# Expanding Xpert MTB/RIF Ultra and lateral flow urine lipoarabinomannan testing for diagnosis of tuberculosis among adults living with HIV admitted to hospitals in Tanzania and Mozambique (EXULTANT): a randomised controlled trial

**DOI:** 10.1016/S1473-3099(26)00133-7

**Published:** 2026-09

**Authors:** Marta Cossa, Robert Ndege, Bindiya Meggi, Chacha Mangu, Vinzeigh Leukes, Issa Sabi, Celso Khosa, Bernard Kivuma, Dinis Nguenha, Friedrich Riess, Carlos Fernández-Escobar, Laura de la Torre-Perez, Patricia Manjate, Maja Weisser, Antonio Machiana, Craysophy Zachariah, Elisa Tagliani, Joanna Ehrlich, Julia Buech, Claudia Schacht, Sergi Sanz, Sunita Singh, Dorcas Mnzava, Samuel G Schumacher, Helen Cox, Leslie Scott, Nyanda Ntinginya, Katharina Kranzer, Morten Ruhwald, Adam Penn-Nicholson, Alberto L Garcia-Basteiro, TB-CAPT Consortium, Vinzeigh Leukes, Adam Penn-Nicholson, Morten Ruhwald, Berra Erkosar, Mikaela Watson, Samuel G Schumacher, Sunita Singh, Bernard Kivuma, Muhuminu Nuru, Francisca Chuwa, Omary Ngome, Neema Shija, Deogratias Bulime, Dorcas Mnzava, Petro Sabuni, Hosiana Temba, Jamali Siru, Jerry Hella, Jonathan Msafiri, Maja Weisser, Mohamed Mbaruku, Mohamed Sasamalo, Alice Leonard, Ambilikile Malango, Annastazia Alexander, Faith Komakoma, Gloria Msigala, Kasmir Johaness, Grace Mhalu, Robert Ndege, Swalehe Masoud, Theonestina Byazukana, Mahmud Mahmud, Lewis Batao, Frederick Haraka, Anange Lwilla, Craysophy Zachariah, Chacha Mangu, Emmanuel Sichone, Lonze Ndelwa, Sara Kiula, Alfred Danda, Regino Mgaya, Bariki Mtafya, Issa Sabi, Last Mwaipopo, Nyanda Elias Ntinginya, Raphael Edom, Willyhelmina Olomi, Delio Elisio, Dinis Nguenha, Edson Mambuque, Joaquim Cossa, Marta Cossa, Neide Gomes, Patricia Manjate, Shilzia Munguambe, Sozinho Acacio, Helio Chiconela, Katia Ribeiro, António Machiana, Bindiya Meggi, Candido Azize Junior, Carla Madeira, Celso Khosa, Claudio Bila, Denise Floripes, Ezequiel Nhamtumbo, Jorge Ribeiro, Sofia Viegas, Alberto Garcia-Basteiro, Belén Saavedra, Pau Benito, Joanna Ehrlich, Laura de la Torre Perez, Sergi Sanz, Friedrich Riess, Katharina Kranzer, Michael Hoelscher, Norbert Heinrich, Sarah Matuku, Tejaswi Apparalarowthu, Leyla Larsson, Maria del Mar Castro Noriega, Claudia M Denkinger, Saima Bashir, Daniela Maria Cirillo, Elisa Tagliani, Federico Di Marco, Virginia Batignani, Akash Malhotra, David Dowdy, Claudia M Schacht, Julia Buech, Caroline Stöhr, Marguerite Massinga Loembé, Pascale Ondoa, Ngobile Ndlovu, Fumbani Brown, Yonas Ghebrekristos, Cindy Hayes, Uilse Vanderwalt, Shareef Abrahams, Puleng Marokane, Mbuti Radebe, Neil Martinson, Anura David, Lesley Scott, Pedro Da Silva, Riffat Munir, Wendy Stevens, Charles Abongomera, Klaus Reither, Leon Stieger, Adrian Brink, Chad Centner, Helen Cox, Judi van Heerden, Mark Nicol, Nchimunya Hapeela, Parveen Brown, Reyhana Salomon, Widaad Zemanay, Tania Dolby

**Affiliations:** a Centro de Investigação em Saúde de Manhiça (CISM), Maputo, Mozambique; b Ifakara Health Institute, Ifakara, Tanzania; c Instituto Nacional de Saúde (INS), Marracuene, Mozambique; d Mbeya Medical Research Centre, National Institute for Medical Research (NIMR), Mbeya, Tanzania; e FIND, Geneva, Switzerland; f Division of Infectious Diseases and Tropical Medicine, LMU University Hospital, LMU Munich, Munich, Germany; g Clinical Research Department, London School of Hygiene & Tropical Medicine, London, UK; h ISGlobal, Hospital Clínic – Universitat de Barcelona, Barcelona, Spain; i Division of Infectious Diseases, University Hospital Basel, Basel, Switzerland; j Swiss Tropical and Public Health Institute, Allschwil, Switzerland; k Emerging Bacterial Pathogens Unit, IRCCS San Raffaele Scientific Institute, Milan, Italy; l LINQ management, Berlin, Germany; m Wits Diagnostic Innovation Hub, Health Sciences Research Office, Faculty of Health Sciences, University of the Witwatersrand, Johannesburg, South Africa; n Division of Medical Microbiology, Department of Pathology, Institute of Infectious Disease and Molecular Medicine, and Wellcome Centre for Infectious Disease Research in Africa, University of Cape Town, Cape Town, South Africa; o Burnet Institute, Melbourne, VIC, Australia

## Abstract

**Background:**

Tuberculosis is the main cause of death among hospitalised people living with HIV. Non-sputum-based diagnostics could improve patient outcomes. The EXULTANT trial aims to evaluate an expanded tuberculosis screening strategy among people with HIV in two African countries with a high tuberculosis and HIV burden.

**Methods:**

This pragmatic, individually randomised controlled superiority trial was conducted across 11 hospitals in Tanzania and Mozambique. We consecutively enrolled adults living with HIV (aged ≥18 years) without an existing tuberculosis diagnosis or recent tuberculosis treatment, within 24 h of admission. The intervention group underwent Xpert MTB/RIF Ultra (Xpert Ultra) testing from sputum, stool, and urine, plus lateral flow urine lipoarabinomannan (LF-LAM) testing (Determine TB LAM Ag assay), irrespective of symptoms. The control group followed standard-of-care, symptom-based, WHO-recommended sputum Xpert Ultra and LF-LAM testing. The primary endpoint was the proportion of participants with microbiologically confirmed tuberculosis starting treatment within 72 h. Secondary endpoints included 8-week all-cause mortality and time to tuberculosis diagnosis. The trial is registered at ClinicalTrials.gov (NCT04568967) and is completed.

**Findings:**

From Sept 25, 2022, to March 15, 2024, we screened 1534 participants, and randomly assigned 1172 (76·6%) to either the intervention group (n=582) or the control group (n=590). At admission, 715 participants (61·0%) were female, 845 (75·4%) were on antiretroviral therapy (ART), and median CD4 count was 232 cells per μL (IQR 87–490). In the control group, 505 (85·6%) had tuberculosis-compatible symptoms and were eligible for sputum Xpert Ultra testing (306 of them [60·6%] provided a sample) and 538 (91·2%) met WHO criteria for LF-LAM testing. In the intention-to-treat analysis, 93 (16·0%) of 582 participants in the intervention group and 90 (15·3%) of 590 in the control group had microbiologically confirmed tuberculosis and started treatment within 72 h (difference 0·7%, 95% CI –3·4 to 4·8, p=0·73). 8-week all-cause mortality was 25·8% (150 of 582) in the intervention group and 28·8% (170 of 590) in the control group (hazard ratio 0·86, 95% CI 0·69 to 1·07, p=0·18). Median time to tuberculosis treatment initiation was 0·98 days (IQR 0·83–1·92) and 0·92 days (0·79–1·86) in the intervention and control groups, respectively (hazard ratio 1·05, 95% CI 0·82 to 1·33, p=0·72).

**Interpretation:**

An expanded screening strategy among people living with HIV admitted to hospital did not increase the proportion of individuals with microbiologically confirmed tuberculosis starting treatment or reduce 8-week mortality.

**Funding:**

EDCTP2 programme, supported by the EU.

## Introduction

Despite a considerable reduction in the incidence of HIV-associated tuberculosis over the last decade, largely due to the widespread implementation of antiretroviral therapy (ART), tuberculosis remains the leading cause of death among people living with HIV.^1^ In 2024 alone, it caused around 150 000 deaths globally.^1^ Diagnostic challenges persist in this population, partially due to non-specific signs and symptoms, higher frequency of extrapulmonary disease, and lower bacillary load typically found in respiratory specimens from people with HIV, which reduces the likelihood of *Mycobacterium tuberculosis* bacteriological confirmation.^2^ In addition, a considerable proportion of hospitalised individuals with HIV with presumptive tuberculosis are unable to produce spontaneous sputum for microbiological testing.^3^ These limitations underscore the urgent need to develop non-sputum based tests and diagnostic algorithms for this population, particularly among those with advanced HIV disease.^4^, ^5^

**Research in context**

**Evidence before this study**

We searched PubMed on Feb 15, 2025, without date or language restrictions, for studies that investigated the effect of tuberculosis molecular tests (Xpert MTB/RIF or Xpert MTB/RIF Ultra) performed in stool or urine or blood among hospitalised people living with HIV. We used different combinations of terms including “stool Xpert”, “blood Xpert”, “urine Xpert”, “urinary Xpert”, and “tuberculosis diagnosis” or “tuberculosis screening” and “admitted” or “hospitalized” and “HIV”. Our search identified 21 relevant studies. Most studies were diagnostic accuracy studies using urine or stool Xpert MTB/RIF or Xpert MTB/RIF Ultra. These studies (14 for urine Xpert and eight for stool Xpert) showed moderate to good diagnostic yield (additional cases detected) compared with sputum-based diagnostic tests. One clinical trial investigated the effect of adding the lateral flow urine lipoarabinomannan (LF-LAM) assay and Xpert MTB/RIF to initial diagnostic screening compared with sputum Xpert MTB/RIF alone. The intervention showed an impact on 56-day mortality in selected groups (low CD4 cell count, severe anaemia, or clinically presumptive tuberculosis), albeit the diagnostic yield was much larger for LF-LAM than for Xpert MTB/RIF (75% *vs* 35%, respectively). We did not identify any interventional study using the more sensitive Xpert cartridge (Xpert Ultra) on either urine, stool, or both, used as part of a screening strategy for tuberculosis diagnosis among unselected people with HIV.

**Added value of this study**

To our knowledge, this is the only randomised trial conducted among hospitalised adults living with HIV to evaluate the effect of an expanded tuberculosis screening strategy (using Xpert Ultra in stool, urine, and sputum) on patient-important outcomes. Despite the promising diagnostic yield previously reported for urine or stool Xpert Ultra in people living with HIV, our pragmatic trial demonstrates that adding molecular testing of these specimens to the WHO-recommended algorithm did not increase the proportion of people with microbiologically confirmed tuberculosis initiating treatment, nor did it reduce 8-week mortality. The trial also highlights that urine and stool were easy-to-collect specimens, while only half of enrolled participants were able to produce sputum. Notably, sputum contributed the fewest positive results overall and had the lowest incremental yield of microbiologically confirmed cases, whether used alone or in combination with other specimen types and tests. Our findings also underscore the central roles of the Determine TB LAM Ag assay, a WHO-recommended test for this population, which provided by far the highest proportion of microbiological confirmation. The additional cases detected by urine and stool Xpert Ultra were comparatively few. However, more than half of the participants with a sole LF-LAM-positive result were not initiated on tuberculosis treatment by 8 weeks, which is a concerning finding requiring further investigation.

**Implications of all the available evidence**

Although the trial shows that both stool and urine Xpert Ultra identify *Mycobacterium tuberculosis* DNA in a considerable number of hospitalised adults living with HIV, our results do not support the use of additional molecular testing for tuberculosis diagnosis among hospitalised people with HIV when LF-LAM testing is readily available. Given the challenges of acquiring sputum among people with HIV and that both urine and stool are easy-to-collect specimens in this population, our data, together with recent articles reporting a good performance of Xpert Ultra in urine and stool, support their use as an alternative to sputum testing, particularly when the latter cannot be obtained. This trial also supports the implementation of simplified tuberculosis testing algorithms among people with HIV admitted to hospital, without the reliance on symptoms or availability of recent CD4 cell count results. The lack of treatment initiation in a high proportion of patients with a single positive LF-LAM result (when all other test results were negative) raises concerns regarding the perceived specificity of the test performed in this population, highlighting the need for more accurate, easy to implement, and clinically trusted next-generation LAM-based diagnostics.

Nearly a decade ago, WHO recommended, for the first time, the use of a point-of-care urine-based test, the Determine TB LAM Ag assay (Abbott, Palatine, IL, USA), for tuberculosis diagnosis among people with HIV.^6^ Clinical trials have shown that lateral flow urine lipoarabinomannan (LF-LAM) testing can decrease all-cause mortality in hospitalised people with HIV, especially among those with low CD4 cell counts.^7^, ^8^ Despite WHO endorsement^9^ and adoption into national tuberculosis guidelines in countries with a high burden of tuberculosis and HIV, LF-LAM remains underused and not widely available.^10^ Promising next-generation LAM-based tests, with improved accuracy and operational characteristics, are currently under clinical evaluation.^11^ The molecular test, Xpert MTB/RIF Ultra (Cepheid, Sunnyvale, CA, USA; hereafter Xpert Ultra) performed on urine has also been evaluated for tuberculosis diagnosis among hospitalised people with HIV in observational studies and clinical trials^8^, ^12^ including a recently published prospective diagnostic study demonstrating its clinical utility in addition to LF-LAM and sputum Xpert Ultra.^13^ Stool has likewise emerged as another non-invasive, easy-to-collect specimen in this population.^14^, ^15^ Already recommended by WHO for tuberculosis detection in children,^16^ recent studies suggest that stool-based Xpert Ultra testing provides substantial diagnostic value in people with HIV, particularly among those with advanced immunosuppression.^17^ Moreover, the collection of additional specimens such as stool has been shown to be both feasible and acceptable to adults and children undergoing tuberculosis evaluation, further supporting its potential integration into routine diagnostic algorithms.^18^

Currently, WHO recommends both LF-LAM testing and sputum Xpert Ultra (or alternative rapid molecular tests) among hospitalised people with HIV.^19^ Non-sputum-based tests with additive diagnostic yield applied universally upon hospital admission could have the potential to increase tuberculosis detection among people with HIV admitted to hospital, accelerate treatment initiation, and ultimately reduce tuberculosis-associated mortality. Thus, we designed a randomised controlled clinical trial to evaluate the impact of an expanded screening package—including universal microbiological testing using sputum, stool, and urine—on the proportion of hospitalised people with HIV who are diagnosed with bacteriologically confirmed tuberculosis and are initiated on treatment within 72 h (3 days) of hospital admission.

## Methods

### Study design and participants

This was a multicentre, pragmatic, unblinded, individually randomised controlled trial implemented in 11 hospitals collaborating with four research institutions in two countries with a high tuberculosis and HIV burden: Tanzania (Ifakara Health Institute [Ifakara and Dar es Salaam] and National Institute for Medical Research [Mbeya]) and Mozambique (Centro de Investigação em Saúde de Manhiça [Manhiça] and Instituto Nacional de Saúde [Maputo]). Hospitals were located in both rural and urban areas across both countries. Details of the participating hospitals and trial design have been previously published.^20^

The trial consecutively enrolled adults (aged ≥18 years) who were admitted to medical wards of participating hospitals with a confirmed HIV diagnosis, irrespective of tuberculosis symptoms. Exclusion criteria included unwillingness to provide consent, currently being on tuberculosis treatment or tuberculosis preventive therapy, having received a tuberculosis diagnosis or tuberculosis treatment or tuberculosis preventive therapy in the 6 months before enrolment, or being transferred from other referral hospitals. Other exclusion criteria were hospital admission due to trauma, acute abdomen, delivery or pregnancy, or planned or scheduled surgery, or living outside the hospital catchment area or having plans to migrate outside the catchment area within 8 weeks after enrolment.^20^

The study protocol, informed consent, and clinical record forms were approved by local and national ethical and regulatory authorities according to the requirements at each country (approval references: 27/CNBS/22 in Mozambique, NIMR/HQ/R.8a/Vol.IX/4083 and TMDA/WEB0022/CTR/0002 in Tanzania). The protocol is available online. The trial is registered at ClinicalTrials.gov (NCT04568967) and is completed.

### Randomisation and masking

The trial randomly allocated admitted patients (1:1) to intervention and control groups. The intervention group consisted of Xpert Ultra testing on expectorated sputum, stool, and urine, and Determine TB LAM Ag testing on urine irrespective of symptoms in the first 72 h (preferably within the first 24 h) after enrolment in the study. Participants allocated to the control group were screened for tuberculosis according to the WHO criteria at the time of study initiation (September, 2022).^19^ WHO criteria included sputum Xpert Ultra testing if the patient had tuberculosis-compatible signs or symptoms (cough, fever, weight loss, or night sweats) and LF-LAM urine test if the patient presented with signs and symptoms of tuberculosis (pulmonary and/or extrapulmonary) or with advanced HIV disease (being seriously ill or with a CD4 count of less than 200 cells per μL). Randomisation was stratified by site using a computer-generated random block size of ten, through a form created in Open Data Kit (ODK) Central. Allocation was predefined in an external database and concealed to study staff. Access to the platform was personalised for study staff at each site and password protected. Each participant was randomly assigned on the date of enrolment (study day 0).

### Procedures

Potential participants were offered participation in the study within 24 h of admission. Informed consent was obtained from participants or, if unable to respond, from the caregiver. After verification of eligibility criteria, relevant sociodemographic and clinical characteristics were collected (using medical records when available).

Specimens were collected as soon after enrolment as possible, preferably within the first 24 h, with the option to collect up to 72 h after enrolment if earlier sampling was not feasible. In case of suspected extrapulmonary tuberculosis, relevant samples including lymph node tissue, pleural, abdominal, and pericardial fluid as well as cerebrospinal fluid were obtained and tested using Xpert Ultra at the discretion of the study clinicians.

Specimens for Xpert Ultra testing were processed as per manufacturer's instructions. Urine Xpert Ultra was performed after cooled centrifugation of urine, and stool Xpert Ultra followed the simple one-step method.^18^ In Mozambique, rectal swabs were optionally collected and tested by Xpert Ultra if stool was unavailable.

During hospitalisation, relevant clinical data, signs and symptoms, diagnoses made, and medical treatments such as initiation of tuberculosis treatment were collected daily using a standardised questionnaire. Participants diagnosed with tuberculosis were managed by the attending clinicians according to established national guidelines for the care of hospitalised people with HIV. The final decision about a diagnosis or treatment initiation was made by the attending clinician. After discharge, two follow-up visits conducted either by phone or in person were scheduled at weeks 4 and 8 (end of study follow-up) after enrolment. These visits aimed to verify participants’ vital status and tuberculosis treatment initiation. When participants or their nominated proxies could not be reached by phone after three attempts, study teams extracted information from the local tuberculosis registers and conducted household visits to ascertain outcomes.

### Outcomes

The primary trial endpoint was the proportion of participants diagnosed with microbiologically confirmed tuberculosis started on tuberculosis treatment within 72 h of enrolment. Microbiologically confirmed tuberculosis was defined as any positive test result including Xpert Ultra tests performed on urine, sputum, stool, or extrapulmonary samples, and LF-LAM urine test. Secondary endpoints included: (1) 8-week all-cause mortality among all participants enrolled; (2) proportion of participants who were diagnosed with microbiologically confirmed tuberculosis or any tuberculosis (including clinically diagnosed patients) and started on treatment at 7 days, 14 days, 4 weeks, and 8 weeks after enrolment; (3) time to tuberculosis diagnosis and time to tuberculosis treatment initiation; (4) proportion of study participants who were able to provide each specimen type (sputum, stool, and urine samples) within 24, 48, and 72 h of admission; and (5) in-hospital and 4-week all-cause mortality among all participants enrolled. Clinically diagnosed tuberculosis was defined as initiation of tuberculosis treatment without microbiological confirmation.

### Statistical analysis

The sample size for the trial made a series of assumptions about the proportion of admitted people with HIV diagnosed with tuberculosis and the potential yield of the different tests used in each study group. Based on data from previous studies using Xpert and LF-LAM in unselected individuals with HIV admitted to hospital, we assumed that in the standard of care group (following WHO diagnostic recommendations in people with HIV admitted to hospital) there would be around 19% of participants with microbiologically confirmed tuberculosis started on treatment. Based on the expected positivity of universal use of urine, sputum, and stool Xpert Ultra and urine LF-LAM, we assumed that 26% of participants in the intervention group would be bacteriologically confirmed and start tuberculosis treatment (7% absolute increase in bacteriological confirmation). Further rationale for the selection of this expected effect of our intervention is found in the protocol publication.^20^ We thus calculated that enrolment of 586 participants per group would provide 80% power to detect this 7% increase in our primary endpoint, assuming a two-sided type I error of 5% and a pretreatment loss to follow-up of 5%.

The trial was analysed following a prespecified statistical analysis plan. The primary analysis used an intention-to-treat principle, including all randomised participants regardless of protocol adherence. Participants were not excluded based on incomplete sample collection or deviations in the timing of study visits. A secondary per-protocol analysis was conducted, including only participants who received protocol-recommended tests with results available within 72 h of enrolment.

All primary and secondary endpoints were compared between study groups. Time-to-endpoint was calculated as the number of days (or hours) from the enrolment date to the occurrence of death, tuberculosis treatment initiation, or diagnosis—depending on the specific endpoint (all-cause mortality, treatment initiation, or tuberculosis diagnosis, respectively). Events were right censored if the endpoint did not occur due to death (except for mortality outcomes), lost to follow-up or planned end of follow-up. In that case, time-to-endpoint was calculated as the time difference from the enrolment date to the last recorded time of follow-up. For descriptive variables, proportions were compared using the χ^2^ test (or Fisher's exact test if at least 20% of cells had expected values <5), and continuous variables were compared with independent *t* tests (or Mann–Whitney *U* test for asymmetrical distributions), with 95% CIs and statistical significance determined at a p value threshold of 0·05. The proportion of rectal swab collection includes only Mozambican participants in the denominator as institutional review board approval was only authorised in this country. Proportion endpoints were reported as risk differences with 95% CIs, estimated with log-binomial regression models adjusted by site, using marginal standardisation with the delta method for obtaining the confidence intervals. Time-to-event outcomes were summarised with Kaplan–Meier curves and compared with hazard ratios with 95% CIs, obtained from Cox regression models adjusted by site. Prespecified subgroup analyses were conducted based on country, sex, study site, baseline CD4 cell count, and baseline viral load. Subgroup analyses included an interaction term that was considered significant if p<0·01.

After concluding the main intention-to-treat and per-protocol analysis, we conducted a limited post-hoc analysis comparing (1) the tuberculosis burden in both groups through applying the same testing criteria in both groups (control group criteria in the intervention group) and (2) the differences in mortality during follow-up among those with single LF-LAM-positive results (individuals in whom all Xpert Ultra tests were negative) who did and did not start tuberculosis treatment.

Study findings are reported in accordance with CONSORT guidelines.^21^ Statistical analyses were conducted using R version 4.3.2. Risk differences and hazard ratios were obtained using the packages ‘risks’ version 0.4.3, and ‘survival’ version 3.8-3, respectively.

### Role of the funding source

The funder of the study had no role in study design, data collection, data analysis, data interpretation, or writing of the report.

## Results

**Figure 1:**
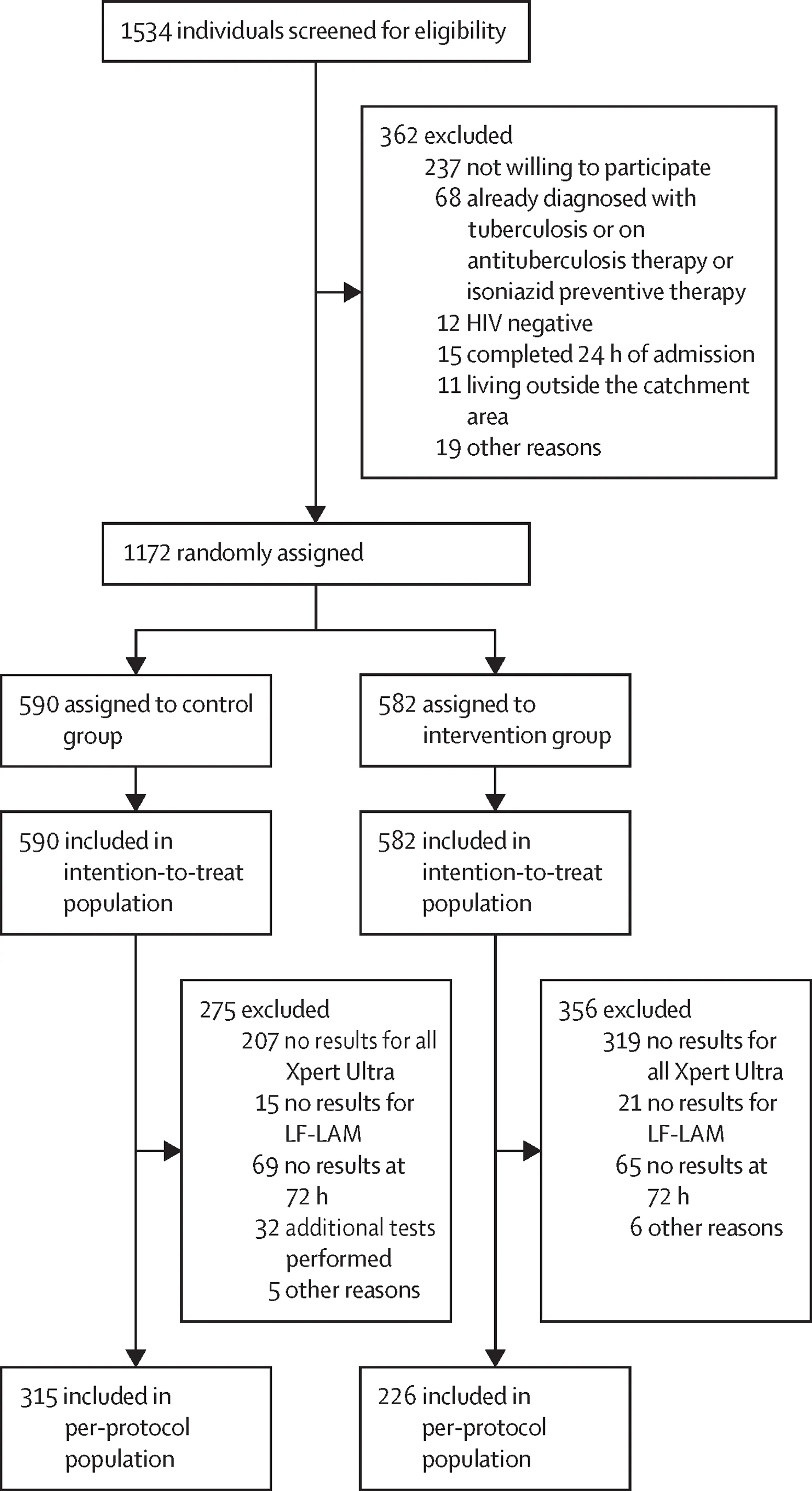
Trial profile Xpert Ultra=Xpert MTB/RIF Ultra. LF-LAM=lateral flow urine lipoarabinomannan (Determine TB LAM Ag assay).

Between Aug 25, 2022, and March 15, 2024, we screened 1534 individuals for eligibility (figure 1). A total of 362 (23·6%) were excluded, leaving 1172 (76·4%) participants in the intention-to-treat population who were randomly assigned 1:1 to the intervention (n=582) or control (n=590) groups. Follow-up time averaged 46·8 days (median 56·1, IQR 42·6–59·1) for the intervention group and 44·4 days (56·0, 29·5–59·0) for the control. Both follow-up visits (week 4 and week 8) were performed in 422 (72·5%) intervention and 407 (69·0%) control participants (p=0·40). Completion of the 8-week follow-up visit was achieved by 425 (73·0%) in the intervention group and 408 (69·2%) participants in the control group (p=0·218).

Baseline characteristics were balanced between the study groups (table 1**,** appendix pp 11–17). Overall, the median age was 44 years (IQR 36–54), and the majority (715 [61·0%] of 1172) were female. Each country enrolled 586 participants. Median time from HIV diagnosis to admission was 4·07 years (IQR 0·1–10·4; n=1049). Among 1121 (95·6%) participants with ART information, 845 (75·4%) were currently on ART (median duration of ART use was 5·0 years [IQR 2·0–10·8]). CD4 cell count results were available for 1122 (95·7%) of 1172 participants. The median CD4 count was 232 cells per μL (IQR 87–490); 310 (27·6%) of 1122 participants had a CD4 count below 100 cells per μL. Viral load measurements were available for 1082 (92·3%) of 1172 participants, with 624 (57·7%) of 1082 having an undetectable viral load (≤150 copies per mL). Almost all participants (1043 [94·6%] of 1103 with available data), had received BCG vaccination, and 332 (29·3%) of 1135 had ever received tuberculosis preventive therapy more than 6 months before trial enrolment. No differences in clinical presentation were observed between intervention and control group (table 1), with 993 (84·7%) participants reporting at least one symptom (cough of any duration in 726 [61·9%]; weight loss in 734 [62·8%]; night sweats in 411 [35·2%], and fever in 325 [27·8%]). Advanced HIV disease—defined as WHO stage 3–4 or CD4 count below 200 cells per μL—was present in 514 (43·9%) of 1172 participants.

**Table 1:** Patient baseline characteristics at enrolment by study group and overall

		**Control group (n=590)**	**Intervention group (n=582)**	**Overall (n=1172)**
Age, years		44·0 (36·0–54·0)	44·0 (36·0–52·0)	44·0 (36·0–53·0)
Sex				
	Female	366 (62·0%)	349 (60·0%)	715 (61·0%)
	Male	224 (38·0%)	233 (40·0%)	457 (39·0%)
Country				
	Mozambique	294 (49·8%)	292 (50·2%)	586 (50·0%)
	Tanzania	296 (50·2%)	290 (49·8%)	586 (50·0%)
Antiretroviral therapy				
	No	136/567 (24·0%)	140/554 (25·3%)	276/1121 (24·6%)
	Yes	431/567 (76·0%)	414/554 (74·7%)	845/1121 (75·4%)
	Missing	23	28	51
WHO clinical stage for HIV/AIDS				
	Stage I	80 (13·6%)	76 (13·1%)	156 (13·3%)
	Stage II	70 (11·9%)	69 (11·9%)	139 (11·9%)
	Stage III	288 (48·8%)	295 (50·7%)	583 (49·7%)
	Stage IV	152 (25·8%)	142 (24·4%)	294 (25·1%)
Any tuberculosis symptom				
	No	85 (14·4%)	94 (16·2%)	179 (15·3%)
	Yes	505 (85·6%)	488 (83·8%)	993 (84·7%)
Cough (any duration)				
	No	222 (37·6%)	224 (38·5%)	446 (38·1%)
	Yes	368 (62·4%)	358 (61·5%)	726 (61·9%)
Cough duration				
	<7 days	116/366 (31·7%)	110/355 (31·0%)	226/721 (31·3%)
	7 days to 2 weeks	133/366 (36·3%)	133/355 (37·5%)	266/721 (36·9%)
	>2 weeks to 2 months	90/366 (24·6%)	92/355 (25·9%)	182/721 (25·2%)
	>2 months	27/366 (7·4%)	20/355 (5·6%)	47/721 (6·5%)
	Missing cough duration	2 (0·5%)	3	5
Producing sputum				
	No	99 (26·9%)	88 (24·6%)	187 (25·8%)
	Yes	269 (73·1%)	270 (75·4%)	539 (74·2%)
Weight loss				
	No	216/589 (36·7%)	219/580 (37·8%)	435/1169 (37·2%)
	Yes	373/589 (63·3%)	361/580 (62·2%)	734/1169 (62·8%)
	Missing	1	2	3
Weight loss, %				
	≥5%	161/373 (43·2%)	152/361 (42·1%)	313/734 (42·6%)
	>5% to 7·5%	120/373 (32·2%)	120/361 (33·2%)	240/734 (32·7%)
	>7·5% to 10%	50/373 (13·4%)	50/361 (13·9%)	100/734 (13·6%)
	>10% to 15%	30/373 (8·0%)	25/361 (6·9%)	55/734 (7·5%)
	>15%	12/373 (3·2%)	14/361 (3·9%)	26/734 (3·5%)
Night sweats				
	No	378/587 (64·4%)	378/580 (65·2%)	756/1167 (64·8%)
	Yes	209/587 (35·6%)	202/580 (34·8%)	411/1167 (35·2%)
	Missing	3	2	5
Fever				
	No	424 (71·9%)	422/581 (72·6%)	846/1171 (72·2%)
	Yes	166 (28·1%)	159/581 (27·4%)	325/1171 (27·8%)
	Missing	0	1	1
Advanced disease (WHO stage 3–4 or CD4 count <200 cells per μL)				
	No	329 (55·8%)	329 (56·5%)	658 (56·1%)
	Yes	261 (44·2%)	253 (43·5%)	514 (43·9%)
Seriously ill				
	No	296 (50·2%)	296 (50·9%)	592 (50·5%)
	Yes	294 (49·8%)	286 (49·1%)	580 (49·5%)
CD4 count category, cells per μL				
	<100	158/563 (28·1%)	152/559 (27·2%)	310/1122 (27·6%)
	100–200	103/563 (18·3%)	101/559 (18·1%)	204/1122 (18·2%)
	≥200	302/563 (53·6%)	306/559 (54·7%)	608/1122 (54·2%)
	Missing	27	23	50
CD4 count, cells per μL		218 (82·0–496)	250 (94·0–488)	232 (87·0–490)
Viral load category				
	Detectable (>150 copies)	233/546 (42·7%)	225/536 (42·0%)	458/1082 (42·3%)
	Undetectable (≥150 copies)	313/546 (57·3%)	311/536 (58·0%)	624/1082 (57·7%)
	Missing	44	46	90
Viral load (thousands of copies) among detectable (>150 copies)		55·6 (2·22–490)	73·7 (1·63–527)	62·8 (1·75–512)

Data are median (IQR), n (%), or n/N (%).

In the control group, 505 (85·6%) of 590 participants had tuberculosis-compatible symptoms and were eligible for sputum Xpert Ultra testing and 538 (91·2%) of 582 met WHO criteria for LF-LAM testing. 306 (51·9%) of 590 participants in the control group (60·6% of the 505 eligible) had a sputum sample collected, compared with 317 (54·5%) of 582 in the intervention group (p=0·40). Urine samples were collected in 564 (96·9%) of 582 participants in the intervention group versus 528 (89·5%) of 590 in the control group (p<0·0001). In the intervention group, spontaneous stool samples were collected in 279 (47·9%) of 582 participants and rectal swabs in 180 (95·2%) of 189 participants in Mozambique for whom spontaneous stool was not collected (table 2). Overall, 459 (78·9%) of 582 participants in the intervention group provided a stool or rectal swab sample.

**Table 2:** Sample collection upon study enrolment

		**Control group (n=590)**	**Intervention group (n=582)**	**p value**
Sputum sample collected		··	··	0·40*
	No	284 (48·1%)	265 (45·5%)	··
	Yes	306 (51·9%)	317 (54·5%)	··
Urine sample collected		··	··	<0·0001*
	No	62 (10·5%)	18 (3·1%)	··
	Yes	528 (89·5%)	564 (96·9%)	··
Stool sample collected		··	··	<0·0001*
	No	586 (99·3%)	123 (21·1%)	··
	Yes	4 (0·7%)	459 (78·9%)	··
Stool sample collection method		··	··	<0·0001*
	Spontaneous stool	4 (0·7%)	279 (47·9%)	··
	Rectal swab	0	180 (30·9%)	··
Ascitic fluid sample collected		··	··	0·42†
	No	589 (99·8%)	579 (99·5%)	··
	Yes	1 (0·2%)	3 (0·5%)	··
Pleural fluid sample collected		··	··	1·0†
	No	588 (99·7%)	580 (99·7%)	··
	Yes	2 (0·3%)	2 (0·3%)	··
Spinal fluid sample collected		··	··	1·0†
	No	564 (95·6%)	562 (96·6%)	··
	Yes	26 (4·4%)	20 (3·4%)	··
Lymph node sample collected		··	··	1·0†
	No	590 (100%)	581 (99·8%)	··
	Yes	0	1 (0·2%)	··

Data are n (%) or p value. Stool, urine, and sputum collection were universally attempted in the intervention group. Urine and sputum in the control group were collected following WHO recommendations (2022). Other extrapulmonary specimens were collected in both groups as per clinical suspicion of attending physician.

* χ^2^ test.

† Fisher's exact test.

At 24 h from enrolment, sputum samples were available in 300 (51·5%) of 582 participants in the intervention group and in 272 (46·1%) of 590 in the control group (p=0·071). For urine samples, 519 (89·2%) of 582 participants in the intervention group had a sample available at 24 h compared with 488 (82·7%) of 590 in the control group (p=0·0020). In the intervention group, spontaneous stool samples were available in 225 (38·7%) participants at 24 h. Similarly, rectal swab samples were collected in 170 (90·0%) of 189 Mozambican participants who did not provide a spontaneous stool sample within 24 h.

Table 3 outlines the availability of test results and diagnostic outcomes. For the Determine TB LAM Ag assay, results were available in 558 (95·9%) of 582 participants in the intervention group and 541 (91·7%) of 590 in the control group; 118 (21·1%) of 558 and 132 (24·4%) of 541 were positive (p=0·22), with indeterminate outcomes observed in three (0·5%) of 558 versus 13 (2·4%) of 541, respectively. Sputum Xpert Ultra results were available for 314 (54·0%) of 582 participants in the intervention group and 295 (50·0%) of 590 in the control group; tuberculosis was microbiologically confirmed in 23 (7·3%) of 314 and 35 (11·9%) of 295, respectively (p=0·077). In the intervention group only, spontaneous stool Xpert Ultra was available in 265 (45·5%) of 582 participants (with *M tuberculosis* detected in 28 [10·6%] of 265), rectal swab Xpert Ultra in 170 (90·0%) of 189 eligible participants (with *M tuberculosis* detected in eight [4·7%] of 170), and urine Xpert Ultra in 541 (93·0%) of 582 (with *M tuberculosis* detected in 38 [7·0%] of 541).

**Figure 2:**
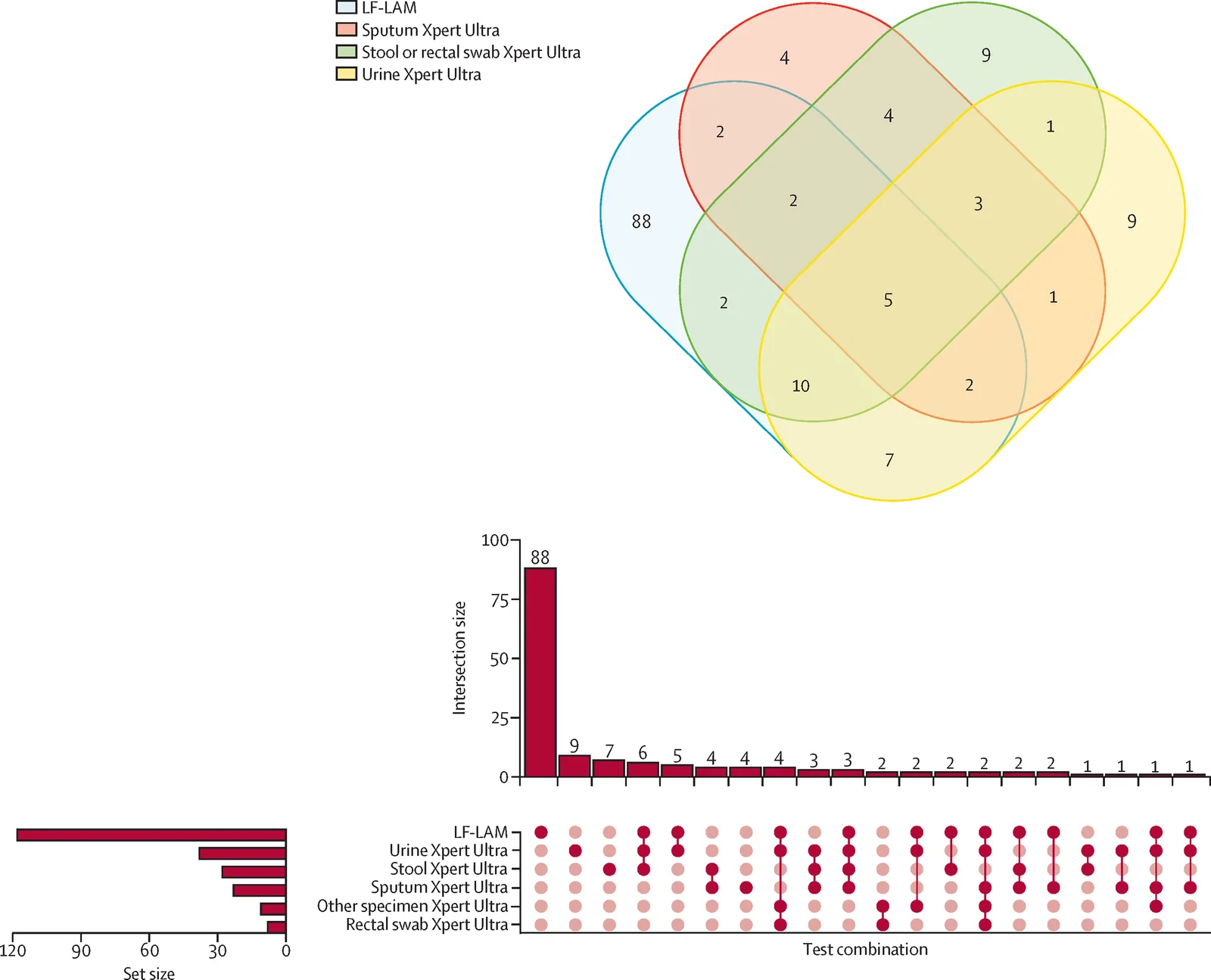
Positive test results in the intervention group, by single test and test combinations Upper plot: Venn diagram of positive results in the intervention group (urine LF-LAM plus sputum, stool [or rectal swab], and urine Xpert Ultra). The size of the diagram is not proportional to the number of positive cases for each test or combination of tests. Lower plot: UpSet plot showing the different scenarios of positive results using all possible test combinations. Set size refers to the total number of positive results for each test. Intersection size refers to the number of positive results for each test alone or in combination with other tests. Xpert Ultra=Xpert MTB/RIF Ultra. LF-LAM=lateral flow urine lipoarabinomannan (Determine TB LAM Ag assay).

In the intervention group, Determine TB LAM Ag had the highest number of positive results (118 [21·1%] of 558 positive among available results), followed by stool Xpert Ultra (28 [10·6%] of 265 positive), sputum Xpert Ultra (23 [7·3%] of 314 positive), urine Xpert Ultra (38 [7·0%] of 541 positive), and rectal swab Xpert Ultra (eight [4·7%] of 170 positive). LF-LAM testing alone accounted for the largest group of unique positive results (88 [59%] of 148 microbiologically confirmed cases; figure 2, table 3). Smaller subsets tested positive exclusively by other methods, including nine of 148 by urine Xpert Ultra (6% of all positives), six of 148 by sputum Xpert Ultra (4% of all positives), and nine of 148 by stool or rectal swab (6% of all positives).

**Table 3:** Diagnostic test results by study group

		**Control group (n=590)**	**Intervention group (n=582)**	**p value**
Number of different test results available		··	··	0·0005*
	0	45 (7·6%)	12 (2·1%)	··
	1	223 (37·8%)	14 (2·4%)	··
	2	307 (52·0%)	85 (14·6%)	··
	3	14 (2·4%)	205 (35·2%)	··
	4	1 (0·2%)	263 (45·2%)	··
	5	0	3 (0·5%)	··
LF-LAM results available		··	··	<0·0001†
	No	49 (8·3%)	24 (4·1%)	··
	Yes	541 (91·7%)	558 (95·9%)	··
LF-LAM result		··	··	0·14*
	Indeterminate	13/541 (2·4%)	3/558 (0·5%)	··
	Negative	396/541 (73·2%)	437/558 (78·3%)	··
	Positive	132/541 (24·4%)	118/558 (21·1%)	··
Sputum Xpert Ultra results available		··	··	<0·0001†
	No	295 (50·0%)	268 (46·0%)	··
	Yes	295 (50·0%)	314 (54·0%)	··
Sputum Xpert Ultra result		··	··	0·14†
	*M tuberculosis* detected	35/295 (11·9%)	23/314 (7·3%)	··
	*M tuberculosis* not detected	260/295 (88·1%)	291/314 (92·7%)	··
Stool Xpert Ultra results available		··	··	<0·0001†
	No	589 (99·8%)	317 (54·5%)	··
	Yes	1 (0·2%)	265 (45·5%)	··
Stool Xpert Ultra result		··	··	1·0*
	*M tuberculosis* detected	0/1	28/265 (10·6%)	··
	*M tuberculosis* not detected	1/1 (100%)	237/265 (89·4%)	··
Urine Xpert Ultra results available		··	··	<0·0001†
	No	578 (98·0%)	41 (7·0%)	··
	Yes	12 (2·0%)	541 (93·0%)	··
Urine Xpert Ultra result		··	··	1·0*
	*M tuberculosis* detected	1/12 (8·3%)	38/541 (7·0%)	··
	*M tuberculosis* not detected	11/12 (91·7%)	503/541 (93·0%)	··
Other specimen Xpert Ultra results available		··	··	<0·0001†
	No	556 (94·2%)	393 (67·5%)	··
	Yes	34 (5·8%)	189 (32·5%)	··
Other specimen Xpert Ultra result		··	··	0·42*
	*M tuberculosis* detected	4/34 (11·8%)	12/189 (6·3%)	··
	*M tuberculosis* not detected	30/34 (88·2%)	177/189 (93·7%)	··
Rectal swab Xpert Ultra results available		··	··	<0·0001†
	No	588 (99·7%)	411 (70·7%)	··
	Yes	2 (0·3%)	170 (29·3%)	··
Rectal swab Xpert Ultra result		··	··	1·0*
	*M tuberculosis* detected	0/2	8/170 (4·7%)	··
	*M tuberculosis* not detected	2/2 (100%)	162/170 (95·3%)	··
Bacteriologically confirmed case (any positive test)		··	··	0·84†
	No	443 (75·1%)	433 (74·4%)	··
	Yes	147 (24·9%)	149 (25·6%)	··

Data are n (%), n/N (%), or p value. Xpert Ultra=Xpert MTB/RIF Ultra. LF-LAM=lateral flow urine lipoarabinomannan (Determine TB LAM Ag assay). Other specimens include lymph node samples or spinal, pleural, or ascitic fluid.

* Fisher's exact test.

† χ^2^ test.

The primary endpoint was reached in 93 (16·0%) of 582 participants in the intervention group and 90 (15·3%) of 590 participants in the control group (difference 0·7%, 95% CI –3·4 to 4·8; p=0·73). The overall proportion of microbiologically confirmed cases was 149 (25·6%) of 582 in the intervention group and 147 (24·9%) of 590 in the control group (p=0·84). Tuberculosis was diagnosed clinically in 46 (7·9%) of 582 participants in the intervention group and 43 (7·3%) of 590 in the control group (p=0·77). When considering all diagnosed tuberculosis (either microbiologically confirmed or clinically diagnosed), 195 (33·5%) of 582 were identified in the intervention group and 190 (32·2%) of 590 in the control group (p=0·68). Tuberculosis treatment was initiated in 144 (24·7%) of 582 participants in the intervention group and 142 (24·1%) of 590 in the control group (p=0·79).

**Figure 3:**
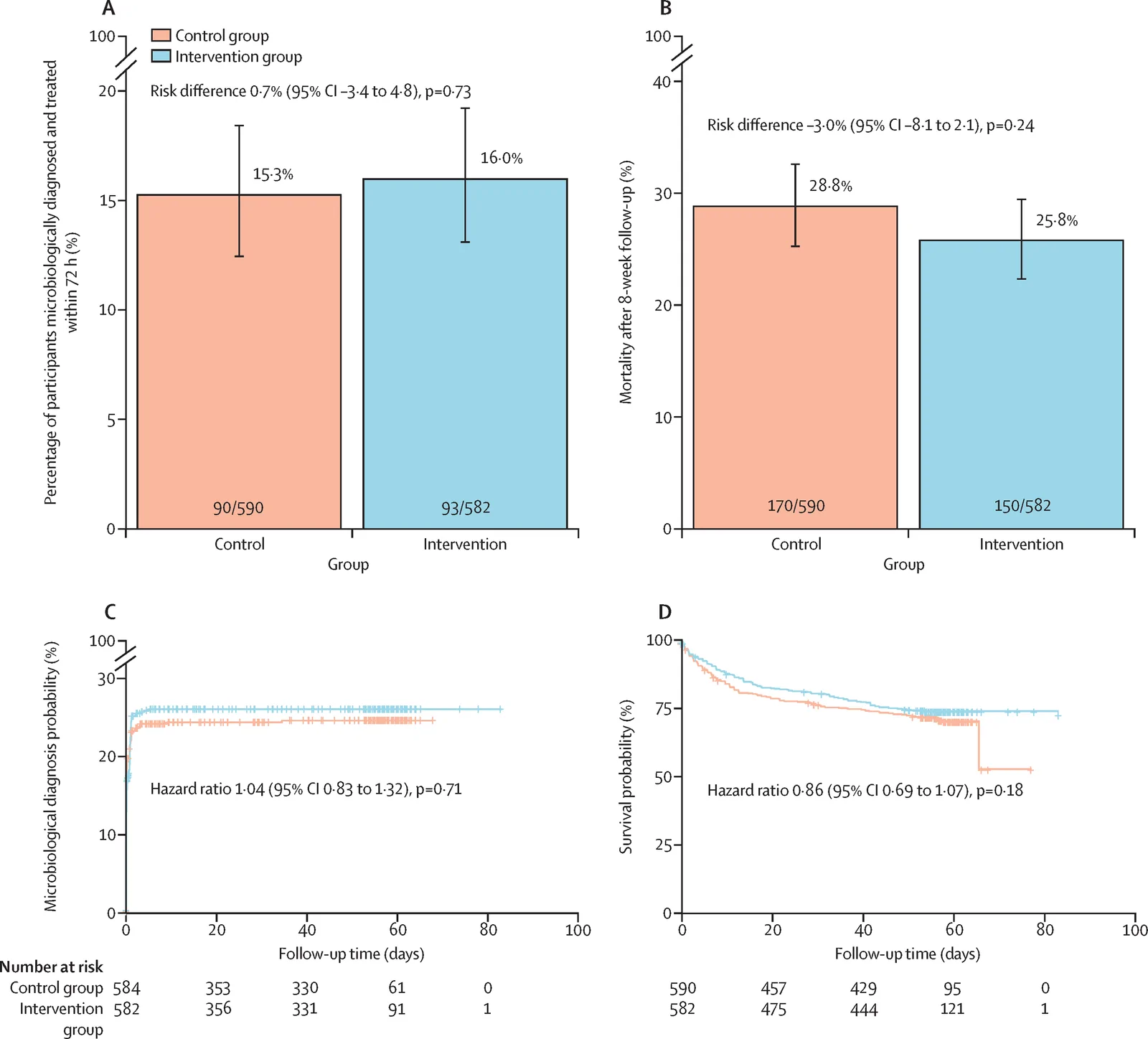
Primary and selected secondary clinical endpoints: early diagnosis and treatment and 8-week mortality (A) Proportion of microbiologically diagnosed individuals started on treatment within 72 h of enrolment by study group. (B) Proportion of deaths at 8 weeks from enrolment by study group. (C) Time to microbiologically confirmed tuberculosis diagnosis. (D) Time to 8-week mortality (survival analysis). In panels A and B, error bars represent 95% CI for proportions; risk differences and p values are adjusted by site. In panels C and D, hazard ratios and p values are from Cox regression adjusted by site.

Secondary endpoints did not show any differences between the study groups. At 8 weeks, overall all-cause mortality was 25·8% (150 of 582) in the intervention group and 28·8% (170 of 590) in the control group (difference –3%, 95% CI –8·1 to 2·1, appendix p 47). Time to mortality in Kaplan–Meier curves (figure 3) was similar between groups (hazard ratio 0·86, 95% CI 0·69 to 1·07, p=0·18). Analyses of 4-week all-cause mortality was also similar between groups (appendix p 74).

Further analyses of diagnosis of microbiologically confirmed tuberculosis at day 7, day 14, week 4, and week 8 did not reveal any differences between the study groups (appendix pp 56–64). Among participants with a positive test result, the median time to tuberculosis diagnosis (first test positivity) was 2·15 h (IQR 0·90–19·4) in the intervention group versus 1·43 h (0·84–5·0) in the control group (hazard ratio 1·04, 95% CI 0·83–1·32, p=0·71). For those treated within 72 h, the median time to tuberculosis treatment initiation was 21·4 h (IQR 19·5–31·1) in the intervention group versus 21·2 h (17·9–28·0) in the control group (p_distribution_=0·52). The median time to tuberculosis treatment initiation overall was 0·98 days (IQR 0·83–1·92) in the intervention group and 0·92 days (0·79–1·86) in the control group (hazard ratio 1·05, 95% CI 0·82 to 1·33, p=0·72).

The subgroup analysis of the primary endpoint—proportion of patients with microbiologically confirmed tuberculosis starting treatment within 72 h—was conducted by CD4 cell count, country, institution, viral load, and sex. 41 (27·0%) of 152 participants in the intervention group with CD4 counts less than 100 cells per μL in the intervention group reached the primary endpoint compared with 44 (27·8%) of 158 in the control group. For participants with CD4 counts of 200 cells per μL or higher, the proportion was 31 (10·1%) of 306 in the intervention and 27 (8·9%) of 302 in the control group (p_interaction_=0·73). By viral load, among those with a detectable viral load (>150 copies per mL), 42 (18·7%) of 225 in the intervention group and 56 (24·0%) of 233 in the control group started treatment within 72 h. For participants with an undetectable viral load (≤150 copies per mL), the corresponding proportions were 42 (13·5%) of 311 in the intervention group and 31 (9·9%) of 313 in the control group (p_interaction_=0·034). No statistically significant differences in the primary endpoint were observed by country or by sex. Similar results were observed in the per-protocol analysis (appendix pp 80–99).

As part of our post-hoc analysis, when applying the same tools and testing criteria used in the control group to the intervention group there was a higher proportion of microbiologically confirmed tuberculosis in the control group (144 [24·4%] of 590) than in the intervention group (126 [21·6%] of 582), although this difference was not statistically significant (p=0·13). The additional tests and specimen types yielded 23 new microbiologically confirmed diagnoses, accounting for approximately 4% of all participants in the intervention group. A limited post-hoc analysis on mortality was done given the observation that 95 participants—representing 48 (32·2%) of 149 and 47 (32·0%) of 147 microbiologically confirmed cases in the intervention and control groups, respectively—had not started treatment by their final study visit (week 8 post-enrolment). Most of these participants (81 [85·3%] of 95) had a single LF-LAM-positive result (37 [77·1%] of 48 in the intervention group; 44 [93·6%] of 47 in the control group). Among participants with at least 3 days of follow-up, no week 8 mortality difference was observed between those with a single positive LF-LAM test started on treatment (25 [23·6%] of 106), and those not initiating treatment (17 [25·4%] of 67), p=0·86. This was the case overall and by trial group (appendix pp 100–103).

## Discussion

Compared with WHO-recommended strategies for hospitalised people with HIV, a diagnostic package incorporating universal molecular screening with Xpert Ultra on stool, urine, and sputum did not increase the proportion of patients initiating tuberculosis treatment within 72 h of admission. This intervention showed no impact on the overall number of tuberculosis diagnoses or on mortality in our study. The modest additional yield in bacteriological confirmation from urine and stool Xpert Ultra testing was outweighed by the high positivity of existing WHO-recommended assays, particularly LF-LAM, resulting in limited incremental diagnostic value from the expanded package. The EXULTANT trial confirms that when current diagnostic strategies are systematically implemented, the burden of tuberculosis among hospitalised people with HIV remains extremely high. Nonetheless, the trial underscores the challenges of sputum collection, with only half of enrolled participants being able to produce a sputum sample, which yielded the fewest positive results overall, contributing the least to additional microbiologically confirmed cases when used alone or in combination with other tests. These findings support the use of urine or stool as alternative specimens for molecular testing when sputum cannot be obtained. Notably, a substantial proportion of participants with sole LF-LAM-positive results were not initiated on tuberculosis treatment, a finding that merits further investigation.

The limited effect of Xpert Ultra testing in multiple specimens or universal diagnostic algorithms on patient-important outcomes—such as mortality or treatment initiation—is consistent with findings from most large diagnostic trials conducted among people under evaluation for tuberculosis over the past 15 years,^22^, ^23^, ^24^, ^25^, ^26^, ^27^, ^28^ including people with HIV admitted to hospital.^8^ To date, only the LF-LAM assay has demonstrated an impact on mortality in two trials, one of which reported benefit only among patients with very low CD4 cell counts.^7^, ^8^ In this trial, 15·3% of participants in the control group and 16·0% in the intervention group were diagnosed with microbiologically confirmed tuberculosis and initiated treatment within 72 h of admission. There might be several reasons why the intervention might have had little impact on specified endpoints. First, a small imbalance in tuberculosis burden between study groups cannot be ruled out. When applying the same testing criteria used in the control group to the intervention group, there was an almost 3% higher bacteriological confirmation in the control compared with the intervention group. The additional tests and specimen types included as part of the trial intervention accounted for approximately 4% increase in bacteriological confirmation in the intervention group. Second, an unexpected and substantial proportion of patients with bacteriologically confirmed tuberculosis (approximately 9%) did not initiate tuberculosis treatment during the study period. In our post-hoc analysis, around 40% of participants with microbiologically confirmed tuberculosis in both groups had not started treatment by their final study visit (week 8 post-enrolment). Most participants were diagnosed on the basis of a single positive LF-LAM result, a WHO-recommended test for this population and included in national guidelines in both Tanzania and Mozambique.^10^ This low proportion initiated on treatment despite a positive LF-LAM result has been reported previously.^29^, ^30^ Several factors might explain this. At the start of the trial, LF-LAM testing was not routinely available at site hospitals, and clinicians—despite being aware of the trial—might not have had sufficient experience or confidence in interpreting results, particularly when other diagnoses were present. Misinterpretation of weak or indeterminate bands is possible but unlikely given staff training, standardised procedure, and double reading of result. Alternatively, the specificity of LF-LAM testing in this population might have been lower than expected. WHO estimated 84% specificity (95% credible interval 48–96)^31^ in unselected hospitalised patients. Given imperfect reference standards and disease heterogeneity, true accuracy is uncertain. The similar mortality between treated and untreated participants with single LF-LAM positive results supports a lower test specificity than previously anticipated. These findings would support (when possible) the performance of complementary urine Xpert Ultra, as recommended in a large multicentre diagnostic study among people with HIV.

This trial incorporated rapid molecular testing of two non-sputum samples which have previously shown additive diagnostic yield when assessed alongside WHO-recommended tests. In the intervention group, 38 participants (26% of all microbiologically confirmed) were urine Xpert Ultra positive, but only nine of these results were additive (ie, not positive with LF-LAM testing or sputum or stool Xpert Ultra). These findings are consistent with the STAMP trial, in which urine Xpert MTB/RIF contributed a modest 6% of all bacteriologically confirmed tuberculosis diagnoses,^8^ comparable to the 7% in our study when stool testing is excluded. Earlier diagnostic accuracy studies among hospitalised people with HIV have reported higher additive yields. In a study by Lawn and colleagues, urine Xpert MTB/RIF identified a greater proportion of cases than LF-LAM testing (59% *vs* 39%, respectively).^12^, ^32^ Similarly, a South African study found higher sensitivity of urine Xpert compared with LF-LAM testing (70% *vs* 45%), when culture from multiple specimens including blood, sputum, and urine were used as reference.^33^ The lower tuberculosis additive yield of urine Xpert testing both in the STAMP and EXULTANT trials probably reflects differences in the immunological profile of participants (with less advanced immunosuppression), as well as variation in study design and local tuberculosis and HIV epidemiology. Stool, like urine, was the sole positive test in nine participants. Although LF-LAM yielded the highest number of positive results overall, stool Xpert Ultra provided the greatest incremental diagnostic yield, with LF-LAM testing identifying 17 additional cases—more than sputum (12) and urine (14) Xpert. A recent study among inpatient and outpatient people with HIV reported that stool Xpert Ultra increased case detection by 23%.^17^ Similarly, a large diagnostic evaluation showed that a novel stool-based quantitative PCR identified 17–21% additional cases compared with sputum Xpert Ultra or sputum culture.^15^

Our results suggest that after LF-LAM testing, stool and sputum tests contribute most to bacterial confirmation (8·5% and 7·3% of bacteriological confirmation in the intervention group, respectively). As many patients are unable to produce sputum, but can provide a stool sample, stool testing yielded the greatest number of additional diagnoses and should be prioritised when sputum is unavailable. Importantly, in settings where LF-LAM testing is not yet widely implemented,^10^ both urine and stool Xpert Ultra can identify a substantial number of additional tuberculosis cases beyond sputum testing alone. Although universal LF-LAM and sputum screening contributed few additional positives in this trial, such strategies simplify diagnostic workup and reduce dependence on CD4 cell count availability or symptom-based screening. This approach is supported by a previous systematic review,^3^ and is now recommended by WHO and incorporated into national guidelines in South Africa.^34^

This trial had several limitations. First, although the proportion of microbiologically confirmed tuberculosis in the control group was higher than anticipated (25% *vs* 19%), the proportion of participants not initiating treatment in this group was unexpectedly high and not accounted for in the sample size. Furthermore, the additional number of cases identified by the systematic testing and expanded sampling was overestimated and the study was underpowered to detect smaller differences between study groups. Second, due to the pragmatic nature of the study,^20^ there were protocol deviations related to the time point of sample collection with some being obtained after 72 h, and/or over-testing in the control group (12 Xpert Ultra in urine and four in stool), clearly pointing at the need for simplification and streamlining of testing algorithms in this population. However, the per-protocol analysis (appendix) suggests the main conclusions would be the same as for the intention-to-treat analysis. Third, in Tanzania the rectal swab procedures were not authorised by the national ethics review board. This lowered the proportion of samples in those not providing stool in Tanzania and thus the true potential of rectal swab sampling in this population is unknown. Despite the yield of rectal swab compared with spontaneous stool being unknown, we have identified a considerable number of positive Xpert Ultra cases through this approach (5% among tested participants in the intervention group). In addition, the high collection rates of rectal swabs among participants who did not provide stool samples support the feasibility of rectal swabs for tuberculosis diagnostics. However, cultural acceptability remains questionable, underscoring the need for further studies on acceptability in such settings. Lastly, the lack of systematic collection of some variables (anaemia, diarrhoea, LAM band intensity, and others) do not allow subanalysis that could have contributed to shed further light on the study results.

To conclude, although this trial did not show an effect in the predefined endpoints, several important lessons emerged from its implementation. First, the findings confirm that urine and stool are feasible and easily collected specimens in hospitalised people with HIV, each contributing additional microbiologically confirmed tuberculosis diagnoses. In contrast, the EXULTANT trial reinforces that sputum is difficult to obtain in this population, yielding the fewest positive results overall. Thus, these results support the use of stool or urine for molecular tuberculosis testing when sputum of any quality is unavailable. Additional studies need to confirm whether stool or urine should be the first specimen to be attempted in this population. Second, the study raises concerns regarding the specificity, interpretation and use of results of the current LF-LAM test. A substantial proportion of participants with single positive LF-LAM results did not initiate tuberculosis treatment, with no observed mortality difference at 8 weeks between those who did and did not start treatment. Such a pattern would be consistent with the interpretation that those not starting treatment might be false positive results. Such findings highlight the urgent need for more accurate, clinician-trusted, bedside-friendly, next-generation urine-based diagnostics.^35^ Despite strong international advocacy for LF-LAM implementation—the only WHO-endorsed point-of-care tuberculosis test—the assay remains underused in many countries with a high tuberculosis and HIV burden.^10^ Limited trust in test results, potentially due to concerns over specificity, might contribute to the suboptimal uptake and impact of this life-saving intervention.

In conclusion, our results suggest that in our study settings the addition of non-sputum Xpert Ultra testing to current diagnostic strategies—including LF-LAM and sputum-based testing—does not substantially increase the number of microbiologically confirmed patients initiated on treatment soon after admission. As such, routine use of this diagnostic package in similar settings might not be justified.

### Contributors

### Data sharing

Data are available on CORA.Repositori de Dades de Recerca, a federated and multidisciplinary data repository of Catalan universities and research centres that follows the FAIR principles and the guidelines of the European Open Science Cloud: https://dataverse.csuc.cat/dataset.xhtml?persistentId=doi:10.34810/data3232.

## Declaration of interests

We declare no competing interests.
