# Supplementary material for: Expanding Xpert MTB/RIF Ultra and lateral flow urine lipoarabinomannan testing for diagnosis of tuberculosis among adults living with HIV admitted to hospitals in Tanzania and Mozambique (EXULTANT): a randomised controlled trial
Source: Lancet Infect Dis. 2026 Sep;26(9):881–93. doi: 10.1016/S1473-3099(26)00133-7 (PMC13490300; doi:10.1016/S1473-3099(26)00133-7)
Supplement: Supplementary file 1 [file mmc1.pdf]

THE LANCET
Infectious Diseases

Supplementary appendix

This appendix formed part of the original submission and has been peer reviewed. We post it as supplied by the authors.

Supplement to: Cossa M, Ndege R, Meggi B, et al. Expanding Xpert MTB/RIF Ultra and lateral flow urine lipoarabinomannan testing for diagnosis of tuberculosis among adults living with HIV admitted to hospitals in Tanzania and Mozambique (EXULTANT): a randomised controlled trial. *Lancet Infect Dis* 2026; published online May 6. https://doi.org/10.1016/S1473-3099(26)00133-7.

Appendix

Expanding Xpert MTB/RIF Ultra and lateral flow urine lipoarabinomannan testing for diagnosis of tuberculosis among adults living with HIV admitted to hospitals in Tanzania and Mozambique (EXULTANT): a randomised controlled trial

Marta Cossa, Robert Ndege, Bindiya Meggi, Chacha Mangu, Vinzeigh Leukes, Issa Sabi, Celso Khosa, Bernard Kivuma, Dinis Nguenha, Friedrich Riess, Carlos Fernández-Escobar, Laura de la Torre-Perez, Patricia Manjate, Maja Weisser, Antonio Machiana, Craysophy Zachariah, Elisa Tagliani, Joanna Ehrlich, Julia Buech, Claudia Schacht, Sergi Sanz, Sunita Singh, Dorcas Mnzava, Samuel G Schumacher, Helen Cox, Leslie Scott, Nyanda Ntinginya, Katharina Kranzer, Morten Ruhwald, Adam Penn-Nicholson, Alberto L Garcia-Basteiro, on behalf of the TB-CAPT Consortium

CONTENT

TB-CAPT Consortium contributors ... 6

Per-Protocol Population criteria ... 7

Exclusion of participants from Per-Protocol Population ... 8

Figure S1. Reasons for excluding participants from Per-Protocol Population 8

Table S1. Reasons for excluding participants from Per-Protocol Population . 9

Sample description ... 11

Table S2. Intention-to-Treat Population ... 11

Table S3. Per-Protocol Population ... 18

Test samples and results ... 24

Table S5. Intention-to-Treat Population ... 24

Test samples and results ... 29

Table S5a. Intention-to-Treat Population. Sample tests and results among asymptomatic participants ... 29

Table S6. Per-Protocol Population ... 31

Outcomes ... 35

Table S7. Intention-to-Treat Population ... 35

Table S8. Per-Protocol-Population ... 39

Outcomes by microbiological confirmation ... 42

Table S9. Intention-to-Treat Population ... 42

Table S10. Per-Protocol-Population ... 43

Main Endpoint: Proportion of microbiologically confirmed TB cases starting treatment within 72 hours of enrolment. ... 45

Figure S2. Intention-to-Treat Population ... 45

Figure S3. Per-Protocol-Population ... 46

Main Secondary Endpoint: 8-week all-cause mortality....47
Figure S4. Intention-to-Treat Population....47
Figure S5. Survival curves in Intention-to-Treat Population....48
Figure S6. Per-Protocol-Population....49
Figure S7. Survival curves in Per-Protocol-Population....50
Secondary Endpoint: Time to treatment initiation....51
Figure S8. Intention-to-Treat Population....51
Figure S9. Per-Protocol-Population....53
Secondary Endpoint: TB diagnosis....54
Figure S10. Intention-to-Treat Population....54
Figure S11. Per-Protocol Population....55
Secondary Endpoint: Diagnosis and treatment day 7, 14, week 4, 8....56
Microbiological Diagnosis and Treatment at Day 7....56
Figure S12. Intention to treat population....56
Figure S13. Per protocol population....58
Microbiological Diagnosis and Treatment at Day 14....59
Figure S14. Intention to treat population....59
Figure S15. Per Protocol population....60
Microbiological Diagnosis and Treatment at Week 4....61
Figure S16. Intention to treat population....61
Figure S17. Per-protocol population....62
Microbiological Diagnosis and Treatment at Week 8....63
Figure S18. Intention to treat population....63
Figure S19. Per-protocol population....64

Secondary Endpoint: Proportion of participants able to provide samples at 24 hours, 48 hours, and 72 or more hours (table S11)........65

Secondary Endpoint: Time to microbiologically confirmed TB diagnosis........68

Table S12. Intention-to-Treat Population........68

Table S13. Per-Protocol-Population........70

Figure S20. Time-to-diagnosis, survival analysis. Intention to treat population........72

Figure S21. Time-to-diagnosis, survival analysis. Per-protocol population...73

Secondary Endpoint: 4-week all-cause mortality........74

Figure S22. Intention-to-Treat Population........74

Figure S23. Per-Protocol-Population........75

Secondary Endpoint: proportion of participants started on treatment without Rif resistance information (only LF-LAM positive)........76

Table S14. Intention-to-Treat Population........76

Figure S24. Intention-to-Treat Population........77

Table S15. Per-Protocol Population........78

Figure S25. Per-Protocol Population........79

Subgroup analysis: Main Endpoint........80

Figure S26. Subgroup Analysis by CD4 Count........80

Figure S27. Subgroup Analysis by Country........82

Figure S28. Subgroup Analysis by Institution........83

Figure S29. Subgroup Analysis by Viral Load........85

Figure S30. Subgroup Analysis by Sex........86

Subgroup analysis: Main Secondary Endpoint........87

Figure S31. Subgroup Analysis by CD4 Count........87

Figure S32. Subgroup Analysis by Country........90

Figure S33. Subgroup Analysis by Institution..92

Figure S34. Subgroup Analysis by Viral Load..96

Figure S35. Subgroup Analysis by Sex..98

Post hoc analyses 100

Figure S36. Mortality of only LF-LAM+ TB cases, with and without treatment 100

Figure S37. Mortality of microbiologically confirmed TB cases, with and without treatment...103

TB-CAPT Consortium contributors

Centro de Investigação em Saúde de Manhiça (CISM), Maputo, Mozambique (M Cossa MD, D Nguenha MD, P Manjate MSc, Prof AL Garcia-Basteiro PhD);

Ifakara Health Institute, Ifakara, Tanzania (R Ndege PhD, B Kivuma MD, D Mnzava BSc, Prof M Weisser MD);

Instituto Nacional de Saúde (INS), Marracuene, Mozambique (B Meggi MD, C Khosa PhD, A Machiana MD);

Mbeya Medical Research Centre, National Institute for Medical Research (NIMR), Mbeya, Tanzania (C Mangu MD, I Sabi PhD, C Zachariah MD, N Ntinginya PhD);

FIND, Geneva, Switzerland (V Leukes PhD, S Singh MSc, S G Schumacher PhD, M Ruhwald PhD, A Penn-Nicholson PhD);

Division of Infectious Diseases and Tropical Medicine, LMU University Hospital, LMU Munich, Munich, Germany (F Riess BSc, Prof K Kranzer);

ISGlobal, Hospital Clínic – Universitat de Barcelona, Barcelona, Spain (C Fernández-Escobar MD, L de la Torre-Perez PhD, J Ehrlich MSc, P Benito, S Sanz MSc, Prof AL Garcia-Basteiro PhD);

Division of Infectious Diseases, University Hospital Basel, Basel, Switzerland (Prof M Weisser PhD);

Swiss Tropical and Public Health Institute, Allschwil, Switzerland (R Ndege PhD, Prof M Weisser MD);

Emerging Bacterial Pathogens Unit, IRCCS San Raffaele Scientific Institute, Milan, Italy (E Tagliani PhD);

LINQ management, Berlin, Germany (J Buech BA, C Schacht PhD);

Wits Diagnostic Innovation Hub, Health Sciences Research Office, Faculty of Health Sciences, University of the Witwatersrand, Johannesburg, South Africa (Prof L Scott PhD);

Division of Medical Microbiology, Department of Pathology, Institute of Infectious Disease and Molecular Medicine, and Wellcome Centre for Infectious Disease Research in Africa, University of Cape Town, Cape Town, South Africa (Prof H Cox PhD);

Burnet Institute, Melbourne, VIC, Australia (Prof H Cox PhD)

Per-Protocol Population criteria

Only participants fulfilling the following criteria were included in the Per-Protocol Population (PPP):

- All samples provided and test results available:
  - Control arm:
    - LF-LAM if symptoms OR advanced disease OR seriously ill.
    - Sputum Ultra if symptoms.
  - Intervention arm:
    - LF-LAM,
    - sputum Ultra,
    - stool Ultra OR rectal swab,
    - urine Ultra.
- No tests performed that were not indicated (in control arm).
- All test results available in less than 72 hours from enrolment.
- No screening failure or major protocol deviation.

Also, for follow-up-related endpoints (e.g., mortality), a modified PPP included the following criteria:

- No follow-up visit missed due to loss to follow-up.

Exception: If the patient died before collection of samples and/or follow-up visits, they still will be included in PPP.

Exclusion of participants from Per-Protocol Population

Figure S1. Reasons for excluding participants from Per-Protocol Population

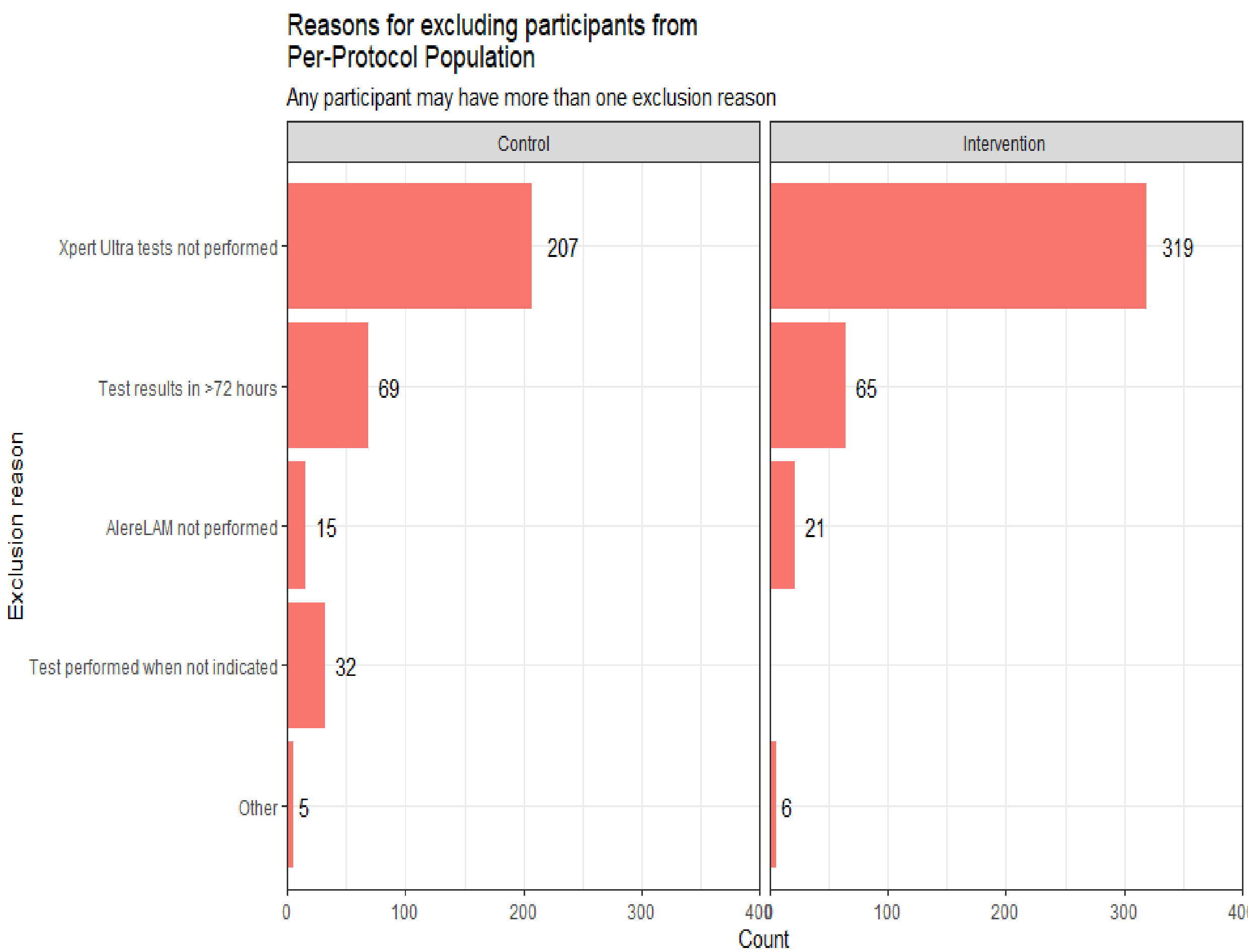

Table S1. Reasons for excluding participants from Per-Protocol Population

	Control (N=590)	Intervention (N=582)	Overall (N=1172)
**Included in Per-Protocol Population**	N=590	N=582	N=1172
Yes	315 (53.4%)	226 (38.8%)	541 (46.2%)
No	275 (46.6%)	356 (61.2%)	631 (53.8%)
**Included in Per-Protocol Population (Mortality analysis)**	N=590	N=582	N=1172
Yes	307 (52.0%)	222 (38.1%)	529 (45.1%)
No	283 (48.0%)	360 (61.9%)	643 (54.9%)
**Study completion**	N=590	N=582	N=1172
No, Death	170 (28.8%)	150 (25.8%)	320 (27.3%)
No, Lost to follow-up	8 (1.4%)	2 (0.3%)	10 (0.9%)
No, Screening failure	1 (0.2%)	1 (0.2%)	2 (0.2%)
No, Withdrawal	3 (0.5%)	4 (0.7%)	7 (0.6%)
Yes, Study completed	408 (69.2%)	425 (73.0%)	833 (71.1%)
**Screening Failure**	N=1	N=1	N=2
**LF-LAM not indicated**	N=19	N=0	N=19
**Stool Ultra not indicated**	N=1	N=0	N=1
**Sputum Ultra not indicated**	N=1	N=0	N=1
**Urine Ultra not indicated**	N=12	N=0	N=12
**Follow-up visits deviation**	N=13	N=10	N=23
**Test results deviation**	N=69	N=65	N=134

	Control (N=590)	Intervention (N=582)	Overall (N=1172)
Results available later than 72 hours from enrolment	69	65	134
**Sputum Ultra deviation**	N=207	N=261	N=468
No sample collected	197 (95.2%)	258 (98.9%)	455 (97.2%)
No valid test performed	10 (4.8%)	3 (1.1%)	13 (2.8%)
**Urine Ultra deviation**	N=0	N=38	N=38
No sample collected	0 (NA%)	14 (36.8%)	14 (36.8%)
No valid test performed	0 (NA%)	24 (63.2%)	24 (63.2%)
**Stool Ultra deviation**	N=0	N=138	N=138
No (stool or rectal swab) sample collected	0 (NA%)	110 (79.7%)	110 (79.7%)
No (stool or rectal swab) test results available	0 (NA%)	28 (20.3%)	28 (20.3%)
**LF-LAM deviation**	N=15	N=21	N=36
No sample collected	13 (86.7%)	14 (66.7%)	27 (75.0%)
No valid test performed	2 (13.3%)	7 (33.3%)	9 (25.0%)

Sample description

Table S2. Intention-to-Treat Population

	Control (N=590)	Intervention (N=582)	Overall (N=1172)
**Age**	N=590	N=582	N=1172
Mean (SD)	45.3 (13.0)	44.6 (12.3)	44.9 (12.7)
Median [Q1, Q3]	44.0 [36.0, 54.0]	44.0 [36.0, 52.0]	44.0 [36.0, 53.0]
**Sex**	N=590	N=582	N=1172
Female	366 (62.0%)	349 (60.0%)	715 (61.0%)
Male	224 (38.0%)	233 (40.0%)	457 (39.0%)
**Country**	N=590	N=582	N=1172
Mozambique	294 (49.8%)	292 (50.2%)	586 (50.0%)
Tanzania	296 (50.2%)	290 (49.8%)	586 (50.0%)
**Ethnic group**	N=590	N=582	N=1172
Black	589 (99.8%)	580 (99.7%)	1169 (99.7%)
South African Coloured (multi-racial ethnicity)	1 (0.2%)	1 (0.2%)	2 (0.2%)
White	0 (0%)	1 (0.2%)	1 (0.1%)
**Highest education level**	N=589	N=580	N=1169
No school	72 (12.2%)	60 (10.3%)	132 (11.3%)
Grade 1-3	44 (7.5%)	48 (8.3%)	92 (7.9%)
Grade 4-7	293 (49.7%)	314 (54.1%)	607 (51.9%)
Grade 8-11	100 (17.0%)	91 (15.7%)	191 (16.3%)
Grade 12	63 (10.7%)	52 (9.0%)	115 (9.8%)
Bachelor Degree	9 (1.5%)	8 (1.4%)	17 (1.5%)
Master or Doctoral Degree	0 (0%)	0 (0%)	0 (0%)
Other	8 (1.4%)	7 (1.2%)	15 (1.3%)
Missing	1 (0.2%)	2 (0.3%)	3 (0.3%)

	Control (N=590)	Intervention (N=582)	Overall (N=1172)
**Employment status**	N=590	N=580	N=1170
Employed	86 (14.6%)	78 (13.4%)	164 (14.0%)
Unemployed	146 (24.7%)	136 (23.4%)	282 (24.1%)
Self-employed	283 (48.0%)	309 (53.3%)	592 (50.6%)
Unable to work (sick/disabled)	22 (3.7%)	14 (2.4%)	36 (3.1%)
Student	6 (1.0%)	5 (0.9%)	11 (0.9%)
House man/house wife	16 (2.7%)	14 (2.4%)	30 (2.6%)
Retired	21 (3.6%)	17 (2.9%)	38 (3.2%)
Other	10 (1.7%)	7 (1.2%)	17 (1.5%)
Missing	0 (0%)	2 (0.3%)	2 (0.2%)
**Employed as a health care worker**	N=590	N=580	N=1170
No	587 (99.5%)	576 (99.3%)	1163 (99.4%)
Yes	3 (0.5%)	4 (0.7%)	7 (0.6%)
Missing	0 (0%)	2 (0.3%)	2 (0.2%)
**Work at a correctional facility**	N=590	N=581	N=1171
No	590 (100%)	576 (99.1%)	1166 (99.6%)
Yes	0 (0%)	5 (0.9%)	5 (0.4%)
Missing	0 (0%)	1 (0.2%)	1 (0.1%)
**Employed as a miner**	N=589	N=581	N=1170
No	581 (98.6%)	572 (98.5%)	1153 (98.5%)
Yes	8 (1.4%)	9 (1.5%)	17 (1.5%)
Missing	1 (0.2%)	1 (0.2%)	2 (0.2%)
**Time from earliest HIV+ result (years)**	N=529	N=520	N=1049
Mean (SD)	5.77 (6.26)	6.35 (6.34)	6.05 (6.30)

	Control (N=590)	Intervention (N=582)	Overall (N=1172)
Median [Q1, Q3]	3.68 [0.0832, 9.54]	4.50 [0.0723, 10.9]	4.07 [0.0750, 10.4]
Missing	61 (10.3%)	62 (10.7%)	123 (10.5%)
**Antiretroviral therapy (ART)**	N=567	N=554	N=1121
No	136 (24.0%)	140 (25.3%)	276 (24.6%)
Yes	431 (76.0%)	414 (74.7%)	845 (75.4%)
Missing	23 (3.9%)	28 (4.8%)	51 (4.4%)
**Received BCG vaccination**	N=552	N=551	N=1103
No	33 (6.0%)	27 (4.9%)	60 (5.4%)
Yes	519 (94.0%)	524 (95.1%)	1043 (94.6%)
Missing	38 (6.4%)	31 (5.3%)	69 (5.9%)
**Cohabitant recently diagnosed with TB**	N=583	N=576	N=1159
No	574 (98.5%)	566 (98.3%)	1140 (98.4%)
Yes	9 (1.5%)	10 (1.7%)	19 (1.6%)
Missing	7 (1.2%)	6 (1.0%)	13 (1.1%)
**Has taken TB preventive therapy**	N=569	N=566	N=1135
No	405 (71.2%)	398 (70.3%)	803 (70.7%)
Yes	164 (28.8%)	168 (29.7%)	332 (29.3%)
Missing	21 (3.6%)	16 (2.7%)	37 (3.2%)
**Taking cotrimoxazole**	N=582	N=573	N=1155
No	515 (88.5%)	496 (86.6%)	1011 (87.5%)
Yes	67 (11.5%)	77 (13.4%)	144 (12.5%)
Missing	8 (1.4%)	9 (1.5%)	17 (1.5%)
**High blood pressure**	N=588	N=579	N=1167
No	460 (78.2%)	483 (83.4%)	943 (80.8%)

	Control (N=590)	Intervention (N=582)	Overall (N=1172)
Yes	128 (21.8%)	96 (16.6%)	224 (19.2%)
Missing	2 (0.3%)	3 (0.5%)	5 (0.4%)
**Diabetes**	N=587	N=581	N=1168
No	567 (96.6%)	566 (97.4%)	1133 (97.0%)
Yes	20 (3.4%)	15 (2.6%)	35 (3.0%)
Missing	3 (0.5%)	1 (0.2%)	4 (0.3%)
**Cardiac disease**	N=589	N=581	N=1170
No	555 (94.2%)	566 (97.4%)	1121 (95.8%)
Yes	34 (5.8%)	15 (2.6%)	49 (4.2%)
Missing	1 (0.2%)	1 (0.2%)	2 (0.2%)
**Epilepsy**	N=590	N=579	N=1169
No	580 (98.3%)	575 (99.3%)	1155 (98.8%)
Yes	10 (1.7%)	4 (0.7%)	14 (1.2%)
Missing	0 (0%)	3 (0.5%)	3 (0.3%)
**Other chronic disease**	N=590	N=582	N=1172
No	538 (91.2%)	545 (93.6%)	1083 (92.4%)
Yes	52 (8.8%)	37 (6.4%)	89 (7.6%)
**Currently smoking**	N=588	N=580	N=1168
No	561 (95.4%)	531 (91.6%)	1092 (93.5%)
Yes	27 (4.6%)	49 (8.4%)	76 (6.5%)
Missing	2 (0.3%)	2 (0.3%)	4 (0.3%)
**Has consumed alcohol in the last 12 months**	N=586	N=578	N=1164
No	447 (76.3%)	412 (71.3%)	859 (73.8%)
Yes	139 (23.7%)	166 (28.7%)	305 (26.2%)
Missing	4 (0.7%)	4 (0.7%)	8 (0.7%)

	Control (N=590)	Intervention (N=582)	Overall (N=1172)
**Karnofsky score**	N=590	N=582	N=1172
Mean (SD)	66.1 (18.6)	66.4 (18.6)	66.3 (18.6)
Median [Q1, Q3]	70.0 [50.0, 80.0]	70.0 [50.0, 80.0]	70.0 [50.0, 80.0]
**WHO clinical stage**	N=590	N=582	N=1172
Stage I	80 (13.6%)	76 (13.1%)	156 (13.3%)
Stage II	70 (11.9%)	69 (11.9%)	139 (11.9%)
Stage III	288 (48.8%)	295 (50.7%)	583 (49.7%)
Stage IV	152 (25.8%)	142 (24.4%)	294 (25.1%)
**BMI category**	N=590	N=582	N=1172
Underweight	114 (19.3%)	126 (21.6%)	240 (20.5%)
Normal weight	336 (56.9%)	337 (57.9%)	673 (57.4%)
Overweight	96 (16.3%)	73 (12.5%)	169 (14.4%)
Obesity	44 (7.5%)	46 (7.9%)	90 (7.7%)
**Any symptom**	N=590	N=582	N=1172
No	85 (14.4%)	94 (16.2%)	179 (15.3%)
Yes	505 (85.6%)	488 (83.8%)	993 (84.7%)
**Cough**	N=590	N=582	N=1172
No	222 (37.6%)	224 (38.5%)	446 (38.1%)
Yes	368 (62.4%)	358 (61.5%)	726 (61.9%)
**Cough duration**	N=366	N=355	N=721
<7 days	116 (31.7%)	110 (31.0%)	226 (31.3%)
7 days-2 weeks	133 (36.3%)	133 (37.5%)	266 (36.9%)
more than 2 weeks-2months	90 (24.6%)	92 (25.9%)	182 (25.2%)
>2 months	27 (7.4%)	20 (5.6%)	47 (6.5%)
Missing	2 (0.5%)	3 (0.8%)	5 (0.7%)

	Control (N=590)	Intervention (N=582)	Overall (N=1172)
**Producing sputum**	N=368	N=358	N=726
No	99 (26.9%)	88 (24.6%)	187 (25.8%)
Yes	269 (73.1%)	270 (75.4%)	539 (74.2%)
**Weight loss**	N=589	N=580	N=1169
No	216 (36.7%)	219 (37.8%)	435 (37.2%)
Yes	373 (63.3%)	361 (62.2%)	734 (62.8%)
Missing	1 (0.2%)	2 (0.3%)	3 (0.3%)
**Weight loss (%)**	N=373	N=361	N=734
<5%	161 (43.2%)	152 (42.1%)	313 (42.6%)
5-7.5%	120 (32.2%)	120 (33.2%)	240 (32.7%)
>7.5-10%	50 (13.4%)	50 (13.9%)	100 (13.6%)
>10-15%	30 (8.0%)	25 (6.9%)	55 (7.5%)
>15%	12 (3.2%)	14 (3.9%)	26 (3.5%)
**Night sweats**	N=587	N=580	N=1167
No	378 (64.4%)	378 (65.2%)	756 (64.8%)
Yes	209 (35.6%)	202 (34.8%)	411 (35.2%)
Missing	3 (0.5%)	2 (0.3%)	5 (0.4%)
**Fever**	N=590	N=581	N=1171
No	424 (71.9%)	422 (72.6%)	846 (72.2%)
Yes	166 (28.1%)	159 (27.4%)	325 (27.8%)
Missing	0 (0%)	1 (0.2%)	1 (0.1%)
**Advanced disease (WHO stage 3/4 or CD4 <200 cells/mm3)**	N=590	N=582	N=1172
No	329 (55.8%)	329 (56.5%)	658 (56.1%)

	Control (N=590)	Intervention (N=582)	Overall (N=1172)
Yes	261 (44.2%)	253 (43.5%)	514 (43.9%)
**Seriously ill**	N=590	N=582	N=1172
No	296 (50.2%)	296 (50.9%)	592 (50.5%)
Yes	294 (49.8%)	286 (49.1%)	580 (49.5%)
**CD4 category**	N=563	N=559	N=1122
<100	158 (28.1%)	152 (27.2%)	310 (27.6%)
100-200	103 (18.3%)	101 (18.1%)	204 (18.2%)
≥200	302 (53.6%)	306 (54.7%)	608 (54.2%)
Missing	27 (4.6%)	23 (4.0%)	50 (4.3%)
**CD4 count**	N=563	N=559	N=1122
Mean (SD)	321 (313)	334 (336)	328 (324)
Median [Q1, Q3]	218 [82.0, 496]	250 [94.0, 488]	232 [87.0, 490]
Missing	27 (4.6%)	23 (4.0%)	50 (4.3%)
**Viral load category**	N=546	N=536	N=1082
Detectable (>150 copies)	233 (42.7%)	225 (42.0%)	458 (42.3%)
Undetectable (≤150 copies)	313 (57.3%)	311 (58.0%)	624 (57.7%)
Missing	44 (7.5%)	46 (7.9%)	90 (7.7%)
**Viral load (thousands of copies), among Detectable (>150 copies)**	N=233	N=225	N=458
Mean (SD)	571 (1340)	1070 (6800)	818 (4860)
Median [Q1, Q3]	55.6 [2.22, 490]	73.7 [1.63, 527]	62.8 [1.75, 512]

Table S3. Per-Protocol Population

	Control (N=315)	Intervention (N=226)	Overall (N=541)
**Age**	N=315	N=226	N=541
Mean (SD)	45.6 (13.0)	43.4 (12.3)	44.7 (12.7)
Median [Q1, Q3]	44.0 [36.0, 54.0]	42.0 [34.3, 51.0]	43.0 [36.0, 53.0]
**Sex**	N=315	N=226	N=541
Female	191 (60.6%)	123 (54.4%)	314 (58.0%)
Male	124 (39.4%)	103 (45.6%)	227 (42.0%)
**Country**	N=315	N=226	N=541
Mozambique	187 (59.4%)	164 (72.6%)	351 (64.9%)
Tanzania	128 (40.6%)	62 (27.4%)	190 (35.1%)
**Ethnic group**	N=315	N=226	N=541
Black	315 (100%)	226 (100%)	541 (100%)
South African Coloured (multi-racial ethnicity)	0 (0%)	0 (0%)	0 (0%)
White	0 (0%)	0 (0%)	0 (0%)
**Highest education level**	N=315	N=226	N=541
No school	46 (14.6%)	27 (11.9%)	73 (13.5%)
Grade 1-3	21 (6.7%)	13 (5.8%)	34 (6.3%)
Grade 4-7	141 (44.8%)	94 (41.6%)	235 (43.4%)
Grade 8-11	55 (17.5%)	58 (25.7%)	113 (20.9%)
Grade 12	43 (13.7%)	31 (13.7%)	74 (13.7%)
Bachelor Degree	6 (1.9%)	1 (0.4%)	7 (1.3%)
Master or Doctoral Degree	0 (0%)	0 (0%)	0 (0%)
Other	3 (1.0%)	2 (0.9%)	5 (0.9%)
**Employment status**	N=315	N=225	N=540
Employed	59 (18.7%)	41 (18.2%)	100 (18.5%)

	Control (N=315)	Intervention (N=226)	Overall (N=541)
Unemployed	63 (20.0%)	46 (20.4%)	109 (20.2%)
Self-employed	156 (49.5%)	117 (52.0%)	273 (50.6%)
Unable to work (sick/disabled)	13 (4.1%)	3 (1.3%)	16 (3.0%)
Student	0 (0%)	4 (1.8%)	4 (0.7%)
House man/house wife	6 (1.9%)	3 (1.3%)	9 (1.7%)
Retired	11 (3.5%)	10 (4.4%)	21 (3.9%)
Other	7 (2.2%)	1 (0.4%)	8 (1.5%)
Missing	0 (0%)	1 (0.4%)	1 (0.2%)
**Employed as a health care worker**	N=315	N=225	N=540
No	313 (99.4%)	223 (99.1%)	536 (99.3%)
Yes	2 (0.6%)	2 (0.9%)	4 (0.7%)
Missing	0 (0%)	1 (0.4%)	1 (0.2%)
**Work at a correctional facility**	N=315	N=226	N=541
No	315 (100%)	224 (99.1%)	539 (99.6%)
Yes	0 (0%)	2 (0.9%)	2 (0.4%)
**Employed as a miner**	N=315	N=226	N=541
No	312 (99.0%)	223 (98.7%)	535 (98.9%)
Yes	3 (1.0%)	3 (1.3%)	6 (1.1%)
**Time from earliest HIV+ result (years)**	N=271	N=205	N=476
Mean (SD)	5.75 (6.48)	5.81 (6.08)	5.77 (6.30)
Median [Q1, Q3]	3.32 [0.0777, 9.49]	3.60 [0.00664, 10.5]	3.52 [0.0367, 9.99]
Missing	44 (14.0%)	21 (9.3%)	65 (12.0%)
**Antiretroviral therapy (ART)**	N=299	N=213	N=512

	Control (N=315)	Intervention (N=226)	Overall (N=541)
No	64 (21.4%)	59 (27.7%)	123 (24.0%)
Yes	235 (78.6%)	154 (72.3%)	389 (76.0%)
Missing	16 (5.1%)	13 (5.8%)	29 (5.4%)
**Received BCG vaccination**	N=299	N=218	N=517
No	15 (5.0%)	10 (4.6%)	25 (4.8%)
Yes	284 (95.0%)	208 (95.4%)	492 (95.2%)
Missing	16 (5.1%)	8 (3.5%)	24 (4.4%)
**Cohabitant recently diagnosed with TB**	N=313	N=224	N=537
No	309 (98.7%)	222 (99.1%)	531 (98.9%)
Yes	4 (1.3%)	2 (0.9%)	6 (1.1%)
Missing	2 (0.6%)	2 (0.9%)	4 (0.7%)
**Has taken TB preventive therapy**	N=302	N=221	N=523
No	214 (70.9%)	158 (71.5%)	372 (71.1%)
Yes	88 (29.1%)	63 (28.5%)	151 (28.9%)
Missing	13 (4.1%)	5 (2.2%)	18 (3.3%)
**Taking cotrimoxazole**	N=311	N=222	N=533
No	269 (86.5%)	184 (82.9%)	453 (85.0%)
Yes	42 (13.5%)	38 (17.1%)	80 (15.0%)
Missing	4 (1.3%)	4 (1.8%)	8 (1.5%)
**High blood pressure**	N=313	N=226	N=539
No	242 (77.3%)	186 (82.3%)	428 (79.4%)
Yes	71 (22.7%)	40 (17.7%)	111 (20.6%)
Missing	2 (0.6%)	0 (0%)	2 (0.4%)
**Diabetes**	N=313	N=226	N=539
No	304 (97.1%)	220 (97.3%)	524 (97.2%)

	Control (N=315)	Intervention (N=226)	Overall (N=541)
Yes	9 (2.9%)	6 (2.7%)	15 (2.8%)
Missing	2 (0.6%)	0 (0%)	2 (0.4%)
**Cardiac disease**	N=315	N=226	N=541
No	297 (94.3%)	222 (98.2%)	519 (95.9%)
Yes	18 (5.7%)	4 (1.8%)	22 (4.1%)
**Epilepsy**	N=315	N=225	N=540
No	312 (99.0%)	225 (100%)	537 (99.4%)
Yes	3 (1.0%)	0 (0%)	3 (0.6%)
Missing	0 (0%)	1 (0.4%)	1 (0.2%)
**Other chronic disease**	N=315	N=226	N=541
No	297 (94.3%)	216 (95.6%)	513 (94.8%)
Yes	18 (5.7%)	10 (4.4%)	28 (5.2%)
**Currently smoking**	N=314	N=226	N=540
No	304 (96.8%)	205 (90.7%)	509 (94.3%)
Yes	10 (3.2%)	21 (9.3%)	31 (5.7%)
Missing	1 (0.3%)	0 (0%)	1 (0.2%)
**Has consumed alcohol in the last 12 months**	N=314	N=226	N=540
No	231 (73.6%)	152 (67.3%)	383 (70.9%)
Yes	83 (26.4%)	74 (32.7%)	157 (29.1%)
Missing	1 (0.3%)	0 (0%)	1 (0.2%)
**Karnofsky score**	N=315	N=226	N=541
Mean (SD)	69.3 (16.4)	70.8 (17.1)	69.9 (16.7)
Median [Q1, Q3]	70.0 [60.0, 80.0]	80.0 [50.0, 80.0]	70.0 [60.0, 80.0]
**WHO clinical stage**	N=315	N=226	N=541
Stage I	36 (11.4%)	21 (9.3%)	57 (10.5%)

	Control (N=315)	Intervention (N=226)	Overall (N=541)
Stage II	27 (8.6%)	18 (8.0%)	45 (8.3%)
Stage III	186 (59.0%)	159 (70.4%)	345 (63.8%)
Stage IV	66 (21.0%)	28 (12.4%)	94 (17.4%)
**BMI category**	N=315	N=226	N=541
Underweight	50 (15.9%)	51 (22.6%)	101 (18.7%)
Normal weight	175 (55.6%)	127 (56.2%)	302 (55.8%)
Overweight	57 (18.1%)	27 (11.9%)	84 (15.5%)
Obesity	33 (10.5%)	21 (9.3%)	54 (10.0%)
**Any symptom**	N=315	N=226	N=541
No	57 (18.1%)	10 (4.4%)	67 (12.4%)
Yes	258 (81.9%)	216 (95.6%)	474 (87.6%)
**Cough**	N=315	N=226	N=541
No	74 (23.5%)	30 (13.3%)	104 (19.2%)
Yes	241 (76.5%)	196 (86.7%)	437 (80.8%)
**Cough duration**	N=241	N=195	N=436
<7 days	78 (32.4%)	61 (31.3%)	139 (31.9%)
7 days-2 weeks	90 (37.3%)	70 (35.9%)	160 (36.7%)
more than 2 weeks-2months	53 (22.0%)	56 (28.7%)	109 (25.0%)
>2 months	20 (8.3%)	8 (4.1%)	28 (6.4%)
Missing	74 (23.5%)	31 (13.7%)	105 (19.4%)
**Producing sputum**	N=241	N=196	N=437
No	29 (12.0%)	17 (8.7%)	46 (10.5%)
Yes	212 (88.0%)	179 (91.3%)	391 (89.5%)
Missing	74 (23.5%)	30 (13.3%)	104 (19.2%)
**Weight loss**	N=315	N=226	N=541

	Control (N=315)	Intervention (N=226)	Overall (N=541)
No	128 (40.6%)	64 (28.3%)	192 (35.5%)
Yes	187 (59.4%)	162 (71.7%)	349 (64.5%)
**Weight loss (%)**	N=187	N=162	N=349
<5%	85 (45.5%)	69 (42.6%)	154 (44.1%)
5-7.5%	58 (31.0%)	58 (35.8%)	116 (33.2%)
>7.5-10%	25 (13.4%)	21 (13.0%)	46 (13.2%)
>10-15%	16 (8.6%)	9 (5.6%)	25 (7.2%)
>15%	3 (1.6%)	5 (3.1%)	8 (2.3%)
Missing	128 (40.6%)	64 (28.3%)	192 (35.5%)
**Night sweats**	N=313	N=226	N=539
No	192 (61.3%)	121 (53.5%)	313 (58.1%)
Yes	121 (38.7%)	105 (46.5%)	226 (41.9%)
Missing	2 (0.6%)	0 (0%)	2 (0.4%)
**Fever**	N=315	N=226	N=541
No	212 (67.3%)	141 (62.4%)	353 (65.2%)
Yes	103 (32.7%)	85 (37.6%)	188 (34.8%)
**Advanced disease (WHO stage 3/4 or CD4 <200 cells/mm3)**	N=315	N=226	N=541
No	173 (54.9%)	130 (57.5%)	303 (56.0%)
Yes	142 (45.1%)	96 (42.5%)	238 (44.0%)
**Seriously ill**	N=315	N=226	N=541
No	160 (50.8%)	116 (51.3%)	276 (51.0%)
Yes	155 (49.2%)	110 (48.7%)	265 (49.0%)
**CD4 category**	N=300	N=220	N=520
<100	82 (27.3%)	63 (28.6%)	145 (27.9%)
100-200	60 (20.0%)	33 (15.0%)	93 (17.9%)

	Control (N=315)	Intervention (N=226)	Overall (N=541)
≥200	158 (52.7%)	124 (56.4%)	282 (54.2%)
Missing	15 (4.8%)	6 (2.7%)	21 (3.9%)
**CD4 count**	N=300	N=220	N=520
Mean (SD)	311 (295)	327 (351)	317 (320)
Median [Q1, Q3]	214 [85.5, 461]	260 [83.3, 462]	230 [84.0, 462]
Missing	15 (4.8%)	6 (2.7%)	21 (3.9%)
**Viral load category**	N=285	N=201	N=486
Detectable (>150 copies)	123 (43.2%)	94 (46.8%)	217 (44.7%)
Undetectable (≤150 copies)	162 (56.8%)	107 (53.2%)	269 (55.3%)
Missing	30 (9.5%)	25 (11.1%)	55 (10.2%)
**Viral load (thousands of copies)**	N=155	N=119	N=274
Mean (SD)	474 (1340)	531 (1380)	499 (1360)
Median [Q1, Q3]	12.5 [0.255, 330]	17.4 [0.272, 305]	12.8 [0.258, 320]
Missing	160 (50.8%)	107 (47.3%)	267 (49.4%)

Test samples and results

Table S5. Intention-to-Treat Population

	Control (N=590)	Intervention (N=582)	p-value
**Sputum sample collected**	N=590	N=582	
No	284 (48.1%)	265 (45.5%)	0.4041[a]
Yes	306 (51.9%)	317 (54.5%)	
**Urine sample collected**	N=590	N=582	
No	62 (10.5%)	18 (3.1%)	<0.0001[a]

	Control (N=590)	Intervention (N=582)	p-value
Yes	528 (89.5%)	564 (96.9%)	
**Stool sample collected**	N=590	N=582	
No	586 (99.3%)	304 (52.2%)	<0.0001[a]
Yes	4 (0.7%)	278 (47.8%)	
**Rectal swab collected**	N=590	N=582	
No	590 (100%)	401 (68.9%)	<0.0001[a]
Yes	0 (0%)	181 (31.1%)	
**Ascitic fluid sample**	N=590	N=582	
No	589 (99.8%)	579 (99.5%)	0.3710[b]
Yes	1 (0.2%)	3 (0.5%)	
**Pleural fluid sample**	N=590	N=582	
No	588 (99.7%)	580 (99.7%)	1.0000[b]
Yes	2 (0.3%)	2 (0.3%)	
**Spinal fluid sample**	N=590	N=582	
No	564 (95.6%)	562 (96.6%)	0.4809[a]
Yes	26 (4.4%)	20 (3.4%)	
**Lymph node sample**	N=590	N=582	
No	590 (100%)	581 (99.8%)	0.4966[b]
Yes	0 (0%)	1 (0.2%)	
**Oral swab sample**	N=590	N=582	
No	590 (100%)	581 (99.8%)	0.4966[b]
Yes	0 (0%)	1 (0.2%)	

	Control (N=590)	Intervention (N=582)	p-value
**Number of different test results available***	N=590	N=582	

	Control (N=590)	Intervention (N=582)	p-value
0	45 (7.6%)	12 (2.1%)	<0.0001[a]
1	223 (37.8%)	14 (2.4%)	
2	307 (52.0%)	85 (14.6%)	
3	14 (2.4%)	204 (35.1%)	
4	1 (0.2%)	264 (45.4%)	
5	0 (0%)	3 (0.5%)	
**LF-LAM results available**	N=590	N=582	
No	49 (8.3%)	24 (4.1%)	0.0045[a]
Yes	541 (91.7%)	558 (95.9%)	
**LF-LAM result**	N=541	N=558	
Indeterminate	13 (2.4%)	3 (0.5%)	0.0123[a]
Negative	396 (73.2%)	437 (78.3%)	
Positive	132 (24.4%)	118 (21.1%)	
Missing	49 (8.3%)	24 (4.1%)	
**LF-LAM result (indeterminate as negative)**	N=541	N=558	
Negative	409 (75.6%)	440 (78.9%)	0.2248[a]
Positive	132 (24.4%)	118 (21.1%)	
Missing	49 (8.3%)	24 (4.1%)	
**Sputum Ultra results available**	N=590	N=582	
No	295 (50.0%)	268 (46.0%)	0.1952[a]
Yes	295 (50.0%)	314 (54.0%)	
**Sputum Ultra result**	N=295	N=314	
MTB detected	35 (11.9%)	23 (7.3%)	0.0769[a]
MTB not detected	260 (88.1%)	291 (92.7%)	
Missing	295 (50.0%)	268 (46.0%)	

	Control (N=590)	Intervention (N=582)	p-value
**Stool Ultra results available**	N=590	N=582	
No	589 (99.8%)	317 (54.5%)	<0.0001[a]
Yes	1 (0.2%)	265 (45.5%)	
**Stool Ultra result**	N=1	N=265	
MTB detected	0 (0%)	28 (10.6%)	1.0000[b]
MTB not detected	1 (100%)	237 (89.4%)	
Missing	589 (99.8%)	317 (54.5%)	
**Urine Ultra results available**	N=590	N=582	
No	578 (98.0%)	41 (7.0%)	<0.0001[a]
Yes	12 (2.0%)	541 (93.0%)	
**Urine Ultra result**	N=12	N=541	
MTB detected	1 (8.3%)	38 (7.0%)	0.5880[b]
MTB not detected	11 (91.7%)	503 (93.0%)	
Missing	578 (98.0%)	41 (7.0%)	
**Other specimen Ultra results available**	N=590	N=582	
No	556 (94.2%)	393 (67.5%)	<0.0001[a]
Yes	34 (5.8%)	189 (32.5%)	
**Other Specimen Ultra result**	N=34	N=189	
MTB detected	4 (11.8%)	12 (6.3%)	0.2563[b]
MTB not detected	30 (88.2%)	177 (93.7%)	
Missing	556 (94.2%)	393 (67.5%)	
**Rectal swab Ultra results available**	N=590	N=582	
No	588 (99.7%)	411 (70.6%)	<0.0001[a]

	Control (N=590)	Intervention (N=582)	p-value
Yes	2 (0.3%)	171 (29.4%)	
**Rectal Swab Ultra result**	N=2	N=171	
MTB detected	0 (0%)	9 (5.3%)	
MTB not detected	2 (100%)	162 (94.7%)	1.0000[b]
Missing	588 (99.7%)	411 (70.6%)	
**Bacteriologically confirmed case (Any positive test)**	N=590	N=582	
No	443 (75.1%)	433 (74.4%)	
Yes	147 (24.9%)	149 (25.6%)	0.8391[a]

[a]: Chi-square test; [b]: Fisher exact test; [c]: t-test; [d]: Mann-Whitney U test.

Test samples and results

Table S5a. Intention-to-Treat Population. Sample tests and results among asymptomatic participants

	Control (N=85)	Intervention (N=94)	Overall (N=179)
Number of different test results available*	N=85	N=94	N=179
0	34 (40.0%)	2 (2.1%)	36 (20.1%)
1	43 (50.6%)	5 (5.3%)	48 (26.8%)
2	7 (8.2%)	25 (26.6%)	32 (17.9%)
3	1 (1.2%)	47 (50.0%)	48 (26.8%)
4	0 (0%)	15 (16.0%)	15 (8.4%)
5	0 (0%)	0 (0%)	0 (0%)
LF-LAM results available	N=85	N=94	N=179
No	35 (41.2%)	6 (6.4%)	41 (22.9%)
Yes	50 (58.8%)	88 (93.6%)	138 (77.1%)
LF-LAM result	N=50	N=88	N=138
Indeterminate	2 (4.0%)	1 (1.1%)	3 (2.2%)
Negative	43 (86.0%)	78 (88.6%)	121 (87.7%)
Positive	5 (10.0%)	9 (10.2%)	14 (10.1%)
LF-LAM result (indeterminate as negative)	N=50	N=88	N=138
Negative	45 (90.0%)	79 (89.8%)	124 (89.9%)
Positive	5 (10.0%)	9 (10.2%)	14 (10.1%)
Sputum Ultra results available	N=85	N=94	N=179
No	84 (98.8%)	82 (87.2%)	166 (92.7%)
Yes	1 (1.2%)	12 (12.8%)	13 (7.3%)
Sputum Ultra result	N=1	N=12	N=13

	Control (N=85)	Intervention (N=94)	Overall (N=179)
MTB detected	0 (0%)	0 (0%)	0 (0%)
MTB not detected	1 (100%)	12 (100%)	13 (100%)
Stool Ultra results available	N=85	N=94	N=179
No	85 (100%)	46 (48.9%)	131 (73.2%)
Yes	0 (0%)	48 (51.1%)	48 (26.8%)
Stool Ultra result	N=0	N=48	N=48
MTB detected	0 (NA%)	2 (4.2%)	2 (4.2%)
MTB not detected	0 (NA%)	46 (95.8%)	46 (95.8%)
Urine Ultra results available	N=85	N=94	N=179
No	83 (97.6%)	6 (6.4%)	89 (49.7%)
Yes	2 (2.4%)	88 (93.6%)	90 (50.3%)
Urine Ultra result	N=2	N=88	N=90
MTB detected	0 (0%)	2 (2.3%)	2 (2.2%)
MTB not detected	2 (100%)	86 (97.7%)	88 (97.8%)
Rectal swab Ultra results available	N=85	N=94	N=179
No	85 (100%)	80 (85.1%)	165 (92.2%)
Yes	0 (0%)	14 (14.9%)	14 (7.8%)
Rectal Swab Ultra result	N=0	N=14	N=14
MTB detected	0 (NA%)	0 (0%)	0 (0%)
MTB not detected	0 (NA%)	14 (100%)	14 (100%)
Bacteriologically confirmed case (Any positive test)	N=85	N=94	N=179
No	79 (92.9%)	83 (88.3%)	162 (90.5%)
Yes	6 (7.1%)	11 (11.7%)	17 (9.5%)

Table S6. Per-Protocol Population

	Control (N=315)	Intervention (N=226)	p-value
**Sputum sample collected**	N=315	N=226	
No	60 (19.0%)	5 (2.2%)	<0.0001[a]
Yes	255 (81.0%)	221 (97.8%)	
**Urine sample collected**	N=315	N=226	
No	32 (10.2%)	1 (0.4%)	<0.0001[a]
Yes	283 (89.8%)	225 (99.6%)	
**Stool sample collected**	N=315	N=226	
No	315 (100%)	105 (46.5%)	<0.0001[a]
Yes	0 (0%)	121 (53.5%)	
**Rectal swab collected**	N=315	N=226	
No	315 (100%)	126 (55.8%)	<0.0001[a]
Yes	0 (0%)	100 (44.2%)	
**Ascitic fluid sample**	N=315	N=226	
No	315 (100%)	225 (99.6%)	0.4177[b]
Yes	0 (0%)	1 (0.4%)	
**Pleural fluid sample**	N=315	N=226	
No	314 (99.7%)	226 (100%)	1.0000[b]
Yes	1 (0.3%)	0 (0%)	
**Spinal fluid sample**	N=315	N=226	
No	310 (98.4%)	223 (98.7%)	1.0000[b]
Yes	5 (1.6%)	3 (1.3%)	
**Lymph node sample**	N=315	N=226	
No	315 (100%)	226 (100%)	1.0000[b]

	Control (N=315)	Intervention (N=226)	p-value
Yes	0 (0%)	0 (0%)	
**Oral swab sample**	N=315	N=226	
No	315 (100%)	226 (100%)	1.0000[b]
Yes	0 (0%)	0 (0%)	

	Control (N=315)	Intervention (N=226)	p-value
**Number of different test results available***	N=315	N=226	
0	32 (10.2%)	0 (0%)	0.0005[b]
1	25 (7.9%)	0 (0%)	
2	254 (80.6%)	3 (1.3%)	
3	4 (1.3%)	3 (1.3%)	
4	0 (0%)	217 (96.0%)	
5	0 (0%)	3 (1.3%)	
**LF-LAM results available**	N=315	N=226	
No	32 (10.2%)	0 (0%)	<0.0001[a]
Yes	283 (89.8%)	226 (100%)	
**LF-LAM result**	N=283	N=226	
Indeterminate	2 (0.7%)	0 (0%)	0.1449[b]
Negative	197 (69.6%)	172 (76.1%)	
Positive	84 (29.7%)	54 (23.9%)	
Missing	32 (10.2%)	0 (0%)	
**LF-LAM result (indeterminate as negative)**	N=283	N=226	
Negative	199 (70.3%)	172 (76.1%)	0.1741[a]
Positive	84 (29.7%)	54 (23.9%)	
Missing	32 (10.2%)	0 (0%)	

	Control (N=315)	Intervention (N=226)	p-value
**Sputum Ultra results available**	N=315	N=226	
No	61 (19.4%)	5 (2.2%)	<0.0001[a]
Yes	254 (80.6%)	221 (97.8%)	
**Sputum Ultra result**	N=254	N=221	
MTB detected	31 (12.2%)	17 (7.7%)	0.1402[a]
MTB not detected	223 (87.8%)	204 (92.3%)	
Missing	61 (19.4%)	5 (2.2%)	
**Stool Ultra results available**	N=315	N=226	
No	315 (100%)	103 (45.6%)	<0.0001[a]
Yes	0 (0%)	123 (54.4%)	
**Stool Ultra result**	N=0	N=123	
MTB detected	0 (NA%)	12 (9.8%)	1.0000[b]
MTB not detected	0 (NA%)	111 (90.2%)	
Missing	315 (100%)	103 (45.6%)	
**Urine Ultra results available**	N=315	N=226	
No	315 (100%)	0 (0%)	<0.0001[a]
Yes	0 (0%)	226 (100%)	
**Urine Ultra result**	N=0	N=226	
MTB detected	0 (NA%)	12 (5.3%)	1.0000[b]
MTB not detected	0 (NA%)	214 (94.7%)	
Missing	315 (100%)	0 (0%)	
**Other specimen Ultra results available**	N=315	N=226	
No	307 (97.5%)	124 (54.9%)	<0.0001[a]

	Control (N=315)	Intervention (N=226)	p-value
Yes	8 (2.5%)	102 (45.1%)	
**Other Specimen Ultra result**	N=8	N=102	
MTB detected	1 (12.5%)	6 (5.9%)	0.4197[b]
MTB not detected	7 (87.5%)	96 (94.1%)	
Missing	307 (97.5%)	124 (54.9%)	
**Rectal swab Ultra results available**	N=315	N=226	
No	315 (100%)	126 (55.8%)	<0.0001[a]
Yes	0 (0%)	100 (44.2%)	
**Rectal Swab Ultra result**	N=0	N=100	
MTB detected	0 (NA%)	5 (5.0%)	1.0000[b]
MTB not detected	0 (NA%)	95 (95.0%)	
Missing	315 (100%)	126 (55.8%)	
**Bacteriologically confirmed case (Any positive test)**	N=315	N=226	
No	219 (69.5%)	157 (69.5%)	1.0000[a]
Yes	96 (30.5%)	69 (30.5%)	

[a]: Chi-square test; [b]: Fisher exact test; [c]: t-test; [d]: Mann-Whitney U test.

Outcomes

Table S7. Intention-to-Treat Population

	Control (N=590)	Intervention (N=582)	p-value
**Bacteriologically confirmed case (Any positive test)**	N=590	N=582	
No	443 (75.1%)	433 (74.4%)	0.8391[a]
Yes	147 (24.9%)	149 (25.6%)	
**Clinically diagnosed TB case (intention to treat with negative tests)**	N=590	N=582	
No	547 (92.7%)	536 (92.1%)	0.7737[a]
Yes	43 (7.3%)	46 (7.9%)	
**TB case (microbiologically confirmed or clinically diagnosed)**	N=590	N=582	
No	400 (67.8%)	387 (66.5%)	0.6802[a]
Yes	190 (32.2%)	195 (33.5%)	
**TB case type**	N=190	N=195	
Clinically diagnosed	43 (22.6%)	46 (23.6%)	0.9187[a]
Microbiologically confirmed	147 (77.4%)	149 (76.4%)	
Missing	400 (67.8%)	387 (66.5%)	
**TB treatment intended**	N=590	N=582	
No	440 (74.6%)	429 (73.7%)	0.7861[a]
Yes	150 (25.4%)	153 (26.3%)	
**TB treatment initiated**	N=590	N=582	
No	448 (75.9%)	438 (75.3%)	0.8409[a]
Yes	142 (24.1%)	144 (24.7%)	

	Control (N=590)	Intervention (N=582)	p-value
**Time to first test positivity (hours)**	N=147	N=149	
Mean (SD)	264 (1270)	10.2 (16.5)	0.1809[d]
Median [Q1, Q3]	1.43 [0.842, 4.98]	2.15 [0.900, 19.4]	
Missing	443 (75.1%)	433 (74.4%)	
**Treated within the first 72 hours**	N=142	N=144	
No	27 (19.0%)	28 (19.4%)	1.0000[a]
Yes	115 (81.0%)	116 (80.6%)	
Missing	448 (75.9%)	438 (75.3%)	
**Time to TB treatment (days)**	N=130	N=135	
Mean (SD)	1.74 (2.34)	2.47 (5.16)	0.3809[d]
Median [Q1, Q3]	0.922 [0.792, 1.86]	0.982 [0.831, 1.92]	
Missing	460 (78.0%)	447 (76.8%)	
**Time to TB treatment, patients treated in ≤72 hours (hours)**	N=115	N=116	
Mean (SD)	24.8 (17.4)	25.7 (16.5)	0.5177[d]
Median [Q1, Q3]	21.2 [17.9, 28.0]	21.4 [19.5, 31.1]	
Missing	475 (80.5%)	466 (80.1%)	
**Time to TB treatment, patients treated in >72 hours (days)**	N=17	N=21	
Mean (SD)	6.43 (3.67)	10.0 (10.2)	0.4325[d]
Median [Q1, Q3]	4.87 [3.87, 7.80]	5.06 [4.84, 9.09]	
Missing	573 (97.1%)	561 (96.4%)	
**Length of hospitalization (days)**	N=568	N=558	

	Control (N=590)	Intervention (N=582)	p-value
Mean (SD)	6.31 (9.55)	5.86 (8.40)	0.4689[d]
Median [Q1, Q3]	4.12 [2.14, 7.15]	4.09 [2.08, 7.18]	
Missing	22 (3.7%)	24 (4.1%)	
**Death at week 8 of follow-up**	N=590	N=582	
No	420 (71.2%)	432 (74.2%)	0.2702[a]
Yes	170 (28.8%)	150 (25.8%)	
**Follow-up time (days)**	N=590	N=582	
Mean (SD)	44.4 (22.0)	46.8 (20.4)	0.0435[d]
Median [Q1, Q3]	56.0 [29.5, 59.0]	56.1 [42.6, 59.1]	
**Study completion**	N=590	N=582	
No, Death	170 (28.8%)	150 (25.8%)	0.2159[b]
No, Lost to follow-up	8 (1.4%)	2 (0.3%)	
No, Screening failure	1 (0.2%)	1 (0.2%)	
No, Withdrawal	3 (0.5%)	4 (0.7%)	
Yes, Study completed	408 (69.2%)	425 (73.0%)	
**Week 4 visit performed**	N=590	N=582	
No, patient died before	143 (24.2%)	116 (19.9%)	0.1954[a]
No, patient was lost to follow-up or left study	9 (1.5%)	8 (1.4%)	
Yes	438 (74.2%)	458 (78.7%)	
**Week 8 visit performed**	N=590	N=582	
No, patient died before	170 (28.8%)	150 (25.8%)	0.2395[a]
No, patient was lost to follow-up or left study	12 (2.0%)	7 (1.2%)	
Yes	408 (69.2%)	425 (73.0%)	

	Control (N=590)	Intervention (N=582)	p-value
**Both follow-up visits performed**	N=590	N=582	
No, patient died before	170 (28.8%)	150 (25.8%)	0.3949[a]
No, patient was lost to follow-up or left study	13 (2.2%)	10 (1.7%)	
Yes	407 (69.0%)	422 (72.5%)	

[a]: Chi-square test; [b]: Fisher exact test; [c]: t-test; [d]: Mann-Whitney U test.

Table S8. Per-Protocol-Population

	Control (N=315)	Intervention (N=226)	p-value
**Bacteriologically confirmed case (Any positive test)**	N=315	N=226	
No	219 (69.5%)	157 (69.5%)	1.0000[a]
Yes	96 (30.5%)	69 (30.5%)	
**Clinically diagnosed TB case (intention to treat with negative tests)**	N=315	N=226	
No	289 (91.7%)	196 (86.7%)	0.0806[a]
Yes	26 (8.3%)	30 (13.3%)	
**TB case (microbiologically confirmed or clinically diagnosed)**	N=315	N=226	
No	193 (61.3%)	127 (56.2%)	0.2732[a]
Yes	122 (38.7%)	99 (43.8%)	
**TB case type**	N=122	N=99	
Clinically diagnosed	26 (21.3%)	30 (30.3%)	0.1698[a]
Microbiologically confirmed	96 (78.7%)	69 (69.7%)	
Missing	193 (61.3%)	127 (56.2%)	
**TB treatment intended**	N=315	N=226	
No	210 (66.7%)	145 (64.2%)	0.6074[a]
Yes	105 (33.3%)	81 (35.8%)	
**TB treatment initiated**	N=315	N=226	
No	216 (68.6%)	151 (66.8%)	0.7352[a]
Yes	99 (31.4%)	75 (33.2%)	
**Time to first test positivity (hours)**	N=96	N=69	

	Control (N=315)	Intervention (N=226)	p-value
Mean (SD)	5.07 (8.97)	6.29 (9.70)	0.3372[d]
Median [Q1, Q3]	1.24 [0.750, 3.10]	1.40 [0.833, 5.55]	
Missing	219 (69.5%)	157 (69.5%)	
**Treated within the first 72 hours**	N=99	N=75	
No	16 (16.2%)	12 (16.0%)	1.0000[a]
Yes	83 (83.8%)	63 (84.0%)	
Missing	216 (68.6%)	151 (66.8%)	
**Time to TB treatment (days)**	N=93	N=69	
Mean (SD)	1.73 (2.42)	2.18 (4.05)	0.3896[d]
Median [Q1, Q3]	0.906 [0.809, 1.83]	0.972 [0.833, 1.90]	
Missing	222 (70.5%)	157 (69.5%)	
**Time to TB treatment, patients treated in ≤72 hours (hours)**	N=83	N=63	
Mean (SD)	24.9 (17.5)	28.2 (17.8)	0.3213[d]
Median [Q1, Q3]	21.2 [18.9, 27.0]	21.5 [19.8, 40.4]	
Missing	232 (73.7%)	163 (72.1%)	
**Time to TB treatment, patients treated in >72 hours (days)**	N=11	N=8	
Mean (SD)	6.90 (4.04)	9.75 (8.96)	0.7168[d]
Median [Q1, Q3]	5.86 [3.93, 7.36]	5.86 [4.86, 9.25]	
Missing	304 (96.5%)	218 (96.5%)	
**Length of hospitalization (days)**	N=311	N=219	

	Control (N=315)	Intervention (N=226)	p-value
Mean (SD)	6.37 (7.72)	6.07 (5.68)	0.4112[d]
Median [Q1, Q3]	4.14 [2.19, 7.14]	4.67 [2.15, 7.94]	
Missing	4 (1.3%)	7 (3.1%)	
**Death at week 8 of follow-up**	N=315	N=226	
No	231 (73.3%)	173 (76.5%)	0.4545[a]
Yes	84 (26.7%)	53 (23.5%)	
**Follow-up time (days)**	N=315	N=226	
Mean (SD)	46.4 (21.2)	48.6 (19.6)	0.1502[d]
Median [Q1, Q3]	56.1 [38.1, 59.4]	57.0 [52.9, 60.2]	
**Study completion**	N=315	N=226	
No, Death	84 (26.7%)	53 (23.5%)	0.3408[b]
No, Lost to follow-up	6 (1.9%)	1 (0.4%)	
No, Screening failure	0 (0%)	0 (0%)	
No, Withdrawal	1 (0.3%)	1 (0.4%)	
Yes, Study completed	224 (71.1%)	171 (75.7%)	
**Week 4 visit performed**	N=315	N=226	
No, patient died before	69 (21.9%)	43 (19.0%)	0.7311[b]
No, patient was lost to follow-up or left study	5 (1.6%)	3 (1.3%)	
Yes	241 (76.5%)	180 (79.6%)	
**Week 8 visit performed**	N=315	N=226	
No, patient died before	84 (26.7%)	53 (23.5%)	0.3127[a]
No, patient was lost to follow-up or left study	7 (2.2%)	2 (0.9%)	
Yes	224 (71.1%)	171 (75.7%)	

	Control (N=315)	Intervention (N=226)	p-value
**Both follow-up visits performed**	N=315	N=226	
No, patient died before	84 (26.7%)	53 (23.5%)	0.5551[a]
No, patient was lost to follow-up or left study	8 (2.5%)	4 (1.8%)	
Yes	223 (70.8%)	169 (74.8%)	

[a]: Chi-square test; [b]: Fisher exact test; [c]: t-test; [d]: Mann-Whitney U test

Outcomes by microbiological confirmation

Table S9. Intention-to-Treat Population

	Control		Intervention	
	Clinical diagnosis (N=43)	Lab-confirmed (N=147)	Clinical diagnosis (N=46)	Lab-confirmed (N=149)
**TB treatment intended**	N=43	N=147	N=46	N=149
No	0 (0%)	40 (27.2%)	0 (0%)	42 (28.2%)
Yes	43 (100%)	107 (72.8%)	46 (100%)	107 (71.8%)
**TB treatment initiated**	N=43	N=147	N=46	N=149
No	1 (2.3%)	47 (32.0%)	3 (6.5%)	48 (32.2%)
Yes	42 (97.7%)	100 (68.0%)	43 (93.5%)	101 (67.8%)
**Treated within the first 72 hours**	N=42	N=100	N=43	N=101
No	17 (40.5%)	10 (10.0%)	21 (48.8%)	7 (6.9%)
Yes	25 (59.5%)	90 (90.0%)	22 (51.2%)	94 (93.1%)
Missing	1 (2.3%)	47 (32.0%)	3 (6.5%)	48 (32.2%)
**Time to TB treatment (days)**	N=33	N=97	N=35	N=100

	Control		Intervention	
	Clinical diagnosis (N=43)	Lab-confirmed (N=147)	Clinical diagnosis (N=46)	Lab-confirmed (N=149)
Mean (SD)	2.92 (3.67)	1.34 (1.48)	3.71 (6.33)	2.03 (4.64)
Median [Q1, Q3]	1.70 [0.704, 3.87]	0.899 [0.794, 1.58]	1.90 [0.861, 4.85]	0.917 [0.810, 1.59]
Missing	10 (23.3%)	50 (34.0%)	11 (23.9%)	49 (32.9%)
**Death at week 8 of follow-up**	N=43	N=147	N=46	N=149
No	32 (74.4%)	97 (66.0%)	31 (67.4%)	109 (73.2%)
Yes	11 (25.6%)	50 (34.0%)	15 (32.6%)	40 (26.8%)

Table S10. Per-Protocol-Population

	Control		Intervention	
	Clinical diagnosis (N=26)	Lab-confirmed (N=96)	Clinical diagnosis (N=30)	Lab-confirmed (N=69)
**TB treatment intended**	N=26	N=96	N=30	N=69
No	0 (0%)	17 (17.7%)	0 (0%)	18 (26.1%)
Yes	26 (100%)	79 (82.3%)	30 (100%)	51 (73.9%)
**TB treatment initiated**	N=26	N=96	N=30	N=69
No	0 (0%)	23 (24.0%)	2 (6.7%)	22 (31.9%)
Yes	26 (100%)	73 (76.0%)	28 (93.3%)	47 (68.1%)
**Treated within the first 72 hours**	N=26	N=73	N=28	N=47
No	11 (42.3%)	5 (6.8%)	9 (32.1%)	3 (6.4%)
Yes	15 (57.7%)	68 (93.2%)	19 (67.9%)	44 (93.6%)
Missing	0 (0%)	23 (24.0%)	2 (6.7%)	22 (31.9%)

	Control		Intervention	
	Clinical diagnosis (N=26)	Lab-confirmed (N=96)	Clinical diagnosis (N=30)	Lab-confirmed (N=69)
**Time to TB treatment (days)**	N=22	N=71	N=23	N=46
Mean (SD)	3.59 (4.28)	1.16 (0.881)	2.01 (1.86)	2.26 (4.80)
Median [Q1, Q3]	1.79 [0.828, 5.61]	0.892 [0.802, 1.15]	0.894 [0.838, 2.84]	0.982 [0.830, 1.88]
Missing	4 (15.4%)	25 (26.0%)	7 (23.3%)	23 (33.3%)
**Death at week 8 of follow-up**	N=26	N=96	N=30	N=69
No	20 (76.9%)	63 (65.6%)	20 (66.7%)	53 (76.8%)
Yes	6 (23.1%)	33 (34.4%)	10 (33.3%)	16 (23.2%)

Main Endpoint: Proportion of microbiologically confirmed TB cases starting treatment within 72 hours of enrolment.

Figure S2. Intention-to-Treat Population

Group	Numerator	Denominator	Proportion	Risk Difference	95% Confidence interval	p value
Control	90	590	15.3	0.7%	(-3.4% to 4.8%)	0.7272
Intervention	93	582	16.0			

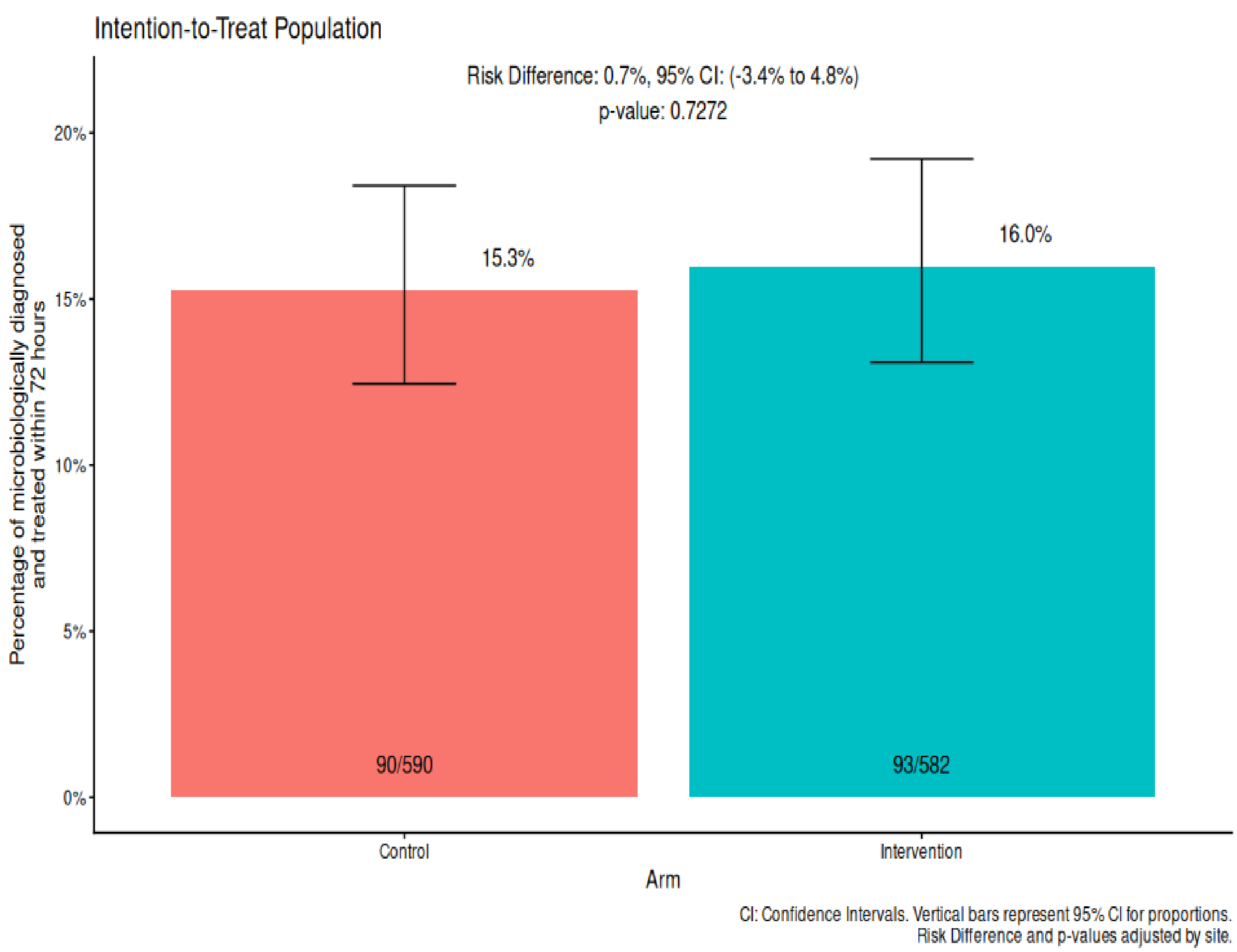

Figure S3. Per-Protocol-Population

Group	Numerator	Denominator	Proportion	Risk Difference	95% Confidence interval	p value
Control	68	315	21.6	-3.0%	(-9.9% to 3.8%)	0.3885
Intervention	44	226	19.5			

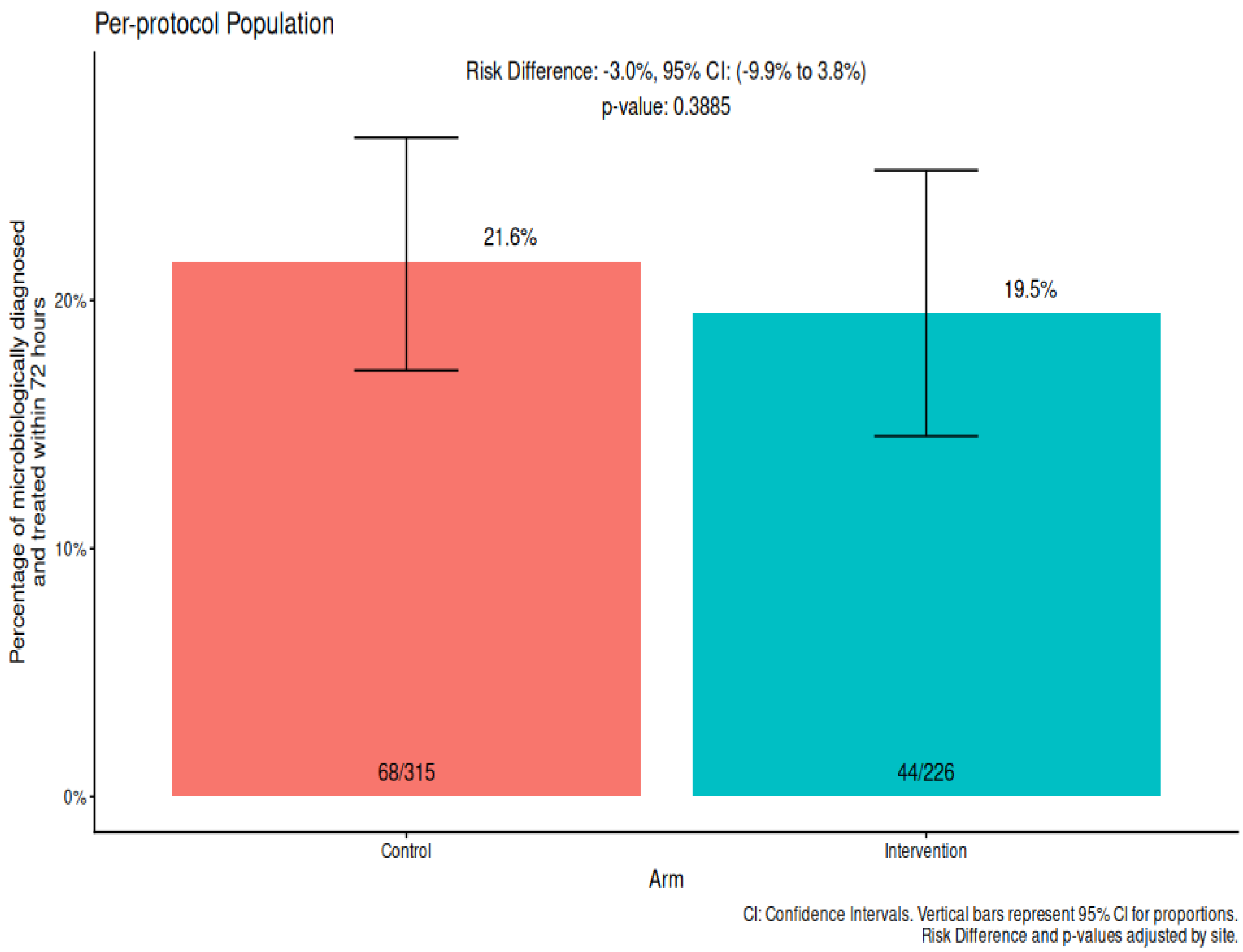

Main Secondary Endpoint: 8-week all-cause mortality

Figure S4. Intention-to-Treat Population

Group	Numerator	Denominator	Proportion	Risk Difference	95% Confidence interval	p value
Control	170	590	28.8	-3.0%	(-8.1% to 2.1%)	0.2428
Intervention	150	582	25.8			

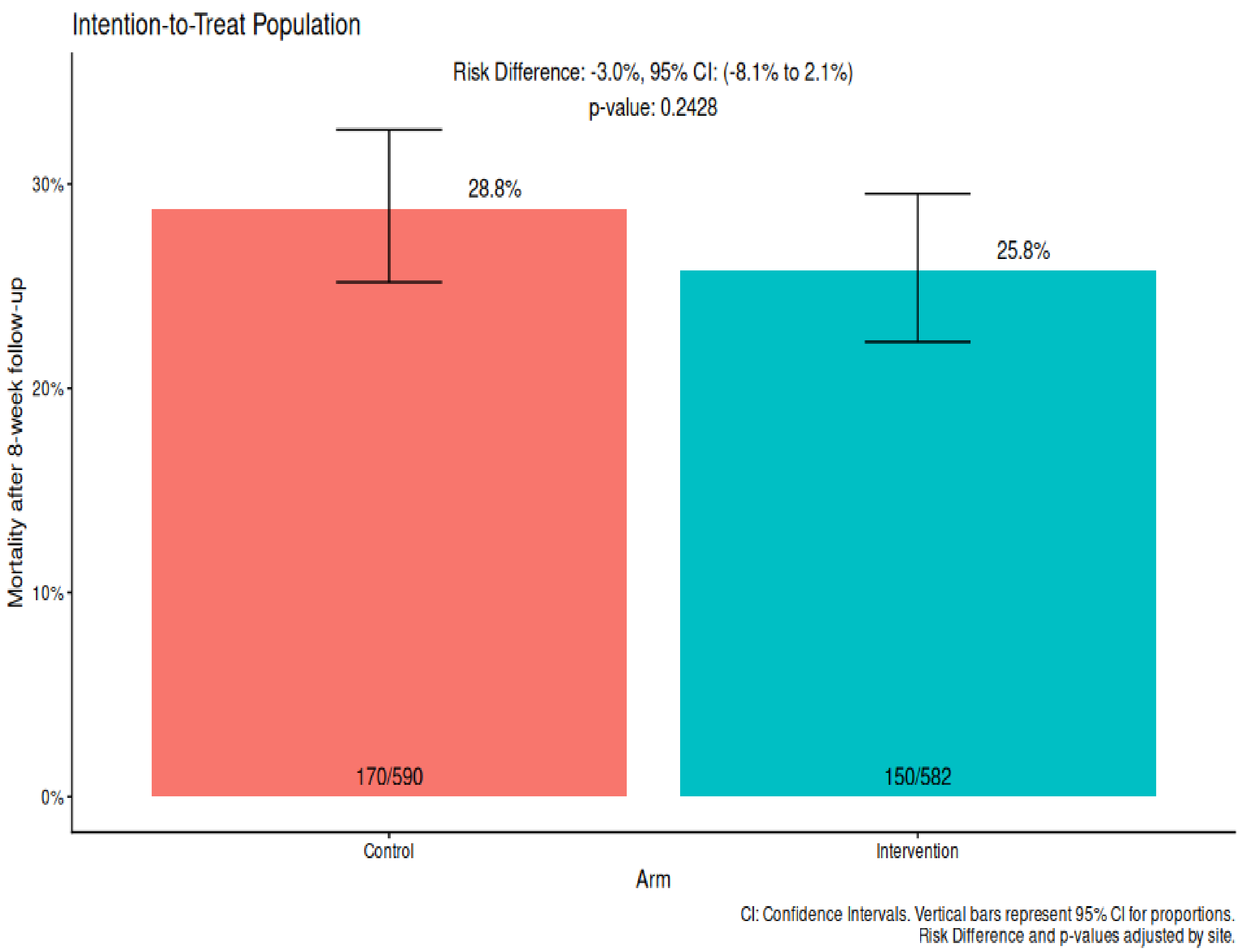

Figure S5. Survival curves in Intention-to-Treat Population

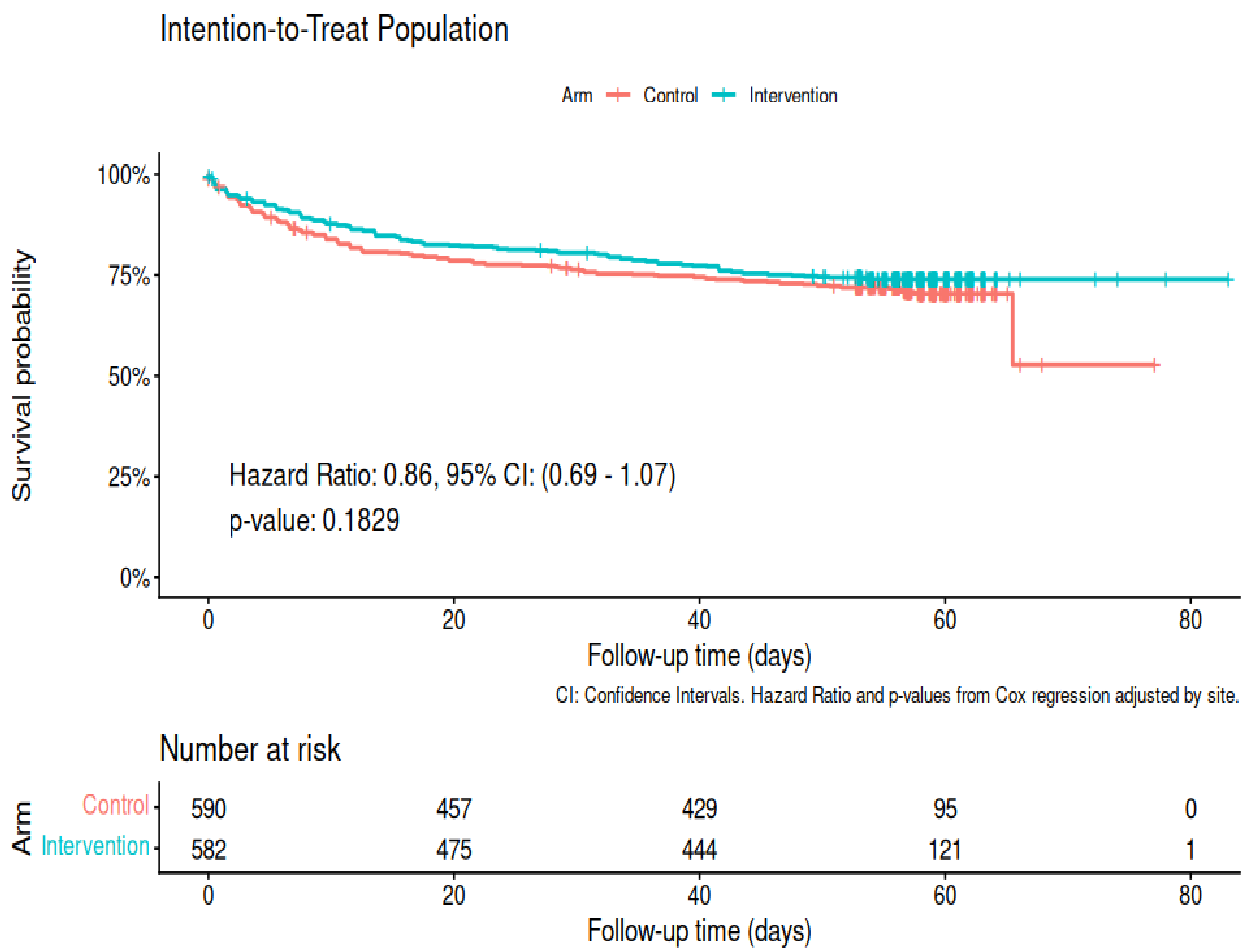

	Control (N=420)	Intervention (N=432)	
**Censoring outcome**	N=420	N=432	0.3220[a]
Administrative censoring	408 (97.1%)	425 (98.4%)	
Loss to follow-up	12 (2.9%)	7 (1.6%)	

[a]: Chi-square test; [b]: Fisher exact test; [c]: t-test; [d]: Mann-Whitney U test.

Figure S6. Per-Protocol-Population

Group	Numerator	Denominator	Proportion	Risk Difference	95% Confidence interval	p value
Control	84	307	27.4	-3.3%	(-10.9% to 4.3%)	0.3920
Intervention	53	222	23.9			

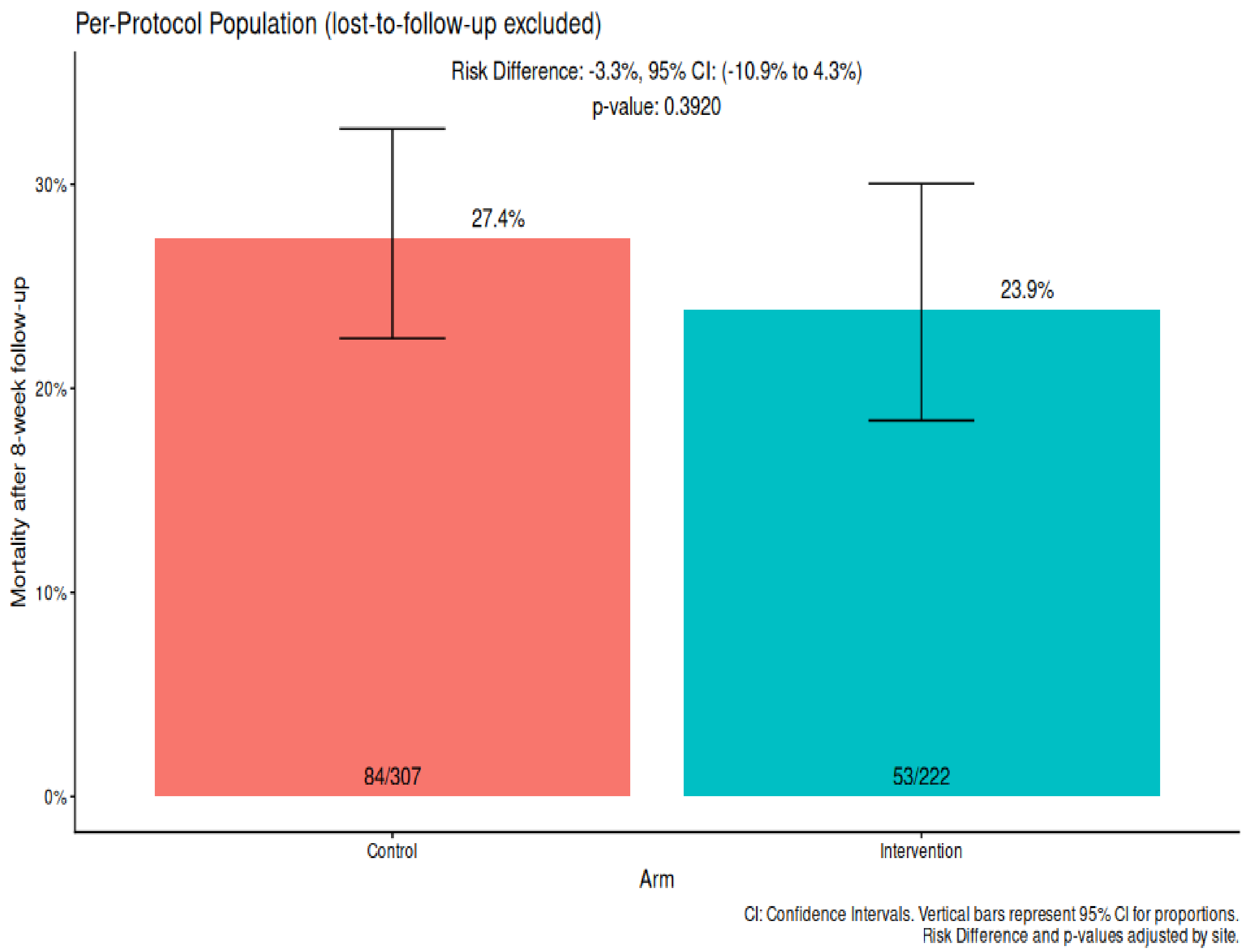

Figure S7. Survival curves in Per-Protocol-Population

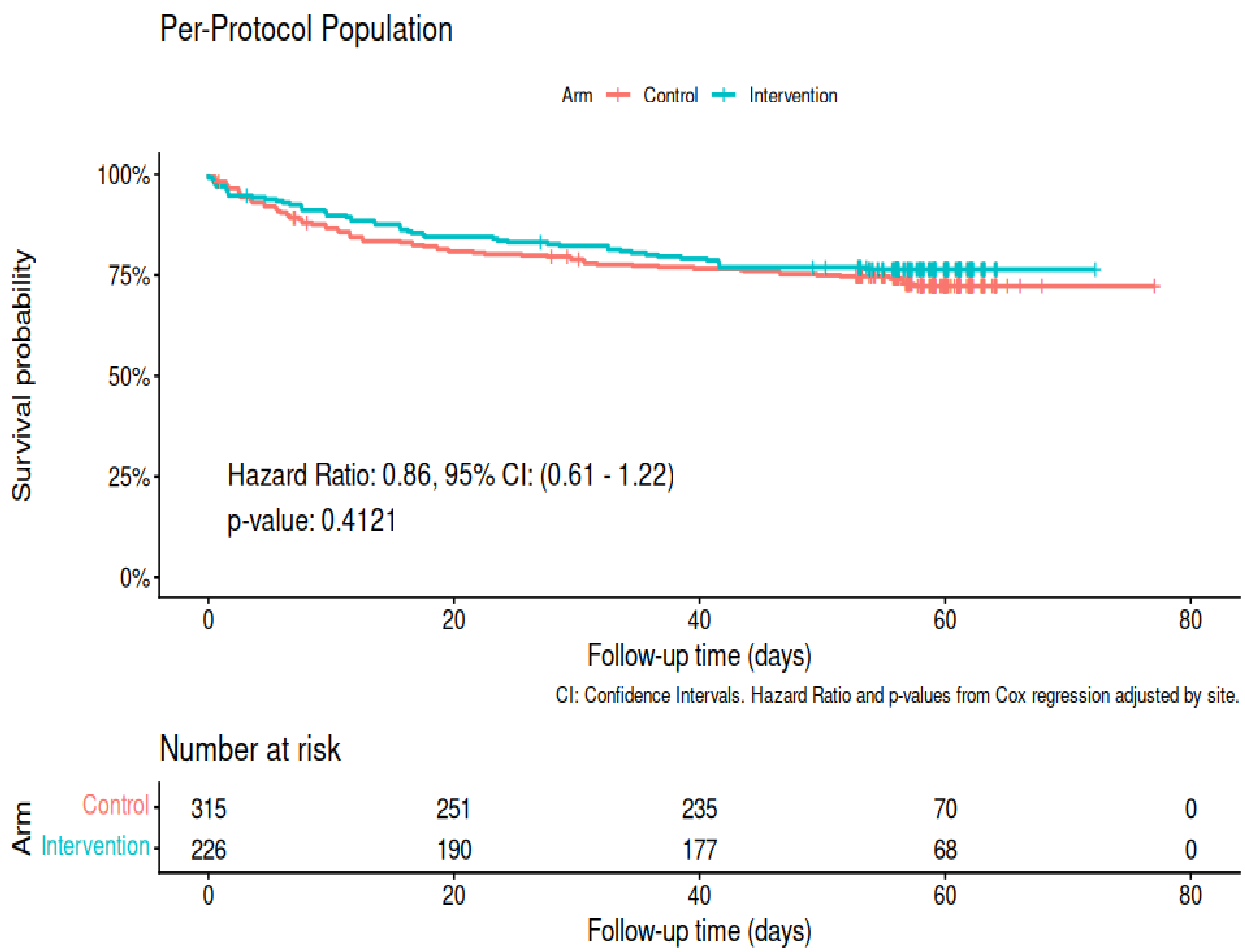

Secondary Endpoint: Time to treatment initiation

Figure S8. Intention-to-Treat Population

	Control (N=590)	Intervention (N=582)	p-value
**Time to TB treatment (days)**	N=130	N=135	0.3809[d]
Mean (SD)	1.74 (2.34)	2.47 (5.16)	
Median [Q1, Q3]	0.922 [0.792, 1.86]	0.982 [0.831, 1.92]	

[a]: Chi-square test; [b]: Fisher exact test; [c]: t-test; [d]: Mann-Whitney U test.

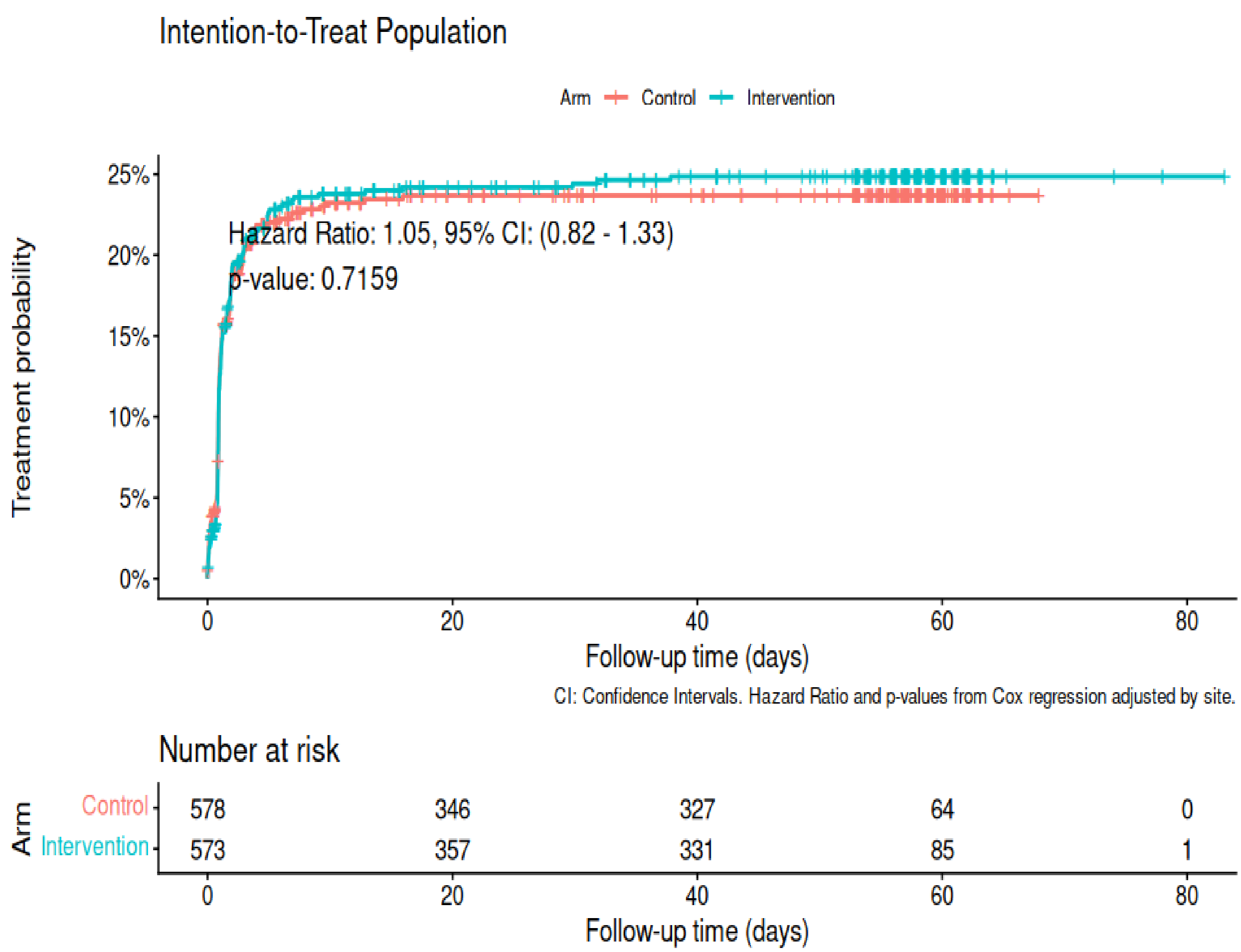

	Control (N=448)	Intervention (N=438)	
**Censoring outcome**	N=448	N=438	0.5422[a]
Administrative censoring	312 (69.6%)	319 (72.8%)	
Competing event (death)	129 (28.8%)	114 (26.0%)	
Loss to follow-up	7 (1.6%)	5 (1.1%)	

[a]: Chi-square test; [b]: Fisher exact test; [c]: t-test; [d]: Mann-Whitney U test.

Figure S9. Per-Protocol-Population

	Control (N=315)	Intervention (N=226)	p-value
**Time to TB treatment (days)**	N=93	N=69	
Mean (SD)	1.73 (2.42)	2.18 (4.05)	0.3896[d]
Median [Q1, Q3]	0.906 [0.809, 1.83]	0.972 [0.833, 1.90]	

[a]: Chi-square test; [b]: Fisher exact test; [c]: t-test; [d]: Mann-Whitney U test.

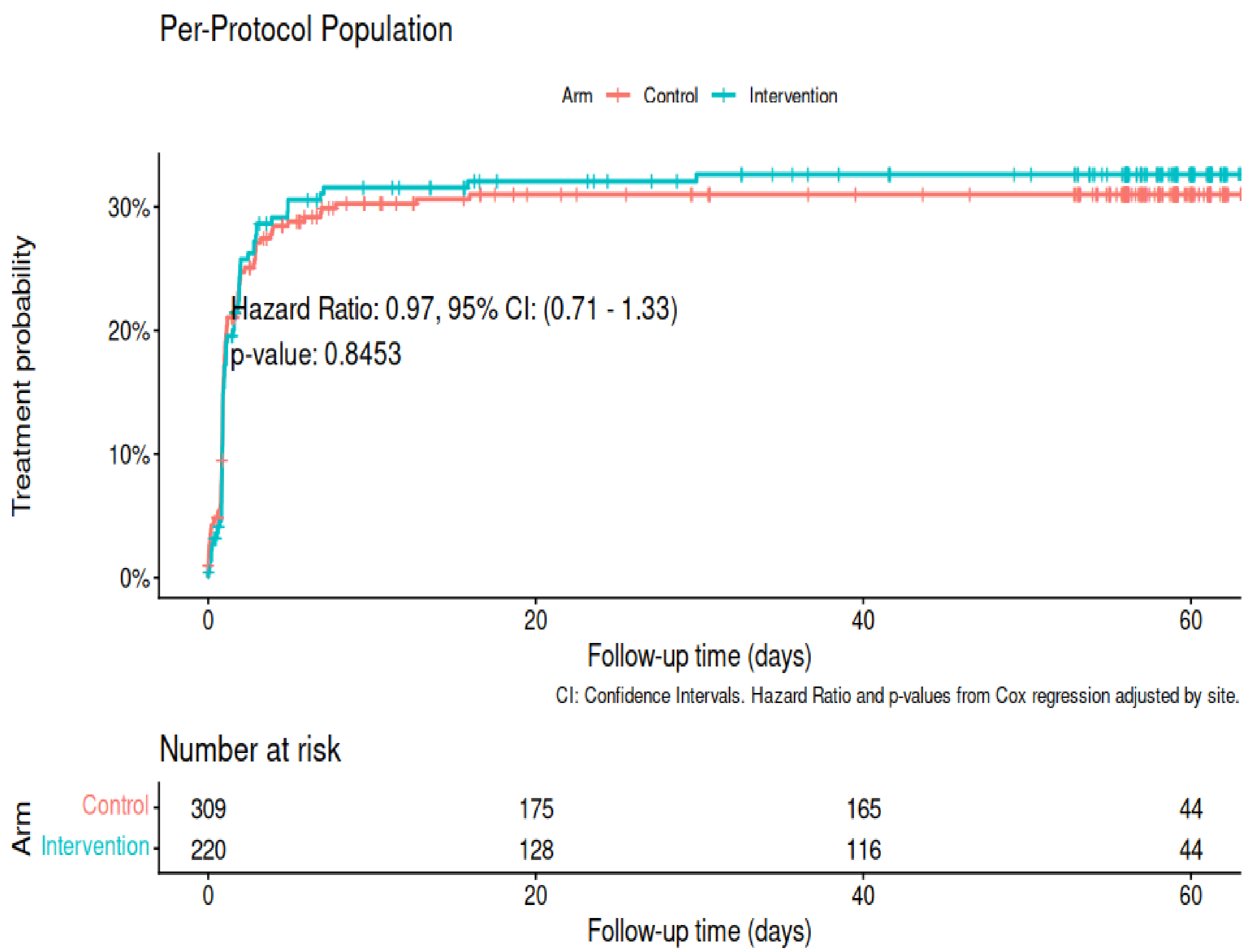

Secondary Endpoint: TB diagnosis

Figure S10. Intention-to-Treat Population

Group	Numerator	Denominator	Proportion	Risk Difference	95% Confidence interval	p value
Control	190	590	32.2	1.2%	(-4.1% to 6.5%)	0.6488
Intervention	195	582	33.5			

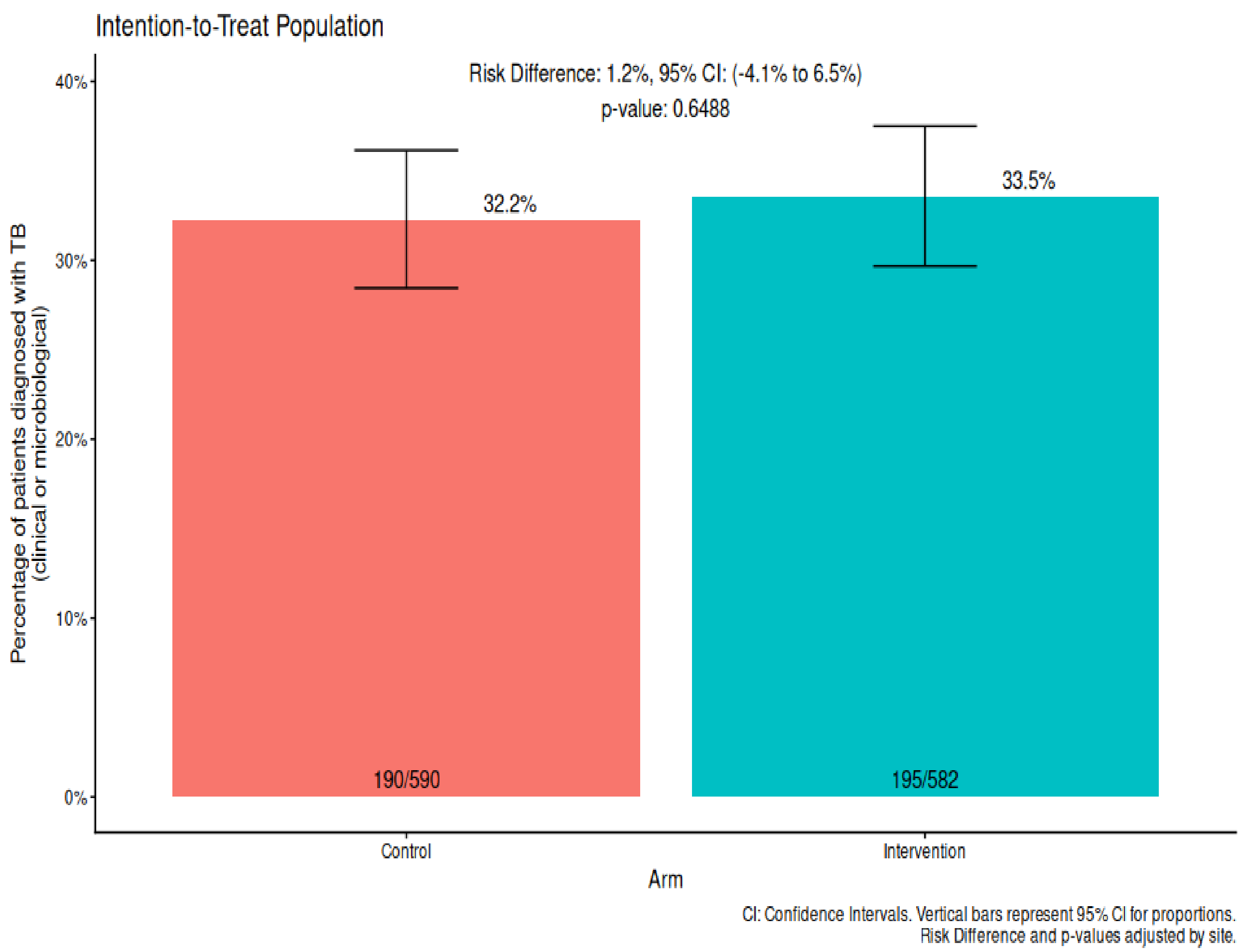

Figure S11. Per-Protocol Population

Group	Numerator	Denominator	Proportion	Risk Difference	95% Confidence interval	p value
Control	122	315	38.7	4.4%	(-4.0% to 12.8%)	0.3086
Intervention	99	226	43.8			

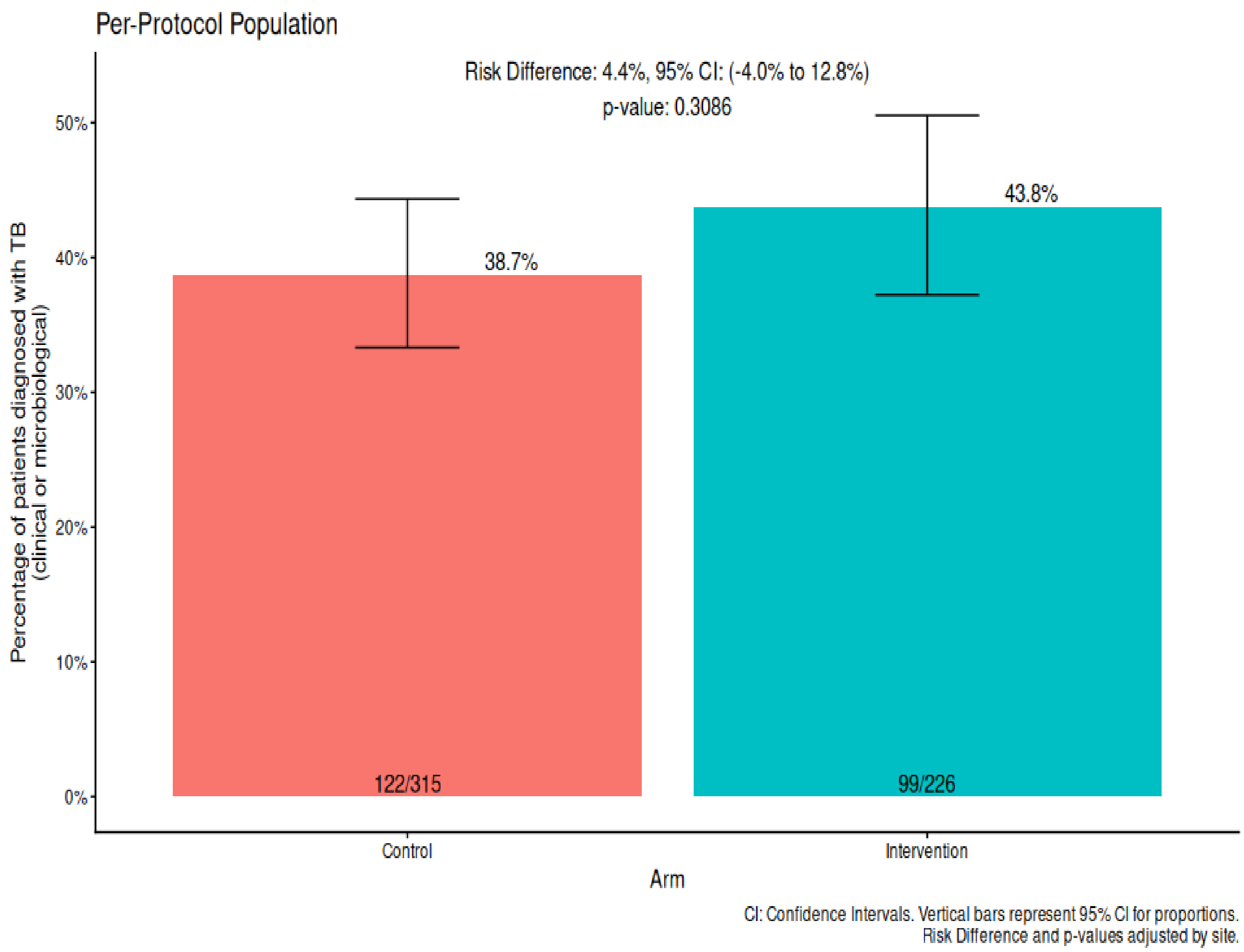

Secondary Endpoint: Diagnosis and treatment day 7, 14, week 4, 8

Proportion of participants who are diagnosed with microbiologically confirmed TB and are started on treatment at 7 days, 14 days, 4 weeks, and 8 weeks.

Microbiological Diagnosis and Treatment at Day 7

Figure S12. Intention to treat population

Group	Numerator	Denominator	Proportion	Risk Difference	95% Confidence interval	p value
Control	95	590	16.1	0.4%	95% Confidence interval	p-value
Intervention	96	582	16.5		(-3.8% to 4.6%)	0.8512

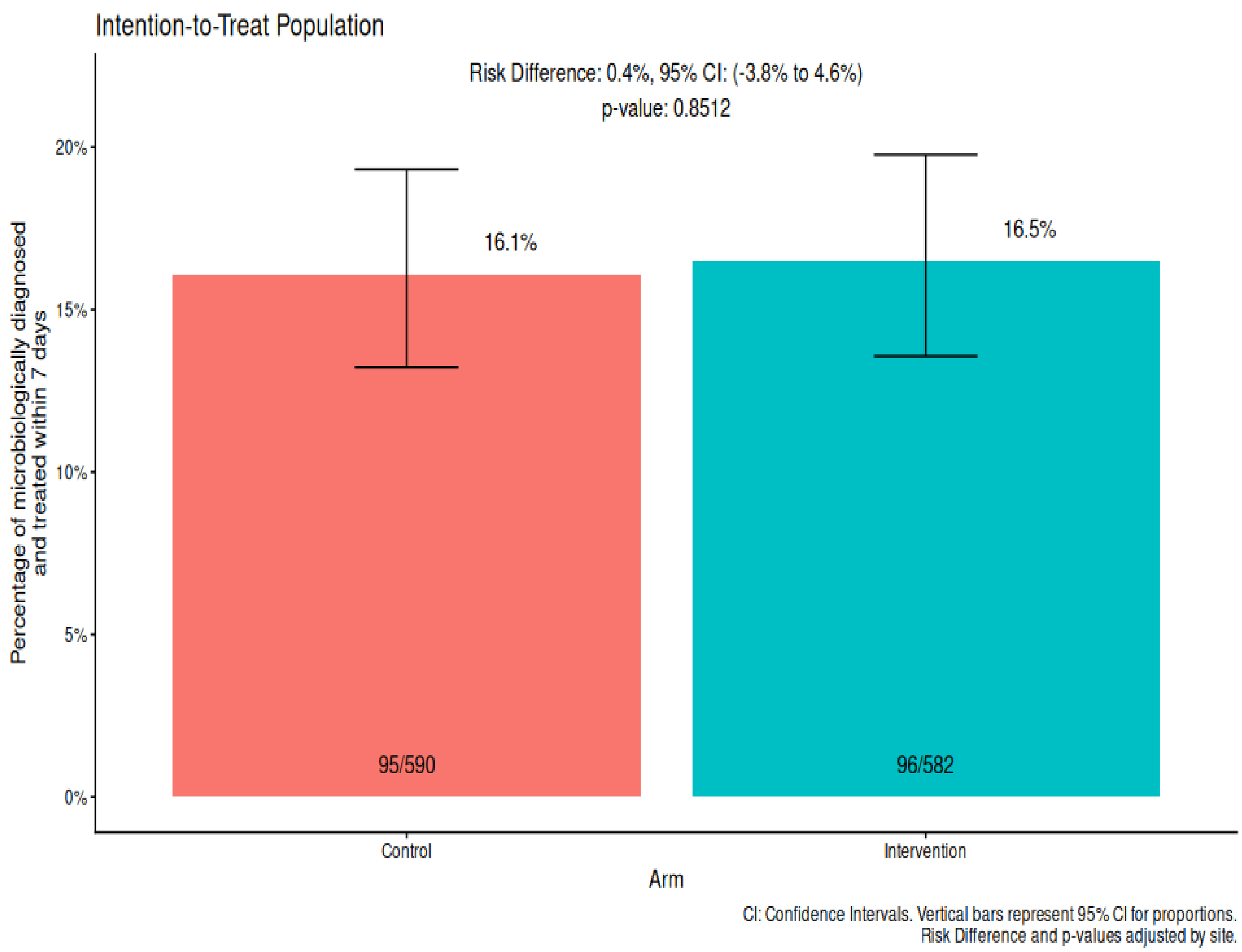

Intention-to-Treat Population
Risk Difference: 0.4%, 95% CI: (-3.8% to 4.6%)
p-value: 0.8512
Percentage of microbiologically diagnosed and treated within 7 days
20%
15%
10%
5%
0%
16.1%
16.5%
95/590
96/582
Control
Intervention
Arm
CI: Confidence Intervals. Vertical bars represent 95% CI for proportions.
Risk Difference and p-values adjusted by site.

Figure S13. Per protocol population

Group	Numerator	Denominator	Proportion	Risk Difference	95% Confidence interval	p value
Control	71	315	22.5	-3.9%	(-10.8% to 3.0%)	0.2724
Intervention	44	226	19.5			

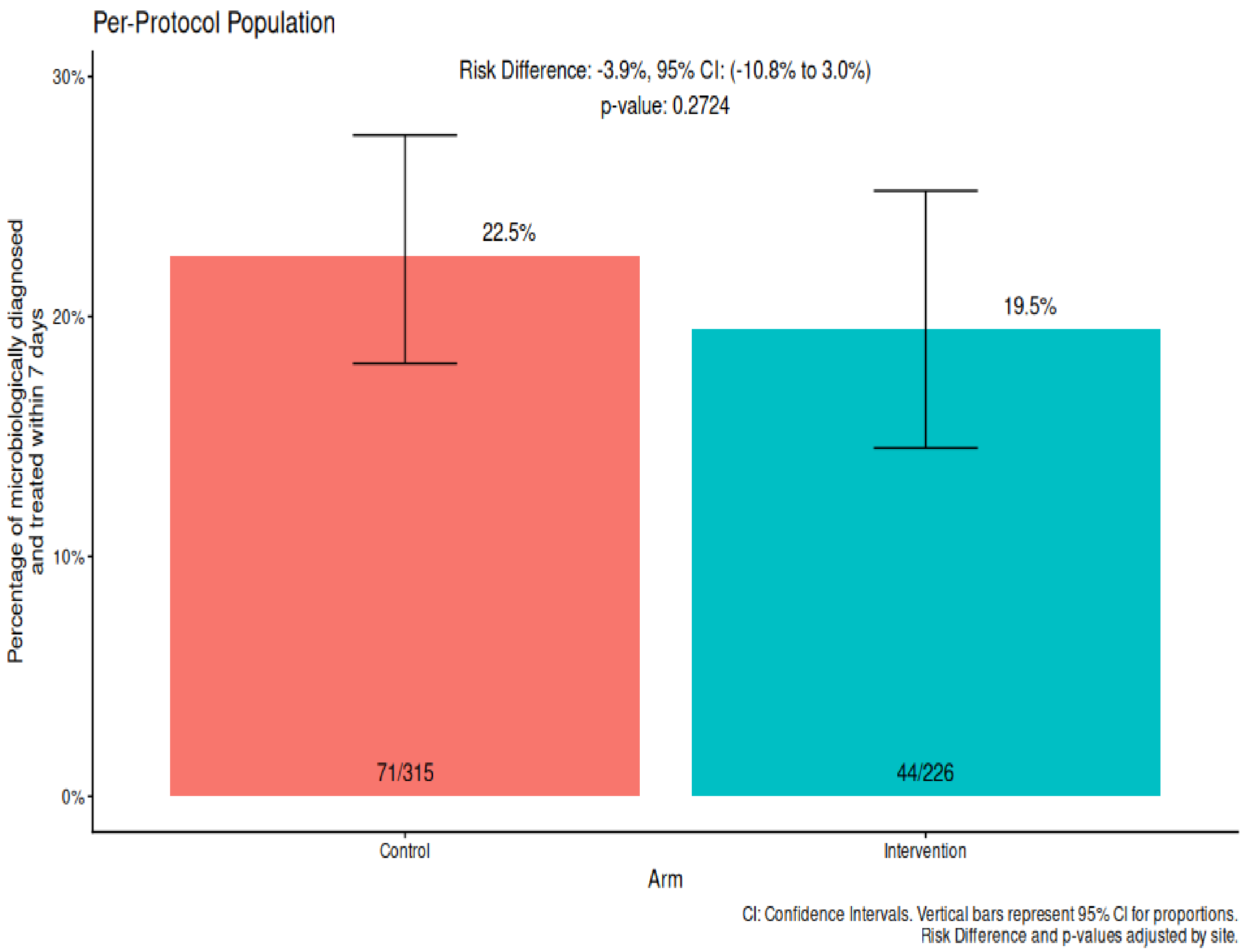

Microbiological Diagnosis and Treatment at Day 14

Figure S14. Intention to treat population

Group	Numerator	Denominator	Proportion	Risk Difference	95% Confidence interval	p value
Control	97	590	16.4	0.2%	(-4.0% to 4.4%)	0.9145
Intervention	97	582	16.7			

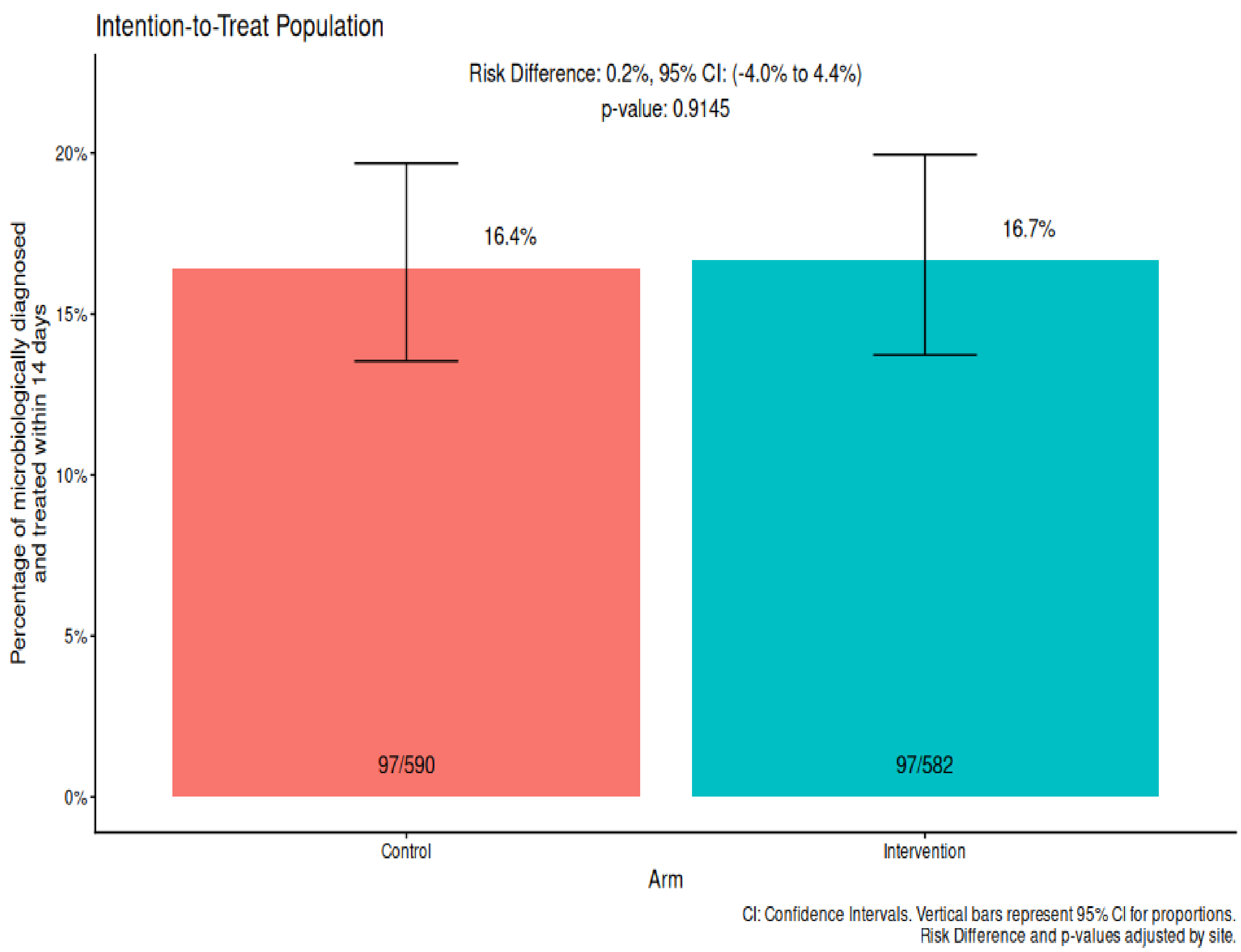

Figure S15. Per Protocol population

Group	Numerator	Denominator	Proportion	Risk Difference	95% Confidence interval	p value
Control	71	315	22.5	-3.9%	(-10.8% to 3.0%)	0.2724
Intervention	44	226	19.5			

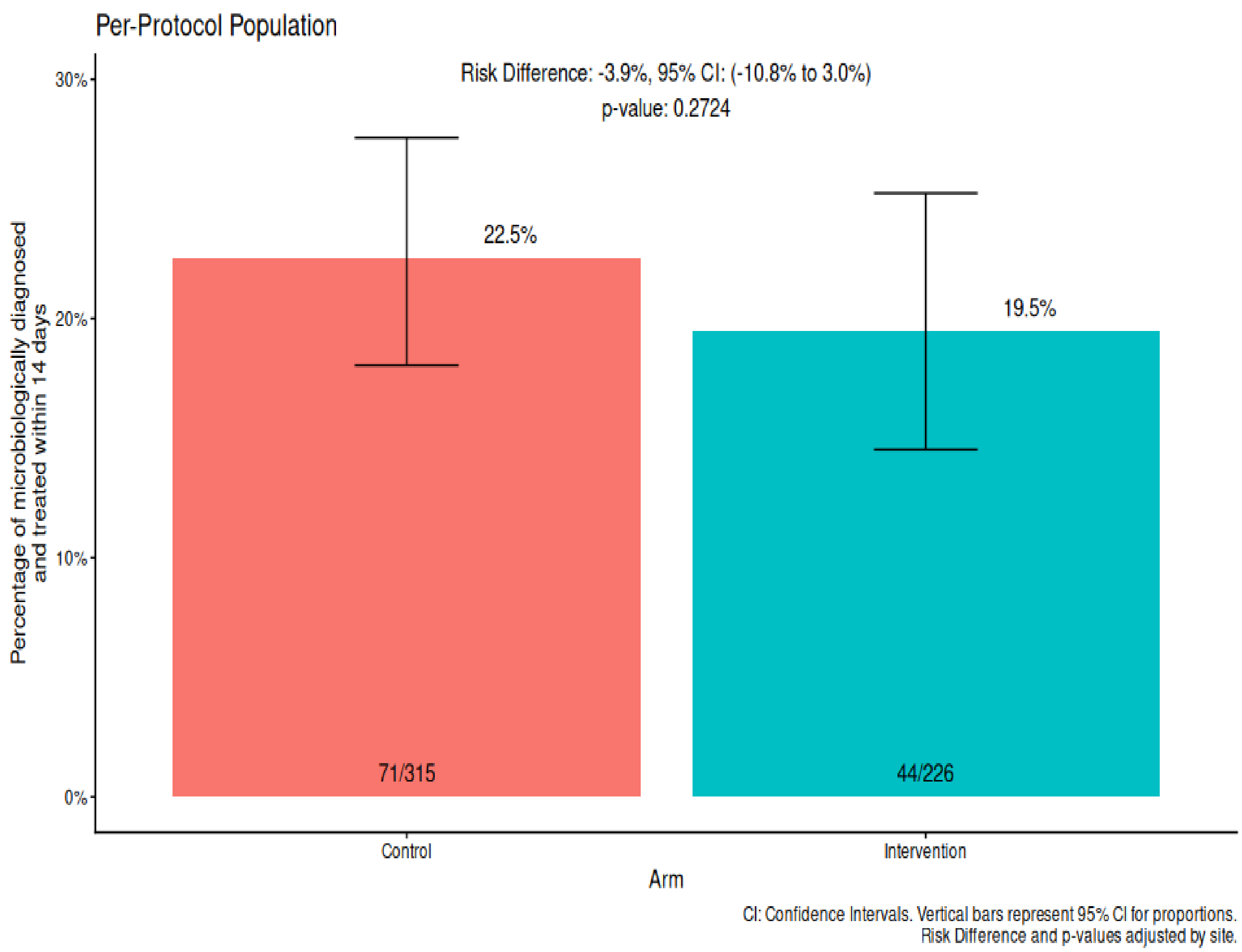

Microbiological Diagnosis and Treatment at Week 4

Figure S16. Intention to treat population

Group	Numerator	Denominator	Proportion	Risk Difference	95% Confidence interval	p value
Control	97	590	16.4	0.4%	(-3.8% to 4.6%)	0.8532
Intervention	98	582	16.8			

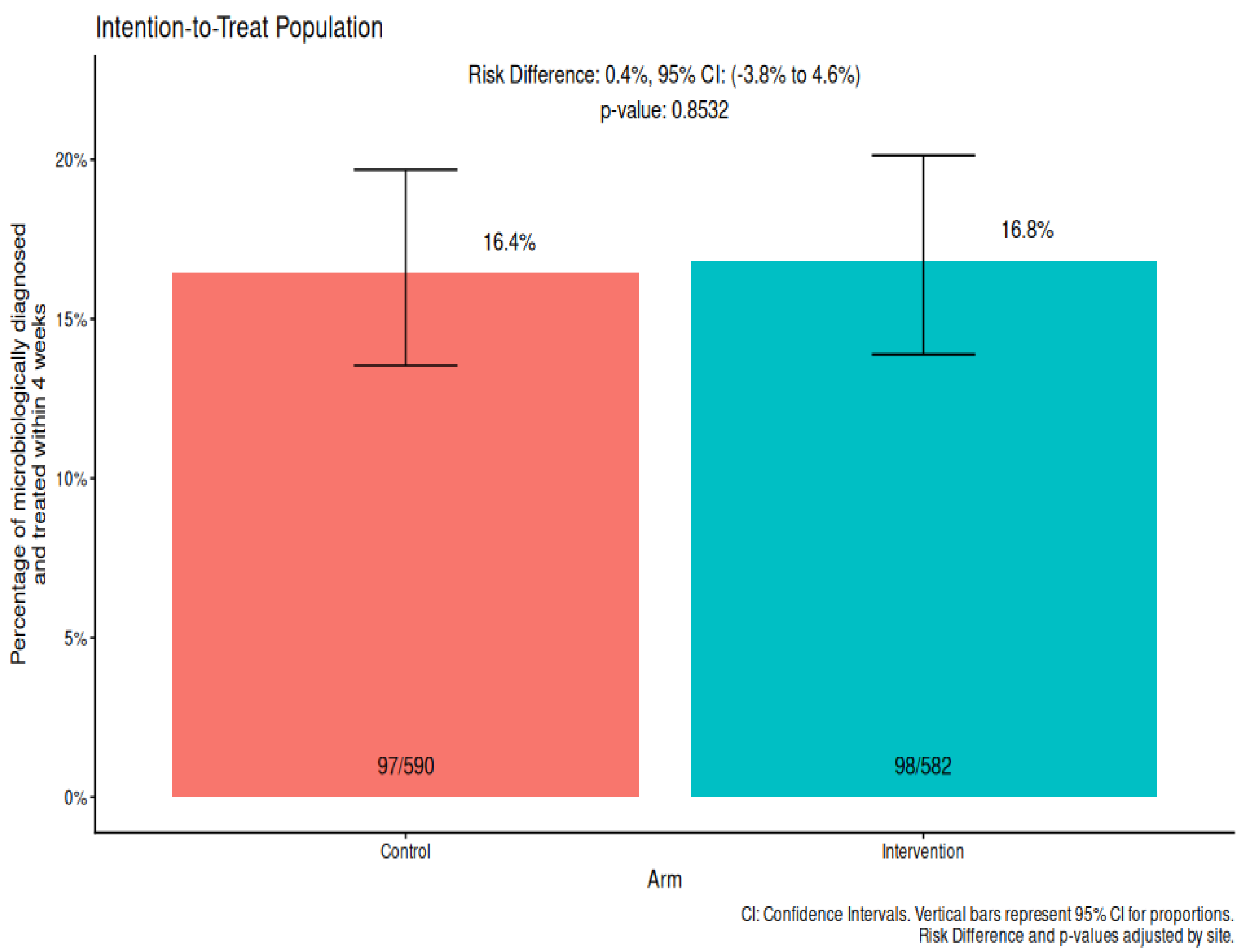

Figure S17. Per-protocol population

Group	Numerator	Denominator	Proportion	Risk Difference	95% Confidence interval	p value
Control	71	315	22.5	-3.5%	(-10.4% to 3.5%)	0.3294
Intervention	45	226	19.9			

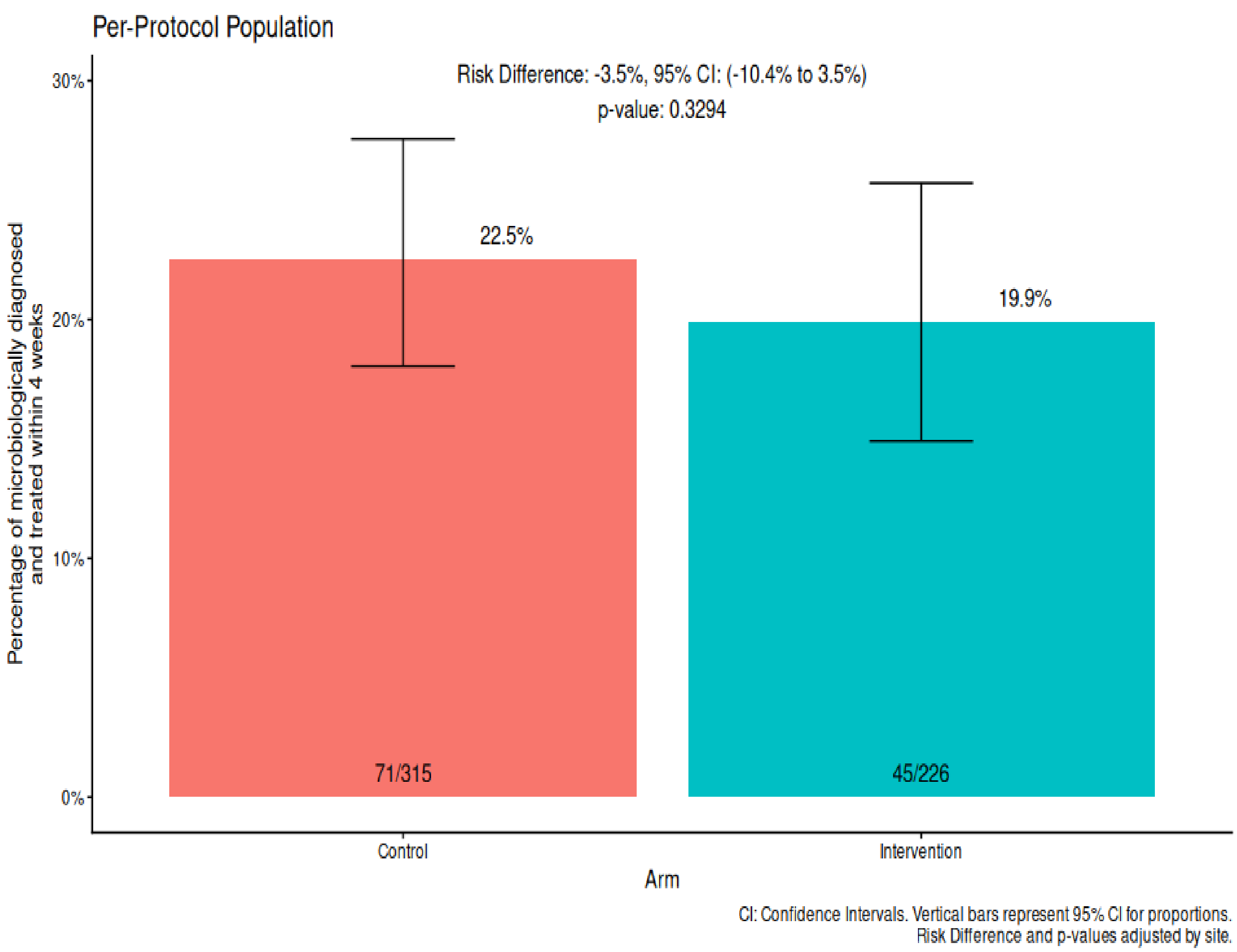

Microbiological Diagnosis and Treatment at Week 8

Figure S18. Intention to treat population

Group	Numerator	Denominator	Proportion	Risk Difference	95% Confidence interval	p value
Control	97	590	16.4	0.7%	(-3.5% to 5.0%)	0.7341
Intervention	100	582	17.2			

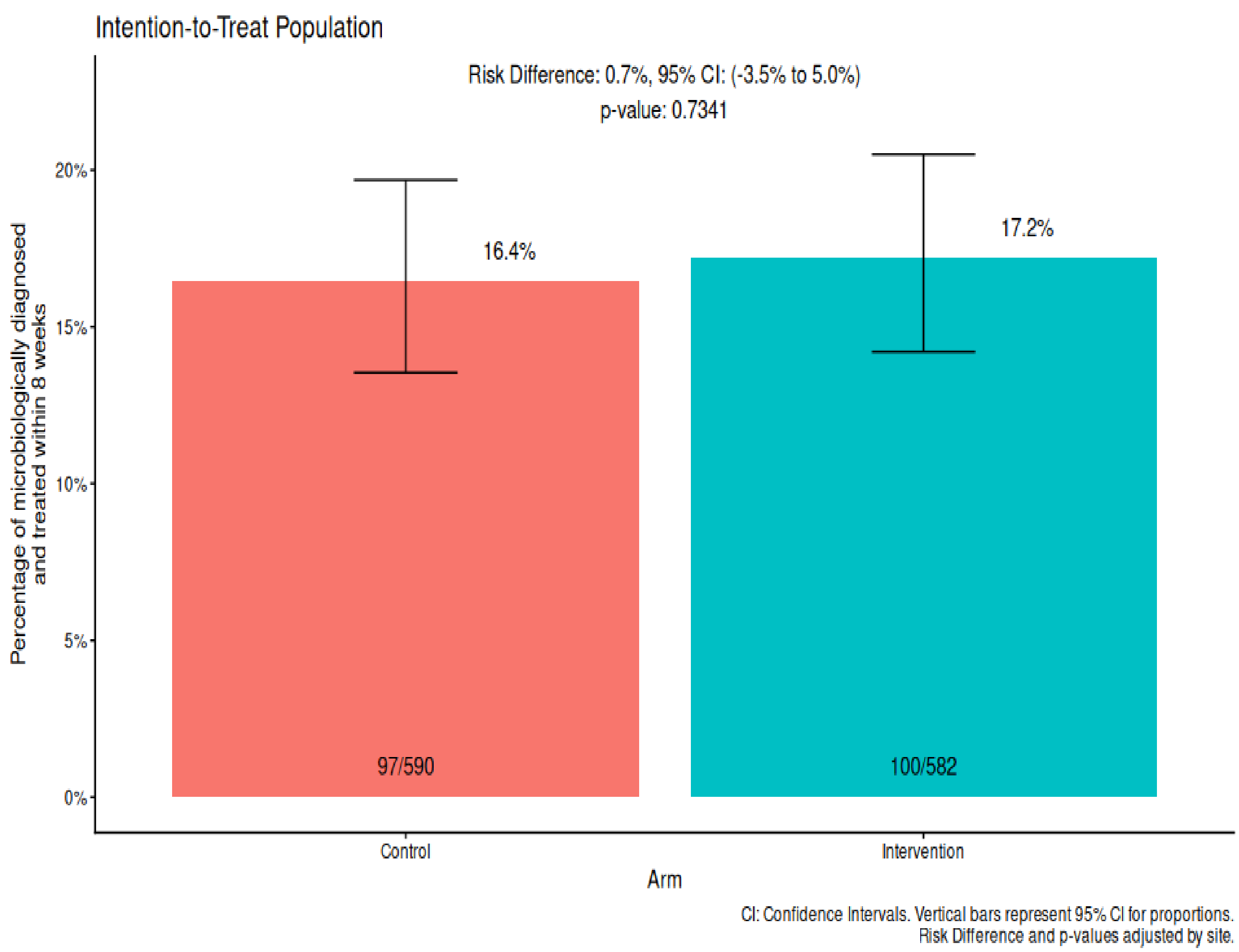

Figure S19. Per-protocol population

Group	Numerator	Denominator	Proportion	Risk Difference	95% Confidence interval	p value
Control	71	315	22.5	-3.0%	(-10.0% to 3.9%)	0.3929
Intervention	46	226	20.4			

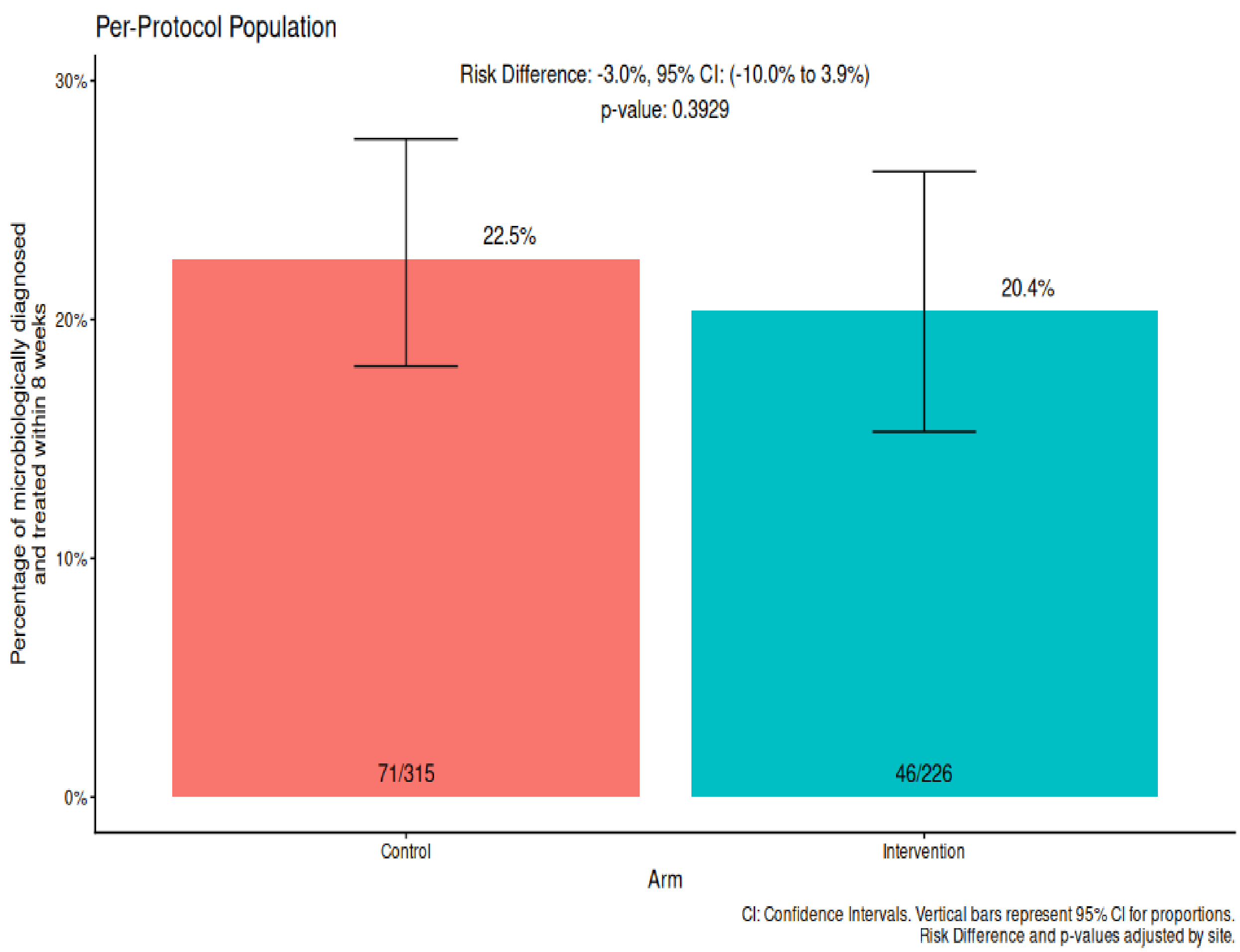

Secondary Endpoint: Proportion of participants able to provide samples at 24 hours, 48 hours, and 72 or more hours (table S11)

	Control (N=590)	Intervention (N=582)	p-value
**Sputum sample available 24 hours from enrolment**	N=590	N=582	
No	318 (53.9%)	282 (48.5%)	0.0709[a]
Yes	272 (46.1%)	300 (51.5%)	
**Sputum sample available 48 hours from enrolment**	N=590	N=582	
No	296 (50.2%)	268 (46.0%)	0.1759[a]
Yes	294 (49.8%)	314 (54.0%)	
**Sputum sample available 72 or more hours from enrolment**	N=590	N=582	
No	286 (48.5%)	265 (45.5%)	0.3419[a]
Yes	304 (51.5%)	317 (54.5%)	
**Stool sample available 24 hours from enrolment**	N=590	N=582	
No	586 (99.3%)	357 (61.3%)	<0.0001[a]
Yes	4 (0.7%)	225 (38.7%)	
**Stool sample available 48 hours from enrolment**	N=590	N=582	
No	586 (99.3%)	314 (54.0%)	<0.0001[a]

	Control (N=590)	Intervention (N=582)	p-value
Yes	4 (0.7%)	268 (46.0%)	
**Stool sample available 72 or more hours from enrolment**	N=590	N=582	
No	586 (99.3%)	304 (52.2%)	<0.0001[a]
Yes	4 (0.7%)	278 (47.8%)	
**Urine sample available 24 hours from enrolment**	N=590	N=582	
No	102 (17.3%)	63 (10.8%)	0.0020[a]
Yes	488 (82.7%)	519 (89.2%)	
**Urine sample available 48 hours from enrolment**	N=590	N=582	
No	70 (11.9%)	24 (4.1%)	<0.0001[a]
Yes	520 (88.1%)	558 (95.9%)	
**Urine sample available 72 or more hours from enrolment**	N=590	N=582	
No	62 (10.5%)	18 (3.1%)	<0.0001[a]
Yes	528 (89.5%)	564 (96.9%)	
**Rectal swab sample available 24 hours from enrolment**	N=590	N=582	
No	590 (100%)	411 (70.6%)	<0.0001[a]
Yes	0 (0%)	171 (29.4%)	

	Control (N=590)	Intervention (N=582)	p-value
**Rectal swab sample available 48 hours from enrolment**	N=590	N=582	
No	590 (100%)	402 (69.1%)	<0.0001[a]
Yes	0 (0%)	180 (30.9%)	
**Rectal swab sample available 72 or more hours from enrolment**	N=590	N=582	
No	590 (100%)	401 (68.9%)	<0.0001[a]
Yes	0 (0%)	181 (31.1%)	

[a]: Chi-square test; [b]: Fisher exact test; [c]: t-test; [d]: Mann-Whitney U test.

Secondary Endpoint: Time to microbiologically confirmed TB diagnosis

Table S12. Intention-to-Treat Population

	Control (N=590)	Intervention (N=582)	p-value
**Time to first test positivity (hours)**	N=147	N=149	0.1809 [d]
Mean (SD)	264 (1270)	10.2 (16.5)	
Median [Q1, Q3]	1.43 [0.842, 4.98]	2.15 [0.900, 19.4]	
Missing	443 (75.1%)	433 (74.4%)	
**Time to LF-LAM result**	N=541	N=558	<0.0001 [a]
First result in >72 hours	36 (6.7%)	5 (0.9%)	
First result in ≤72 hours	505 (93.3%)	553 (99.1%)	
Missing	49 (8.3%)	24 (4.1%)	
**Time to sputum Ultra result**	N=296	N=314	0.0006 [a]
First result in >72 hours	31 (10.5%)	10 (3.2%)	
First result in ≤72 hours	265 (89.5%)	304 (96.8%)	
Missing	294 (49.8%)	268 (46.0%)	
**Time to stool Ultra result**	N=1	N=266	1.0000 [b]
First result in >72 hours	0 (0%)	17 (6.4%)	
First result in ≤72 hours	1 (100%)	249 (93.6%)	
Missing	589 (99.8%)	316 (54.3%)	
**Time to urine Ultra result**	N=12	N=541	0.2545 [b]
First result in >72 hours	2 (16.7%)	43 (7.9%)	
First result in ≤72 hours	10 (83.3%)	498 (92.1%)	

	Control (N=590)	Intervention (N=582)	p-value
Missing	578 (98.0%)	41 (7.0%)	
**Time to other specimen Ultra result**	N=34	N=189	0.7170 b
First result in >72 hours	3 (8.8%)	13 (6.9%)	
First result in ≤72 hours	31 (91.2%)	176 (93.1%)	
Missing	556 (94.2%)	393 (67.5%)	
**Time to other specimen (2) Ultra result**	N=0	N=9	1.0000 b
First result in >72 hours	0 (NA%)	3 (33.3%)	
First result in ≤72 hours	0 (NA%)	6 (66.7%)	
Missing	590 (100%)	573 (98.5%)	

[a]: Chi-square test; [b]: Fisher exact test; [c]: t-test; [d]: Mann-Whitney U test.

Table S13. Per-Protocol-Population

	Control (N=315)	Intervention (N=226)	p-value
**Time to first test positivity (hours)**	N=96	N=69	
Mean (SD)	5.07 (8.97)	6.29 (9.70)	0.3372 d
Median [Q1, Q3]	1.24 [0.750, 3.10]	1.40 [0.833, 5.55]	
Missing	219 (69.5%)	157 (69.5%)	
**Time to LF-LAM result**	N=283	N=226	
First result in >72 hours	0 (0%)	0 (0%)	1.0000 b
First result in ≤72 hours	283 (100%)	226 (100%)	
Missing	32 (10.2%)	0 (0%)	
**Time to sputum Ultra result**	N=254	N=221	
First result in >72 hours	0 (0%)	0 (0%)	1.0000 b
First result in ≤72 hours	254 (100%)	221 (100%)	
Missing	61 (19.4%)	5 (2.2%)	
**Time to stool Ultra result**	N=0	N=123	
First result in >72 hours	0 (NA%)	0 (0%)	1.0000 b
First result in ≤72 hours	0 (NA%)	123 (100%)	
Missing	315 (100%)	103 (45.6%)	
**Time to urine Ultra result**	N=0	N=226	
First result in >72 hours	0 (NA%)	0 (0%)	1.0000 b
First result in ≤72 hours	0 (NA%)	226 (100%)	
Missing	315 (100%)	0 (0%)	
**Time to other specimen Ultra result**	N=8	N=102	
First result in >72 hours	0 (0%)	0 (0%)	1.0000 b

	Control (N=315)	Intervention (N=226)	p-value
First result in ≤72 hours	8 (100%)	102 (100%)	
Missing	307 (97.5%)	124 (54.9%)	
**Time to other specimen (2) Ultra result**	N=0	N=2	
First result in >72 hours	0 (NA%)	0 (0%)	1.0000 [b]
First result in ≤72 hours	0 (NA%)	2 (100%)	
Missing	315 (100%)	224 (99.1%)	

[a]: Chi-square test; [b]: Fisher exact test; [c]: t-test; [d]: Mann-Whitney U test.

Figure S20. Time-to-diagnosis, survival analysis. Intention to treat population

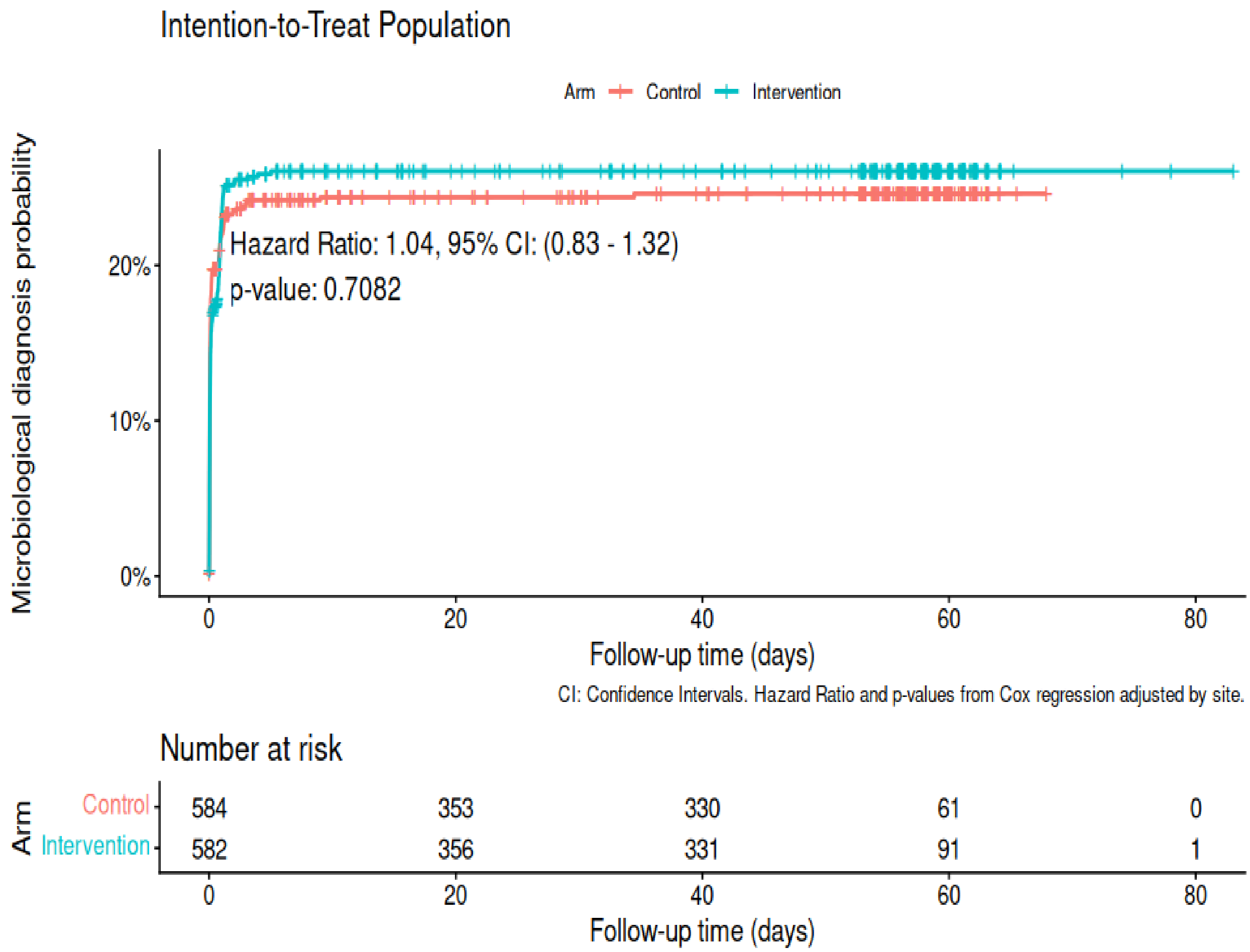

	Control (N=443)	Intervention (N=433)	
**Censoring outcome**	N=443	N=433	0.3629[a]
Administrative censoring	313 (70.7%)	318 (73.4%)	
Competing event (death)	120 (27.1%)	110 (25.4%)	
Loss to follow-up	10 (2.3%)	5 (1.2%)	

[a]: Chi-square test; [b]: Fisher exact test; [c]: t-test; [d]: Mann-Whitney U test.

Figure S21. Time-to-diagnosis, survival analysis. Per-protocol population

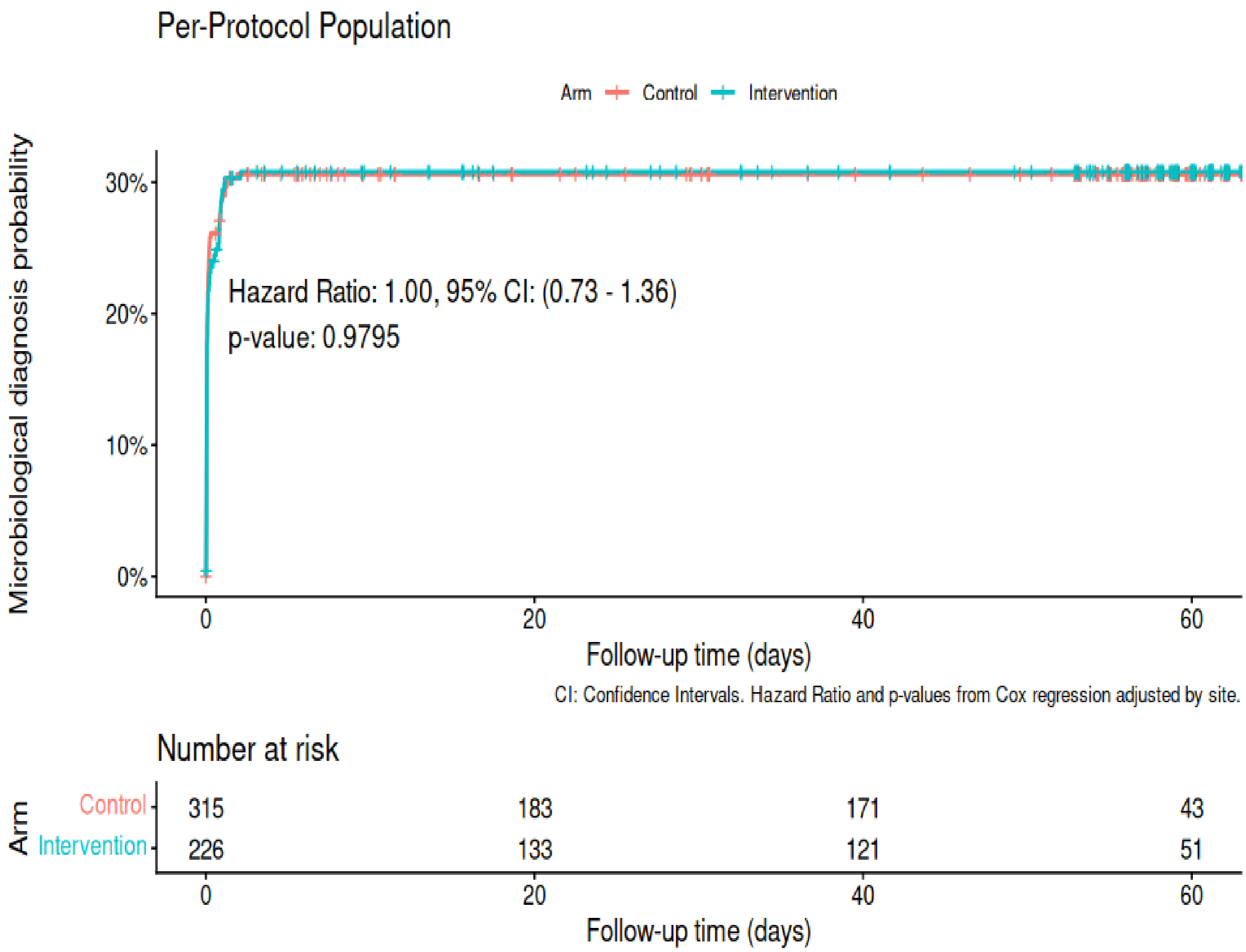

Secondary Endpoint: 4-week all-cause mortality

Figure S22. Intention-to-Treat Population

Group	Numerator	Denominator	Proportion	Risk Difference	95% Confidence interval	p value
Control	133	590	22.5	-3.6%	(-8.2% to 1.0%)	0.1263
Intervention	110	582	18.9			

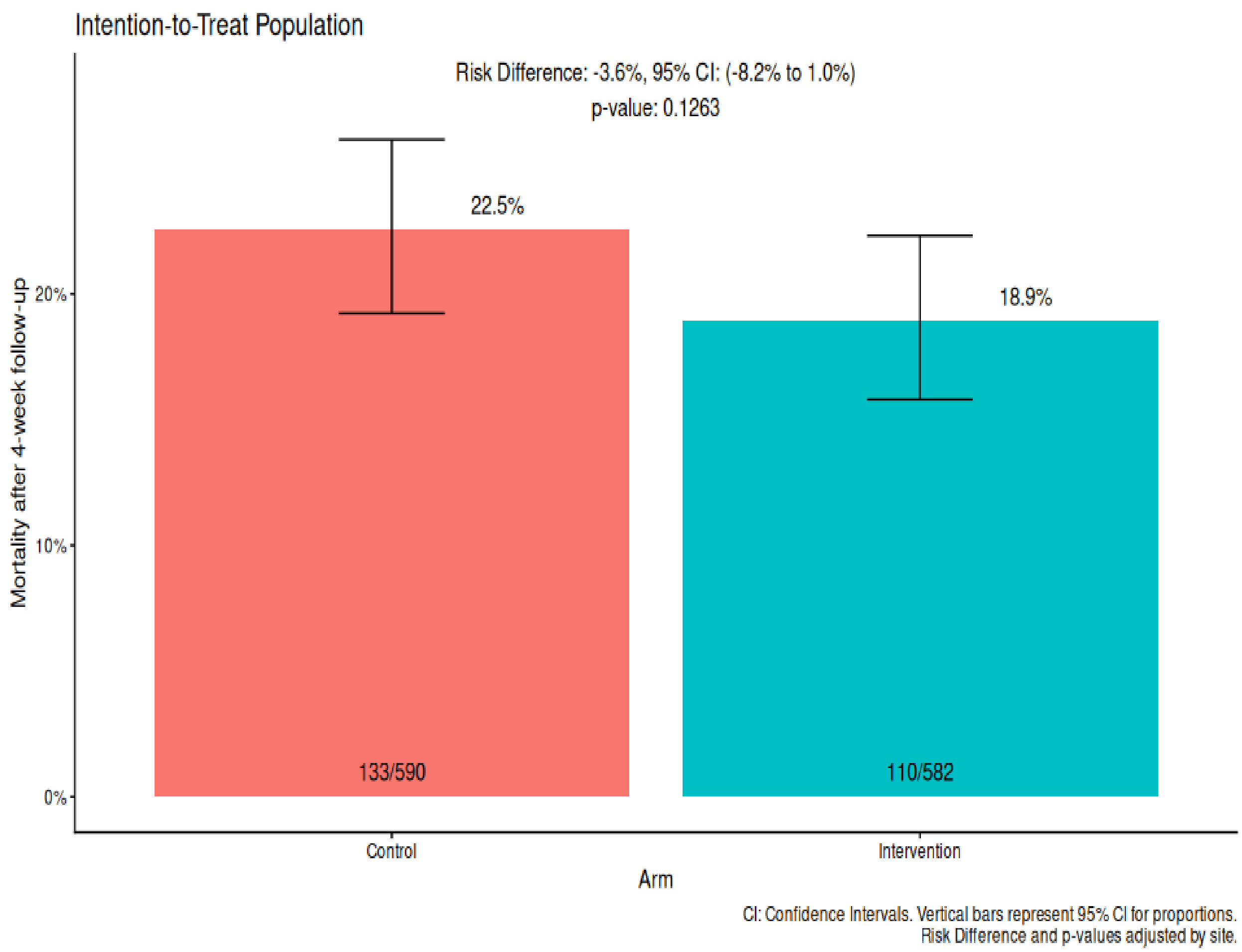

Figure S23. Per-Protocol-Population

Group	Numerator	Denominator	Proportion	Risk Difference	95% Confidence interval	p value
Control	64	307	20.8	-3.2%	(-10.0% to 3.7%)	0.3649
Intervention	39	222	17.6			

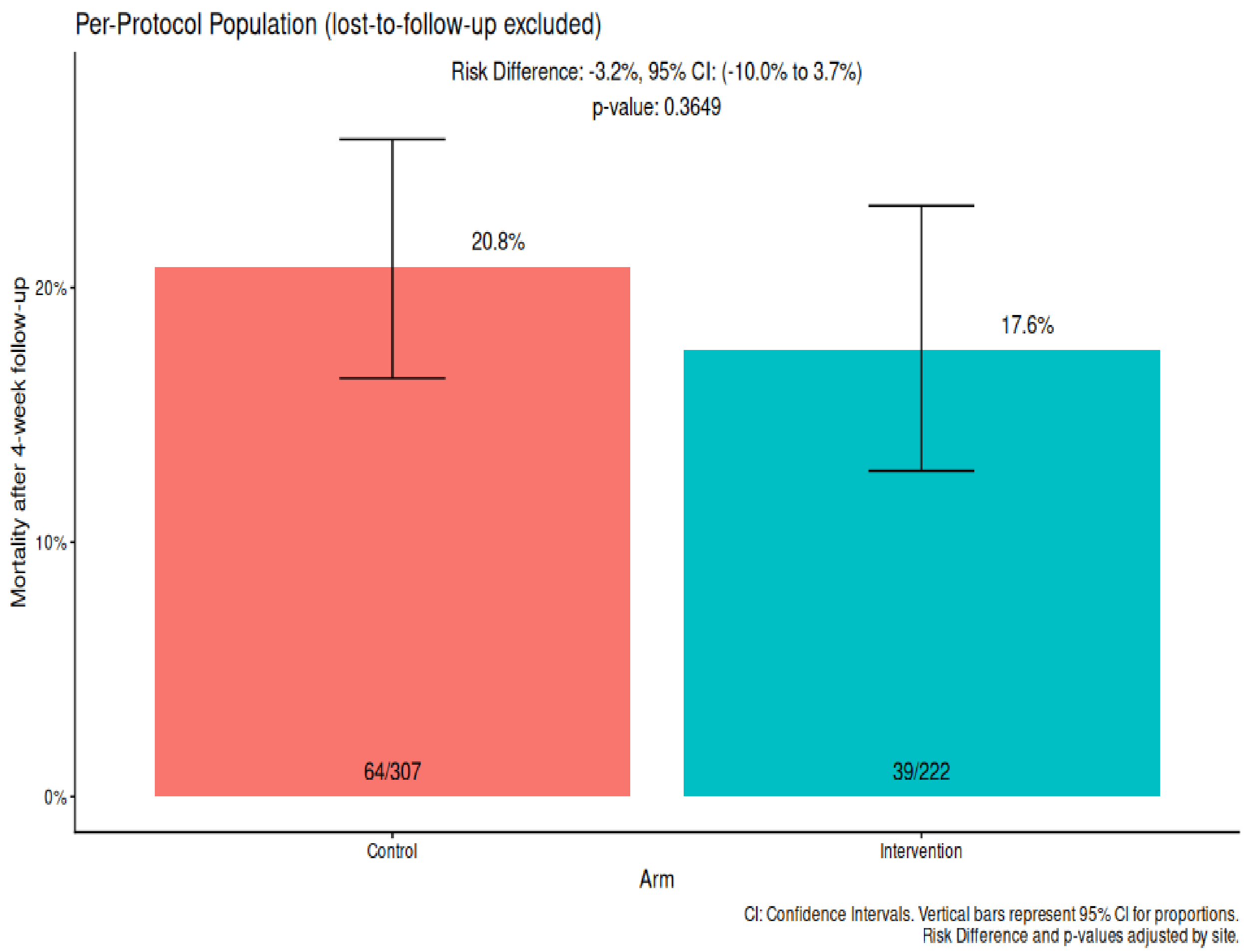

Secondary Endpoint: proportion of participants started on treatment without Rif resistance information (only LF-LAM positive)

Table S14. Intention-to-Treat Population

	Control (N=142)	Intervention (N=144)	p-value
**Rifampicine resistance information available**	N=142	N=144	
No	107 (75.4%)	94 (65.3%)	0.0828[a]
Yes	35 (24.6%)	50 (34.7%)	
**Rifampicine resistance**	N=35	N=50	
No	34 (97.1%)	46 (92.0%)	0.6445[b]
Yes	1 (2.9%)	4 (8.0%)	
Missing	107 (75.4%)	94 (65.3%)	

[a]: Chi-square test; [b]: Fisher exact test; [c]: t-test; [d]: Mann-Whitney U test.

Figure S24. Intention-to-Treat Population

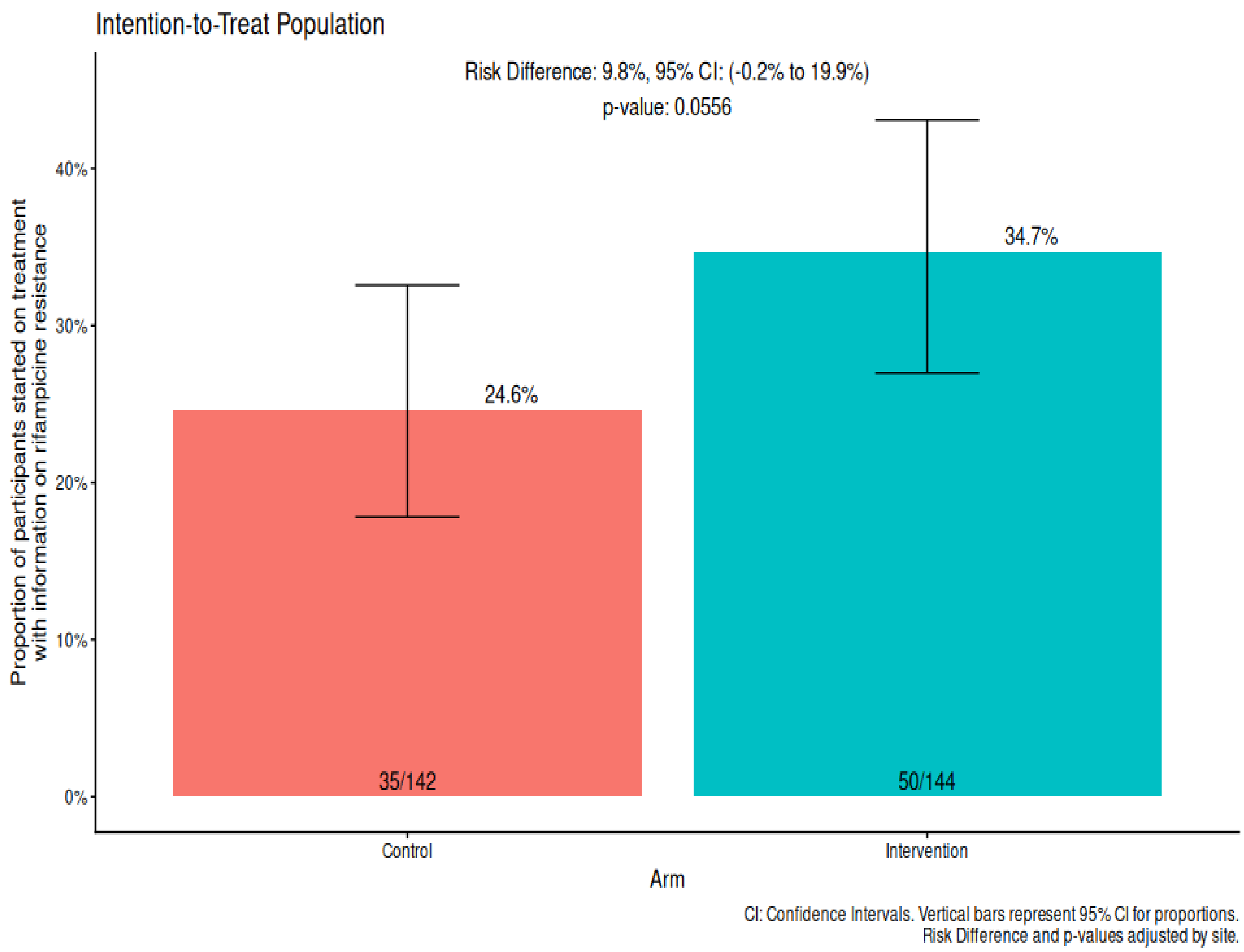

Table S15. Per-Protocol Population

	Control (N=99)	Intervention (N=75)	p-value
**Rifampicine resistance information available**	N=99	N=75	
No	70 (70.7%)	52 (69.3%)	0.9770[a]
Yes	29 (29.3%)	23 (30.7%)	
**Rifampicine resistance**	N=29	N=23	
No	28 (96.6%)	23 (100%)	1.0000[b]
Yes	1 (3.4%)	0 (0%)	
Missing	70 (70.7%)	52 (69.3%)	

[a]: Chi-square test; [b]: Fisher exact test; [c]: t-test; [d]: Mann-Whitney U test.

Group	Numerator	Denominator	Proportion	Risk Difference	95% Confidence interval	p value
Control	29	99	29.3	0.9%	(-12.0% to 13.8%)	0.8899
Intervention	23	75	30.7			

Figure S25. Per-Protocol Population

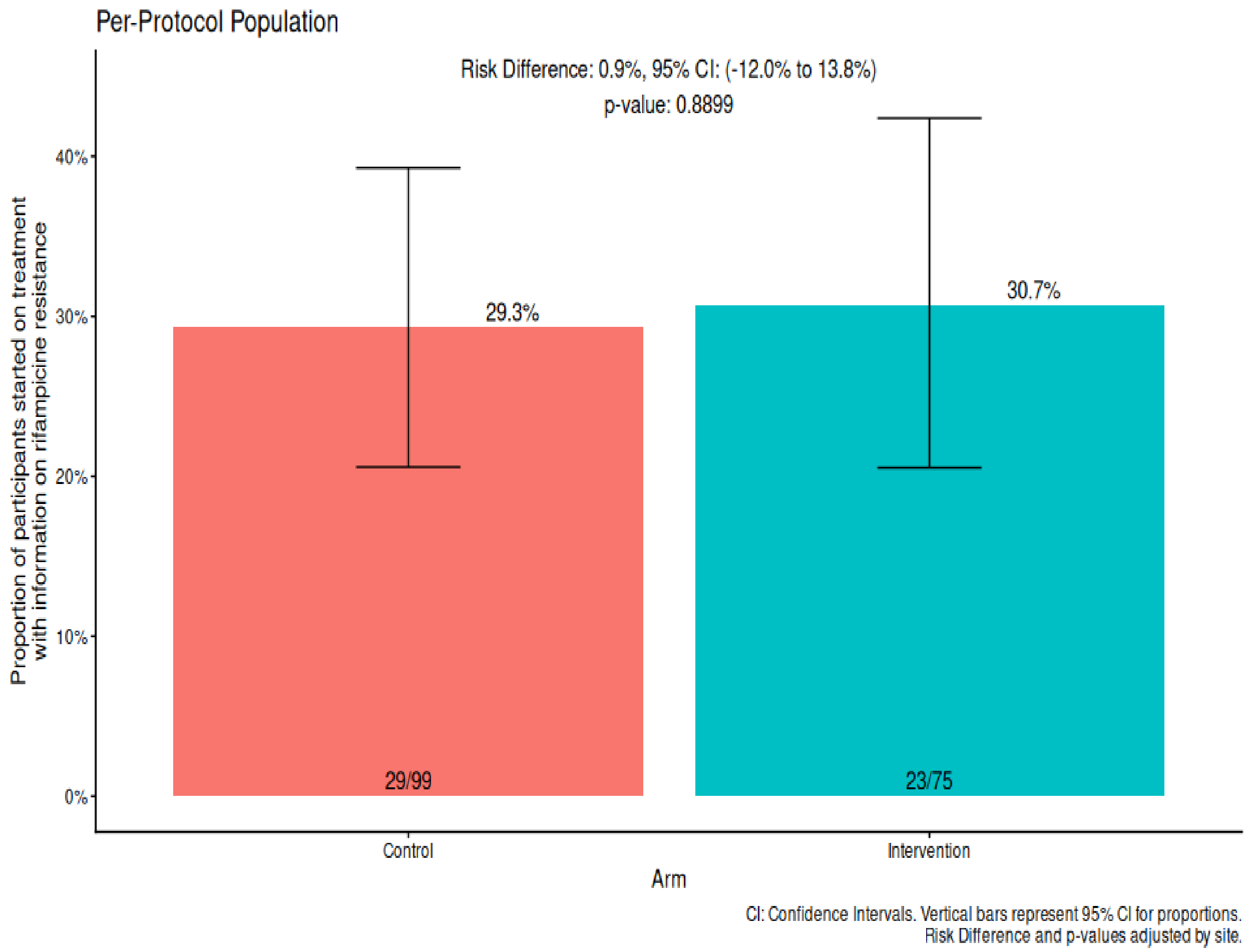

Subgroup analysis: Main Endpoint

Figure S26. Subgroup Analysis by CD4 Count

Facet	Group	Numerator	Denominator	Proportion	Risk Difference	95% Confidence interval	p value for interaction
<100	Control	44	158	27.8	-1.6%	(-11.6% to 8.4%)	0.7284
<100	Intervention	41	152	27.0			
100-200	Control	19	103	18.4	0.1%	(-10.3% to 10.5%)	
100-200	Intervention	17	101	16.8			
≥200	Control	27	302	8.9	1.5%	(-3.1% to 6.1%)	
≥200	Intervention	31	306	10.1			

By CD4 categories (cells/mm3)

p-value for interaction: 0.7284

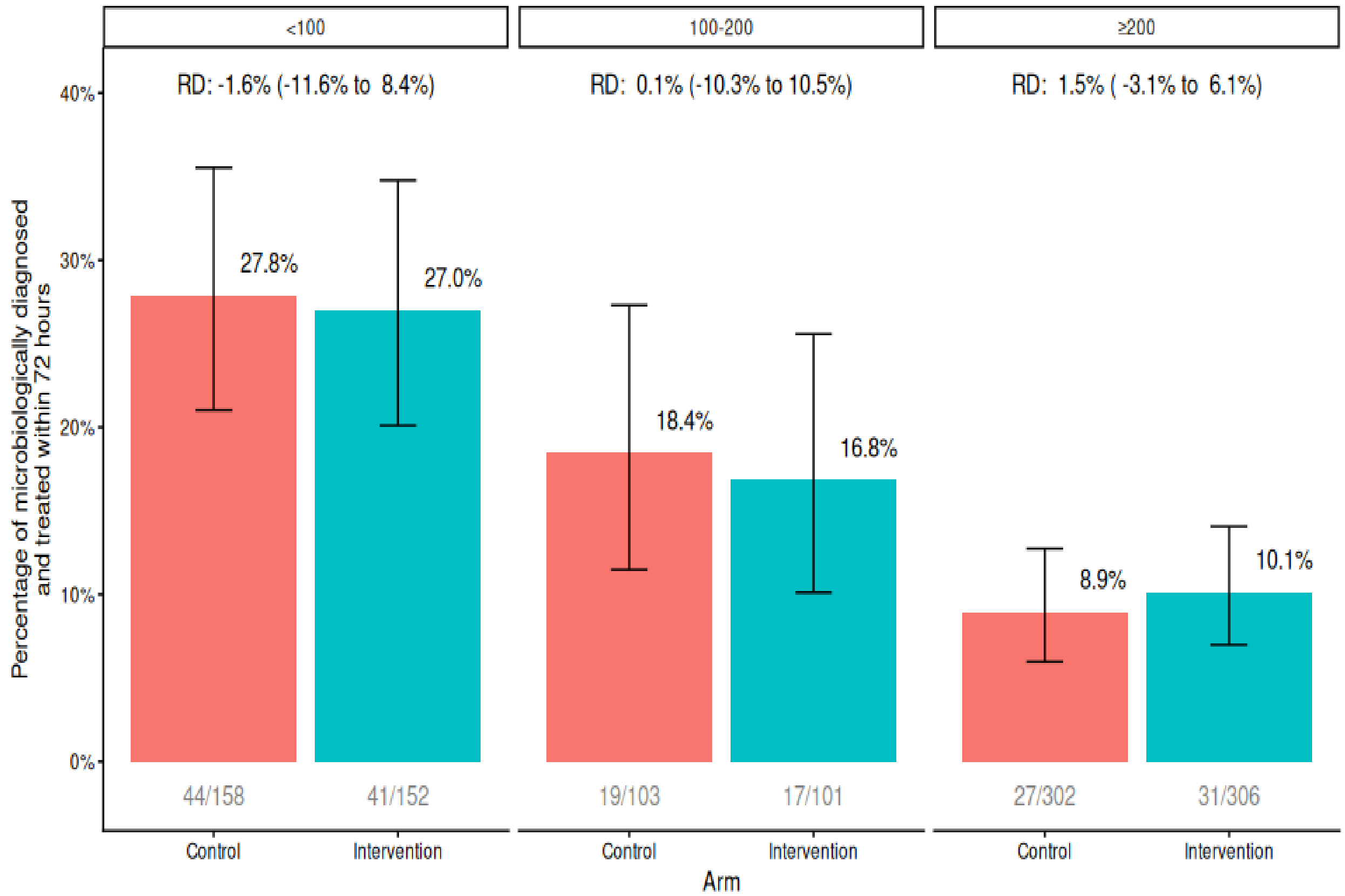

RD: stratified Risk Difference and 95% Confidence Intervals, adjusted by site.

Figure S27. Subgroup Analysis by Country

Facet	Group	Numerator	Denominator	Proportion	Risk Difference	95% Confidence interval	p value for interaction
Mozambique	Control	60	294	20.4	0.4%	(-6.1% to 7.0%)	0.7962
Mozambique	Intervention	61	292	20.9			
Tanzania	Control	30	296	10.1	1.1%	(-3.9% to 6.0%)	
Tanzania	Intervention	32	290	11.0			

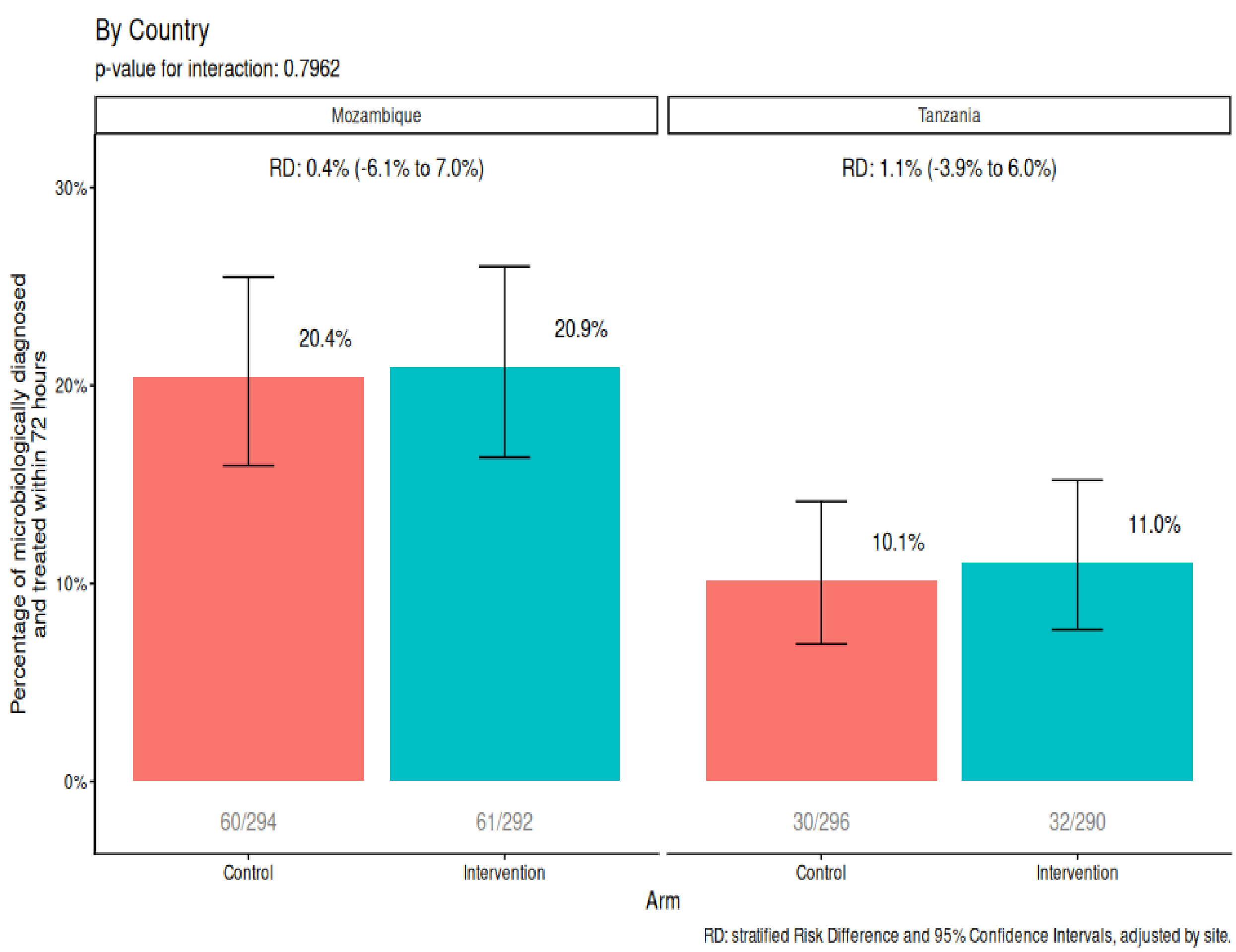

Figure S28. Subgroup Analysis by Institution

Facet	Group	Numerator	Denominator	Proportion	Risk Difference	95% Confidence interval	p-value for interaction
CISM	Control	22	133	16.5	5.1%	(-4.5% to 14.6%)	0.5935
CISM	Intervention	27	125	21.6			
IHI	Control	22	156	14.1	0.2%	(-7.7% to 8.0%)	
IHI	Intervention	21	147	14.3			
INS	Control	38	161	23.6	-3.2%	(-12.2% to 5.7%)	
INS	Intervention	34	167	20.4			
NIMR	Control	8	140	5.7	2.0%	(-3.8% to 7.8%)	
NIMR	Intervention	11	143	7.7			

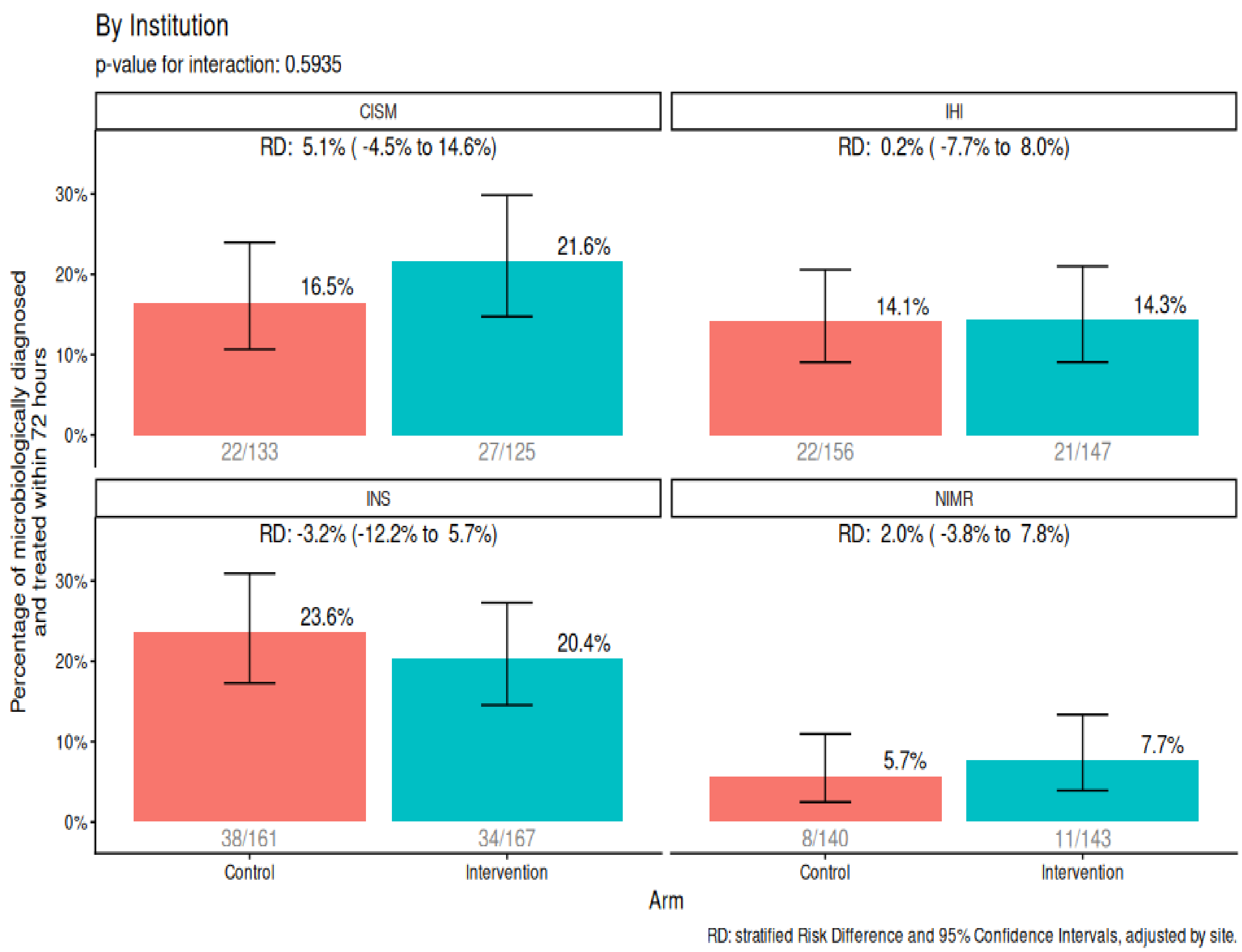
By Institution
p-value for interaction: 0.5935
CISM
RD: 5.1% (-4.5% to 14.6%)
16.5%
21.6%
22/133
27/125
IHI
RD: 0.2% (-7.7% to 8.0%)
14.1%
14.3%
22/156
21/147
INS
RD: -3.2% (-12.2% to 5.7%)
23.6%
20.4%
38/161
34/167
NIMR
RD: 2.0% (-3.8% to 7.8%)
5.7%
7.7%
8/140
11/143
30%
20%
10%
0%
Percentage of microbiologically diagnosed and treated within 72 hours
Control
Intervention
Arm
RD: stratified Risk Difference and 95% Confidence Intervals, adjusted by site.

Figure S29. Subgroup Analysis by Viral Load

Facet	Group	Numerator	Denominator	Proportion	Risk Difference	95% Confidence interval	p-value for interaction
Detectable (>150 copies)	Control	56	233	24.0	-5.5%	(-12.9% to 1.9%)	0.0340
Detectable (>150 copies)	Intervention	42	225	18.7			
Undetectable (≤150 copies)	Control	31	313	9.9	4.0%	(-1.0% to 9.0%)	
Undetectable (≤150 copies)	Intervention	42	311	13.5			

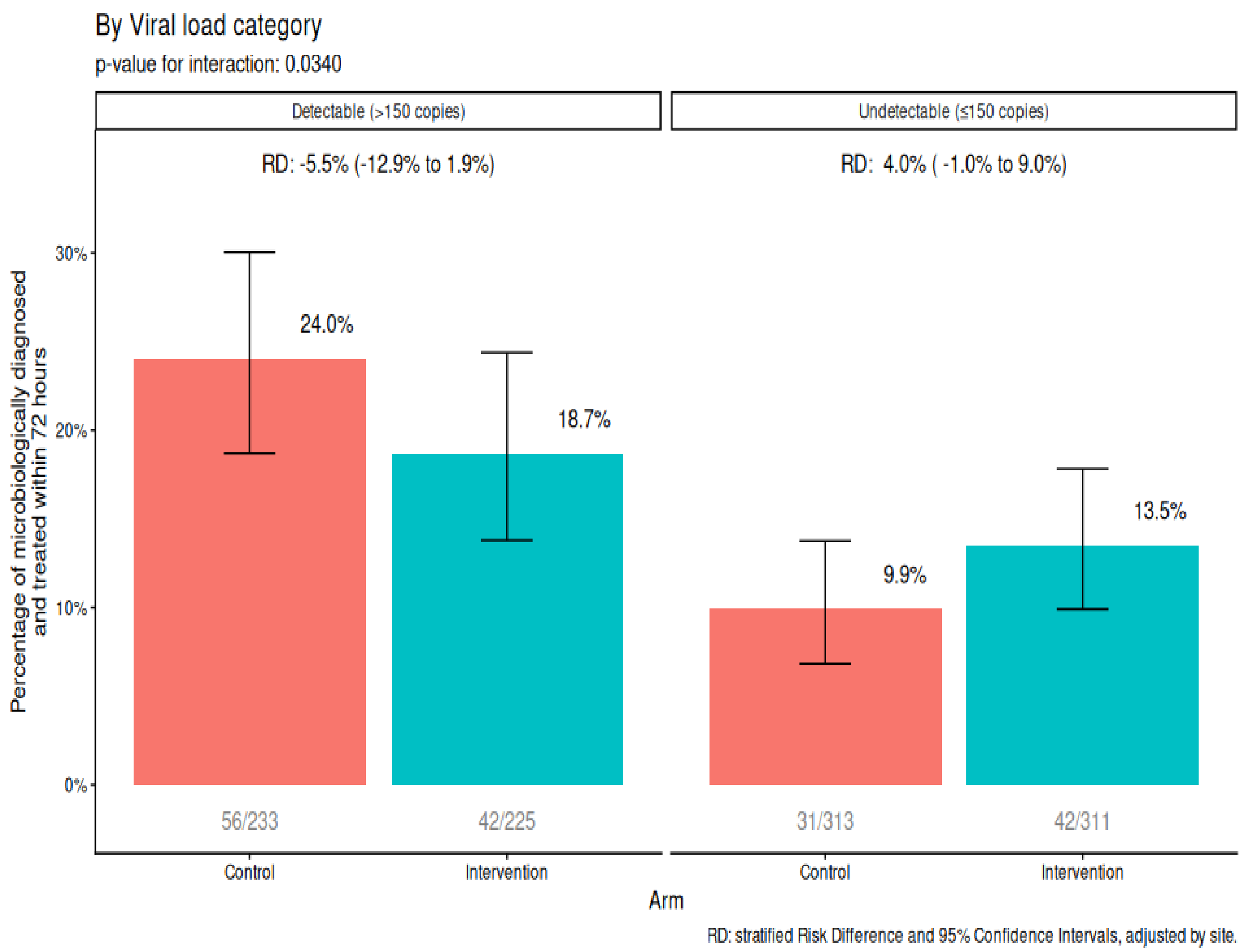

Figure S30. Subgroup Analysis by Sex

Facet	Group	Numerator	Denominator	Proportion	Risk Difference	95% Confidence interval	p-value for interaction
Female	Control	48	366	13.1	-0.1%	(-5.0% to 4.7%)	0.7260
Female	Intervention	45	349	12.9			
Male	Control	42	224	18.8	1.6%	(-5.6% to 8.8%)	
Male	Intervention	48	233	20.6			

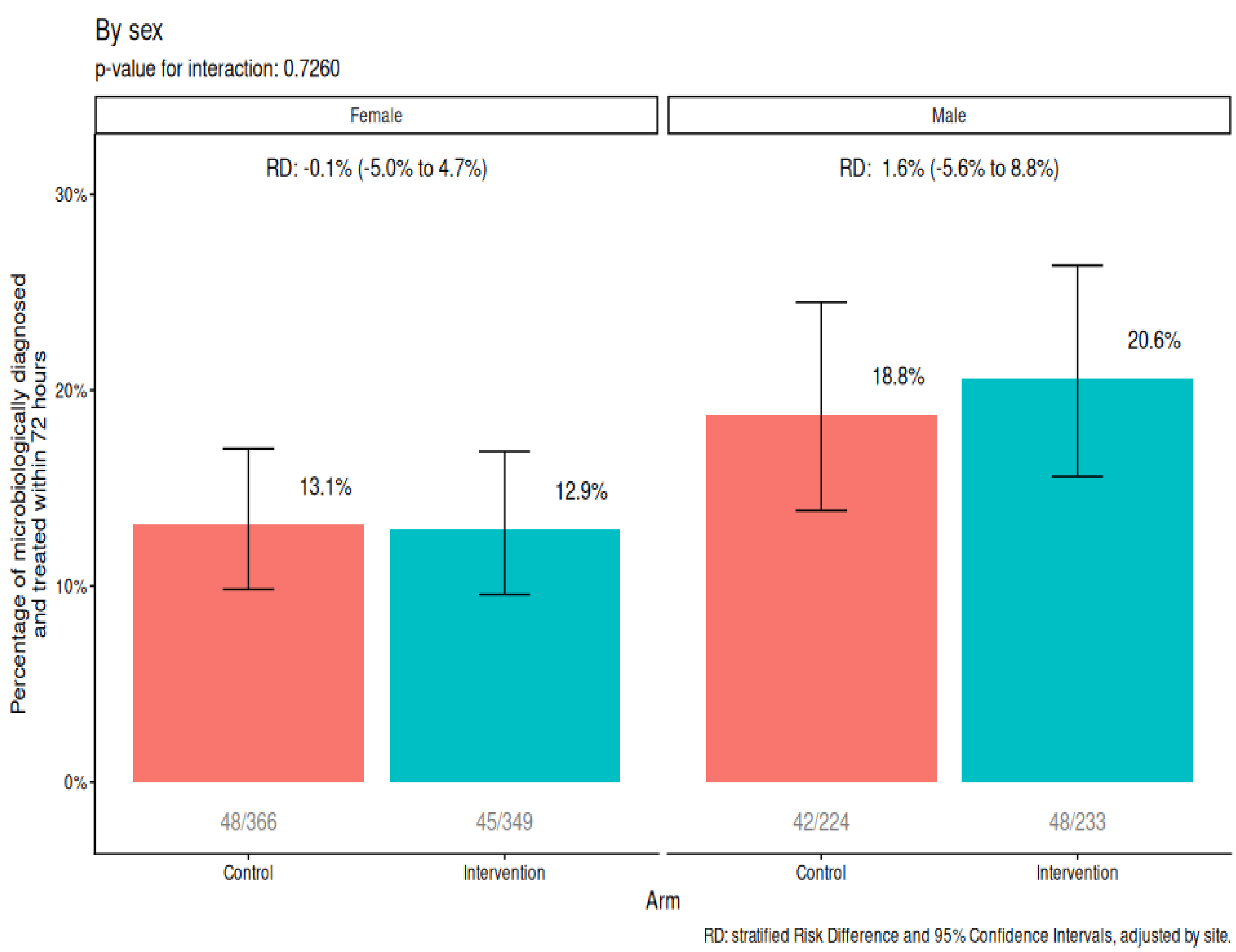

Subgroup analysis: Main Secondary Endpoint

Figure S31. Subgroup Analysis by CD4 Count

Facet	Group	Numerator	Denominator	Proportion	Risk Difference	95% Confidence interval	p-value for interaction
<100	Control	57	158	36.1	-1.2%	(-11.9% to 9.5%)	0.1968
<100	Intervention	50	152	32.9			
100-200	Control	36	103	35.0	5.5%	(-7.7% to 18.8%)	
100-200	Intervention	41	101	40.6			
≥200	Control	72	302	23.8	-6.5%	(-12.9% to 0.0%)	
≥200	Intervention	53	306	17.3			

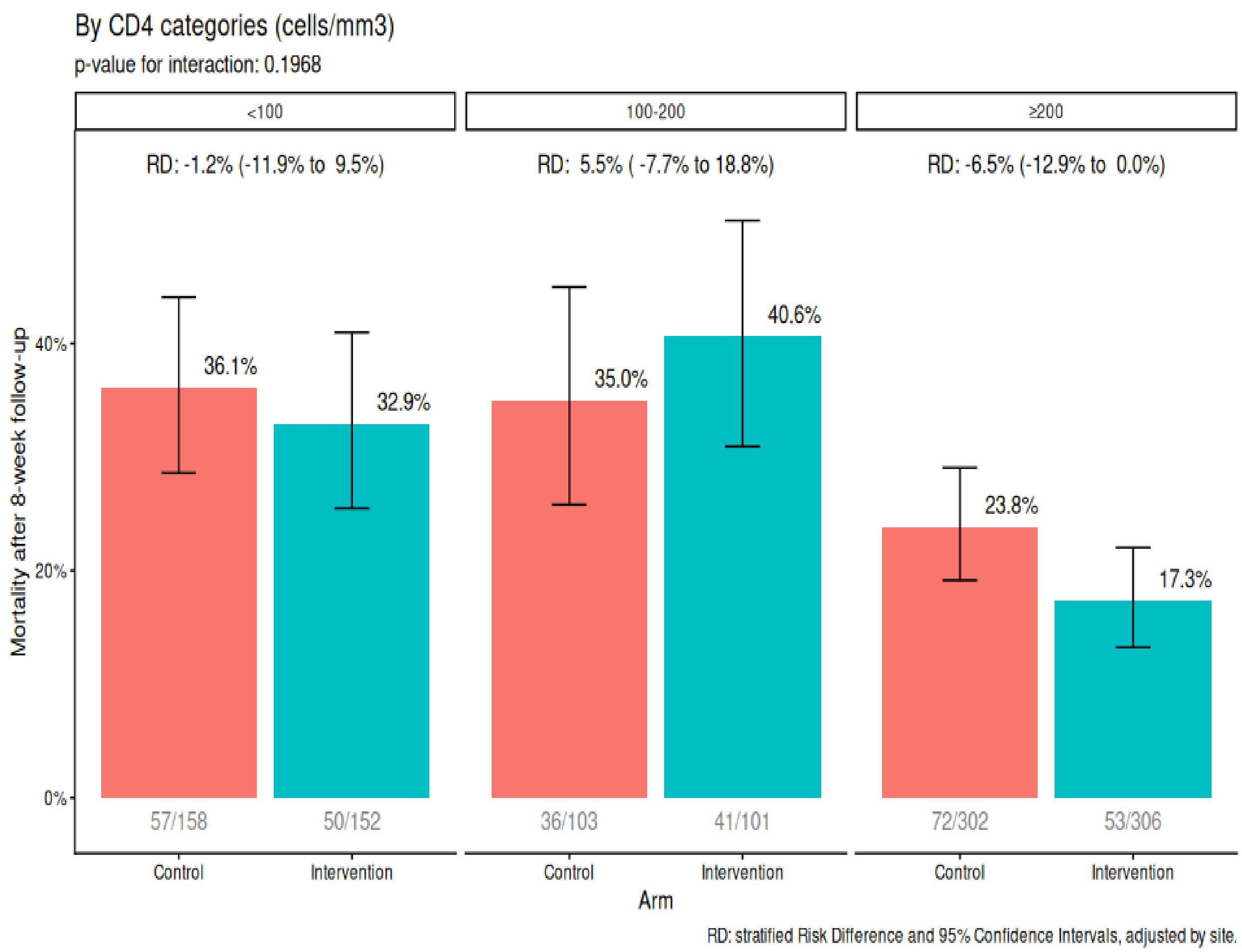
By CD4 categories (cells/mm3)
p-value for interaction: 0.1968
<100
100-200
≥200
RD: -1.2% (-11.9% to 9.5%)
RD: 5.5% (-7.7% to 18.8%)
RD: -6.5% (-12.9% to 0.0%)
Mortality after 8-week follow-up
40%
20%
0%
36.1%
32.9%
35.0%
40.6%
23.8%
17.3%
57/158
50/152
36/103
41/101
72/302
53/306
Control
Intervention
Control
Intervention
Control
Intervention
Arm
RD: stratified Risk Difference and 95% Confidence Intervals, adjusted by site.

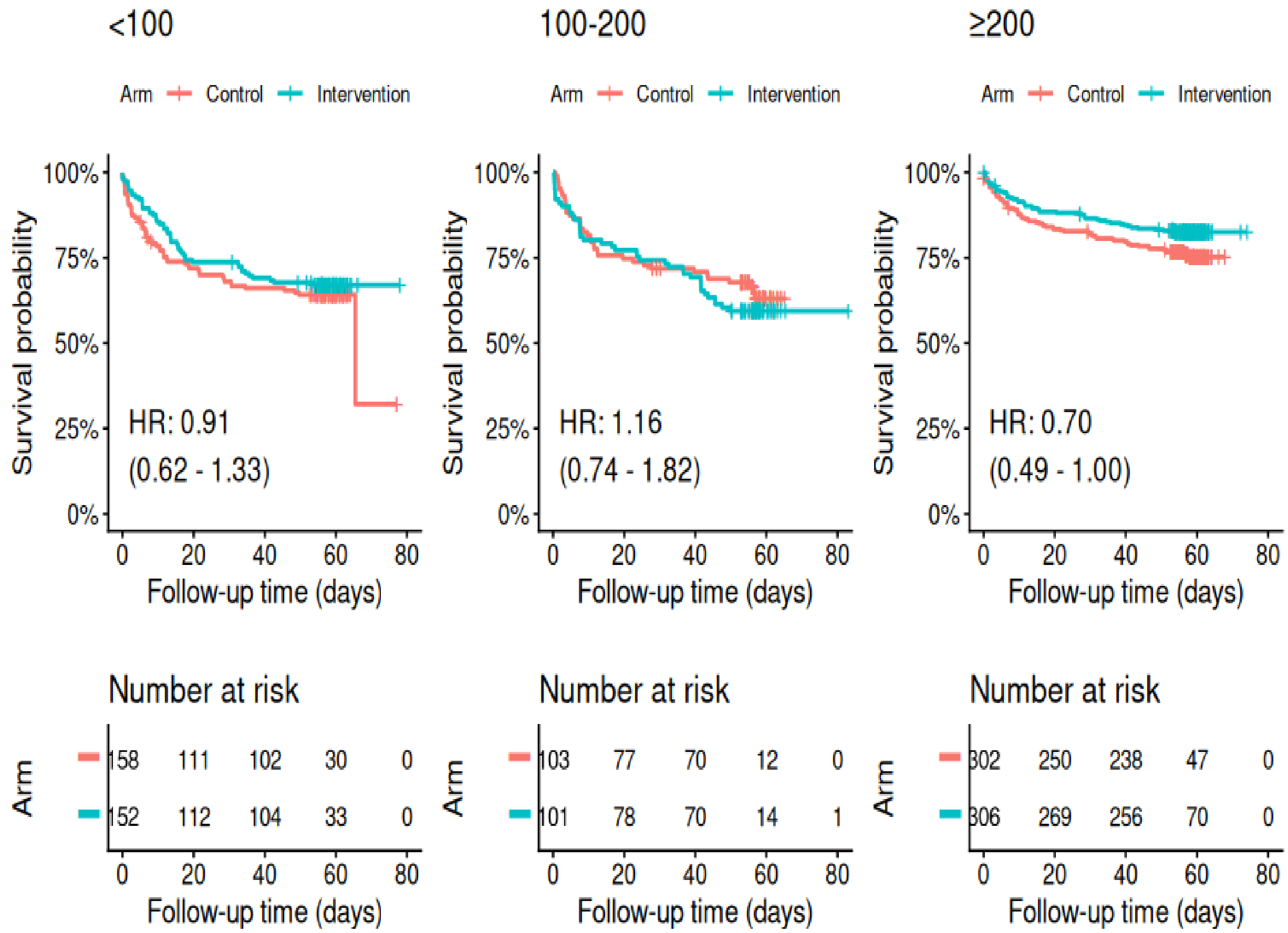

p-value for interaction: 0.2280. HR: stratified Hazard Ratio and 95% Confidence Intervals, adjusted by site.

Figure S32. Subgroup Analysis by Country

Facet	Group	Numerator	Denominator	Proportion	Risk Difference	95% Confidence interval	p-value for interaction
Mozambique	Control	83	294	28.2	-4.0%	(-11.1% to 3.2%)	0.7003
Mozambique	Intervention	71	292	24.3			
Tanzania	Control	87	296	29.4	-2.1%	(-9.4% to 5.2%)	
Tanzania	Intervention	79	290	27.2			

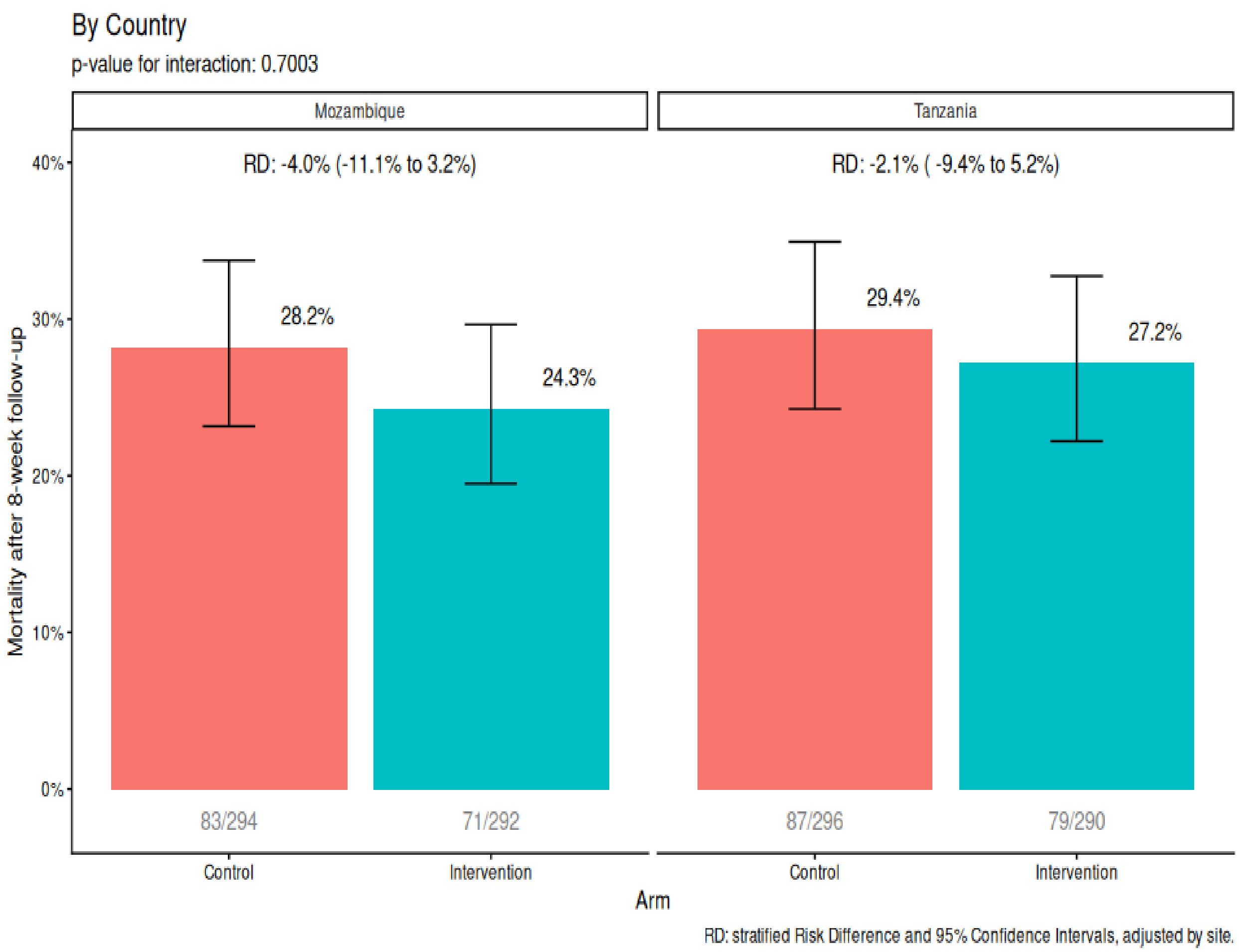

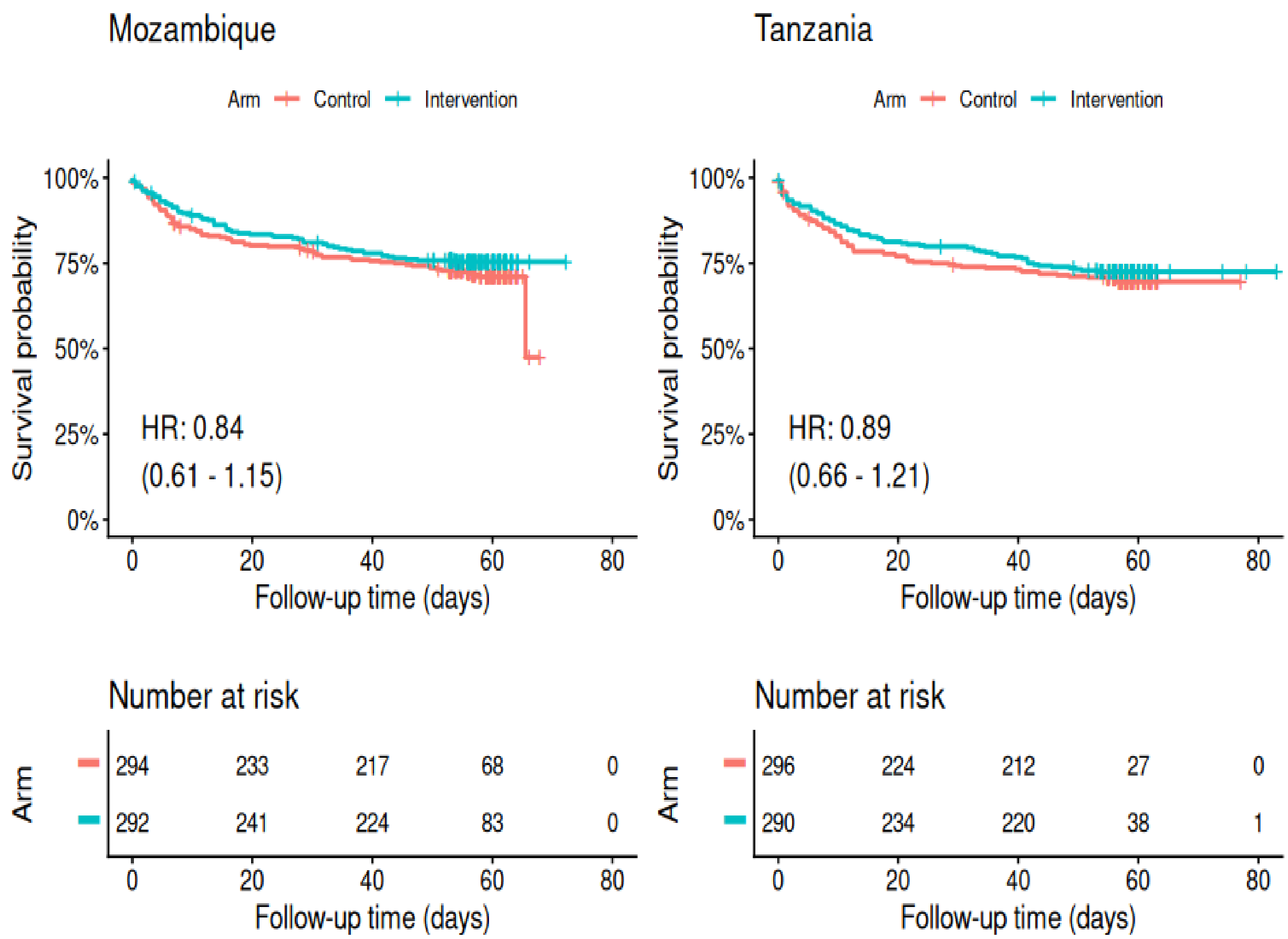

p-value for interaction: 0.7891. HR: stratified Hazard Ratio and 95% Confidence Intervals, adjusted by site.

Figure S33. Subgroup Analysis by Institution

facet	Group	Numerator	Denominator	Proportion	Risk Difference	95% Confidence interval	p-value for interaction
CISM	Control	38	133	28.6	-7.0%	(-17.5% to 3.6%)	0.8587
CISM	Intervention	27	125	21.6			
IHI	Control	47	156	30.1	-1.6%	(-11.8% to 8.7%)	
IHI	Intervention	42	147	28.6			
INS	Control	45	161	28.0	-1.6%	(-11.2% to 8.0%)	
INS	Intervention	44	167	26.3			
NIMR	Control	40	140	28.6	-2.7%	(-13.1% to 7.7%)	
NIMR	Intervention	37	143	25.9			

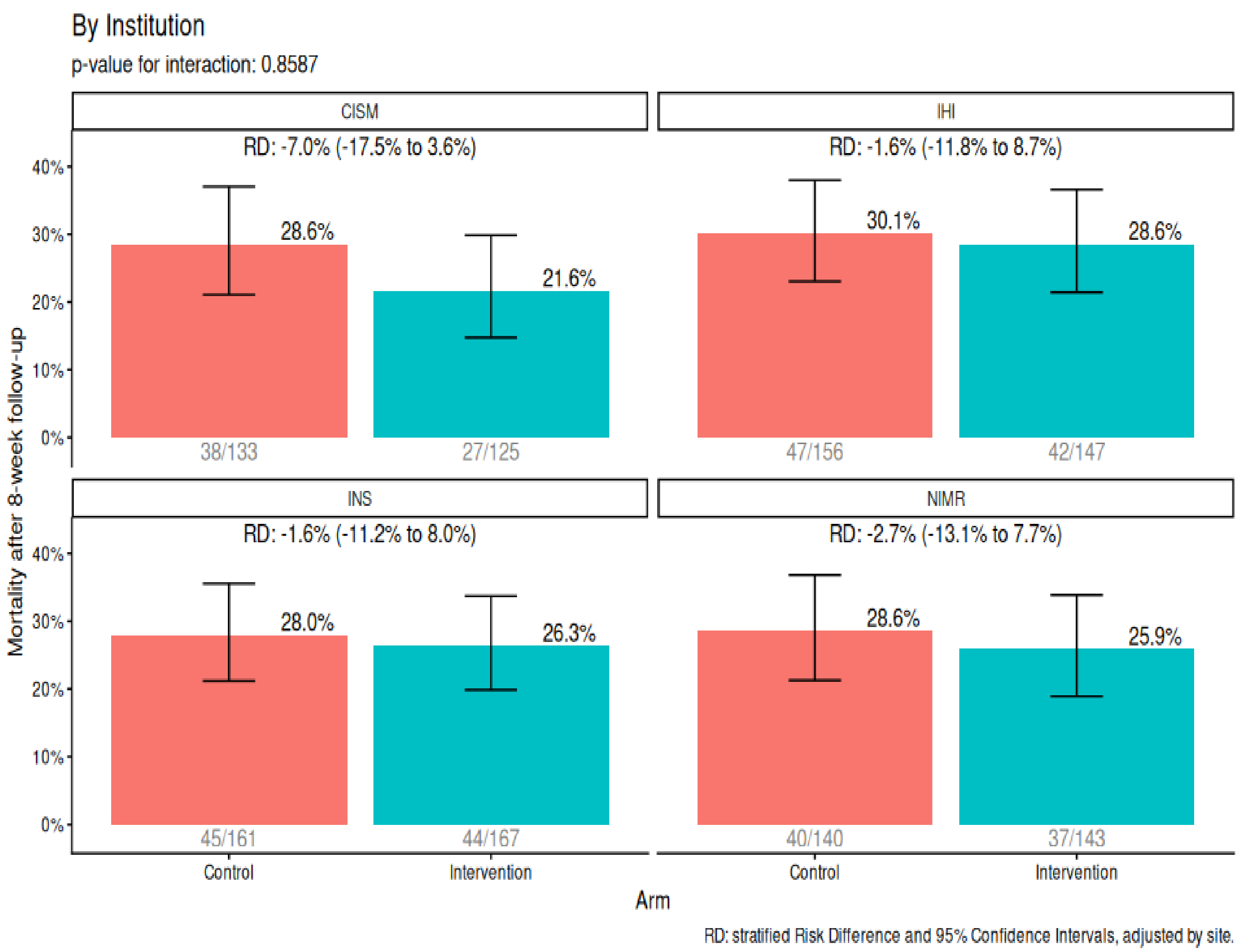
By Institution
p-value for interaction: 0.8587
CISM
RD: -7.0% (-17.5% to 3.6%)
28.6%
21.6%
38/133
27/125
IHI
RD: -1.6% (-11.8% to 8.7%)
30.1%
28.6%
47/156
42/147
INS
RD: -1.6% (-11.2% to 8.0%)
28.0%
26.3%
45/161
44/167
NIMR
RD: -2.7% (-13.1% to 7.7%)
28.6%
25.9%
40/140
37/143
Mortality after 8-week follow-up
40%
30%
20%
10%
0%
Control
Intervention
Arm
RD: stratified Risk Difference and 95% Confidence Intervals, adjusted by site.

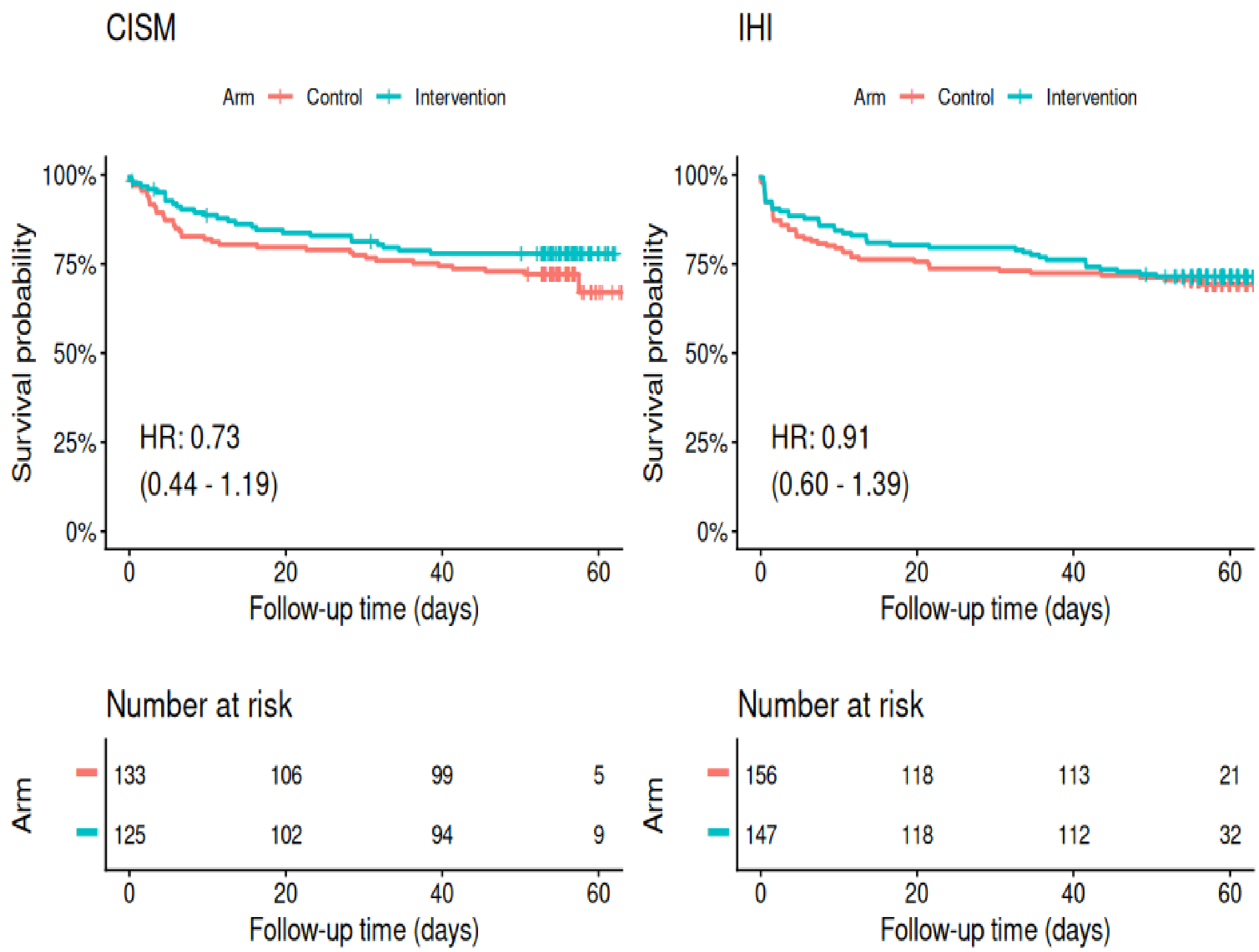
CISM
Arm Control Intervention
Survival probability
100%
75%
50%
25%
0%
HR: 0.73
(0.44 - 1.19)
0
20
40
60
Follow-up time (days)
Number at risk
Arm
133
106
99
5
125
102
94
9
0
20
40
60
Follow-up time (days)
IHI
Arm Control Intervention
Survival probability
100%
75%
50%
25%
0%
HR: 0.91
(0.60 - 1.39)
0
20
40
60
Follow-up time (days)
Number at risk
Arm
156
118
113
21
147
118
112
32
0
20
40
60
Follow-up time (days)

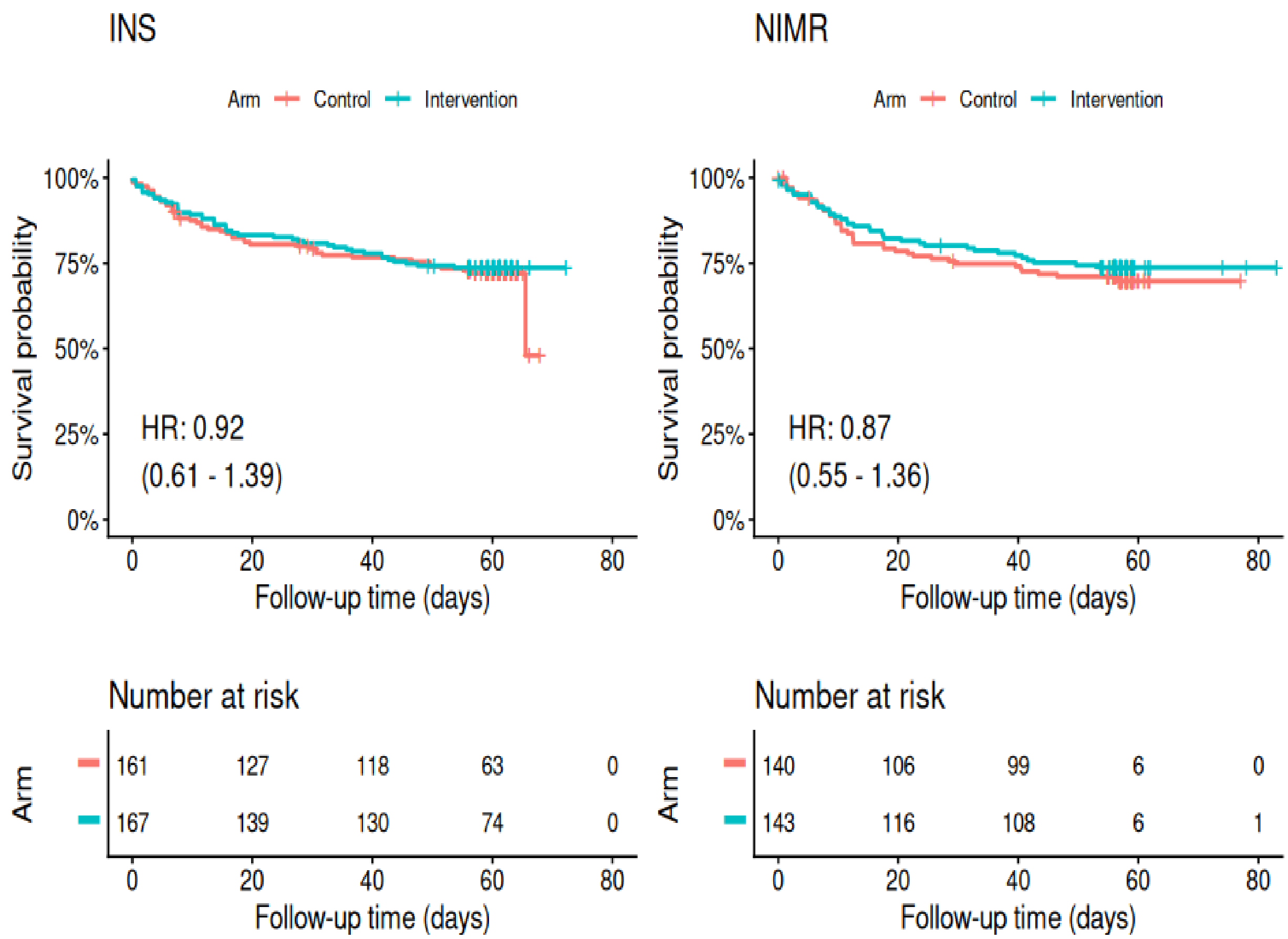

p-value for interaction: 0.8987. HR: stratified Hazard Ratio and 95% Confidence Intervals, adjusted by site.

Figure S34. Subgroup Analysis by Viral Load

facet	Group	Numerator	Denominator	Proportion	Risk Difference	95% Confidence interval	p-value for interaction
Detectable (>150 copies)	Control	75	233	32.2	-8.1%	(-16.2% to 0.1%)	0.2119
Detectable (>150 copies)	Intervention	54	225	24.0			
Undetectable (≤150 copies)	Control	83	313	26.5	-1.0%	(-7.9% to 5.8%)	
Undetectable (≤150 copies)	Intervention	79	311	25.4			

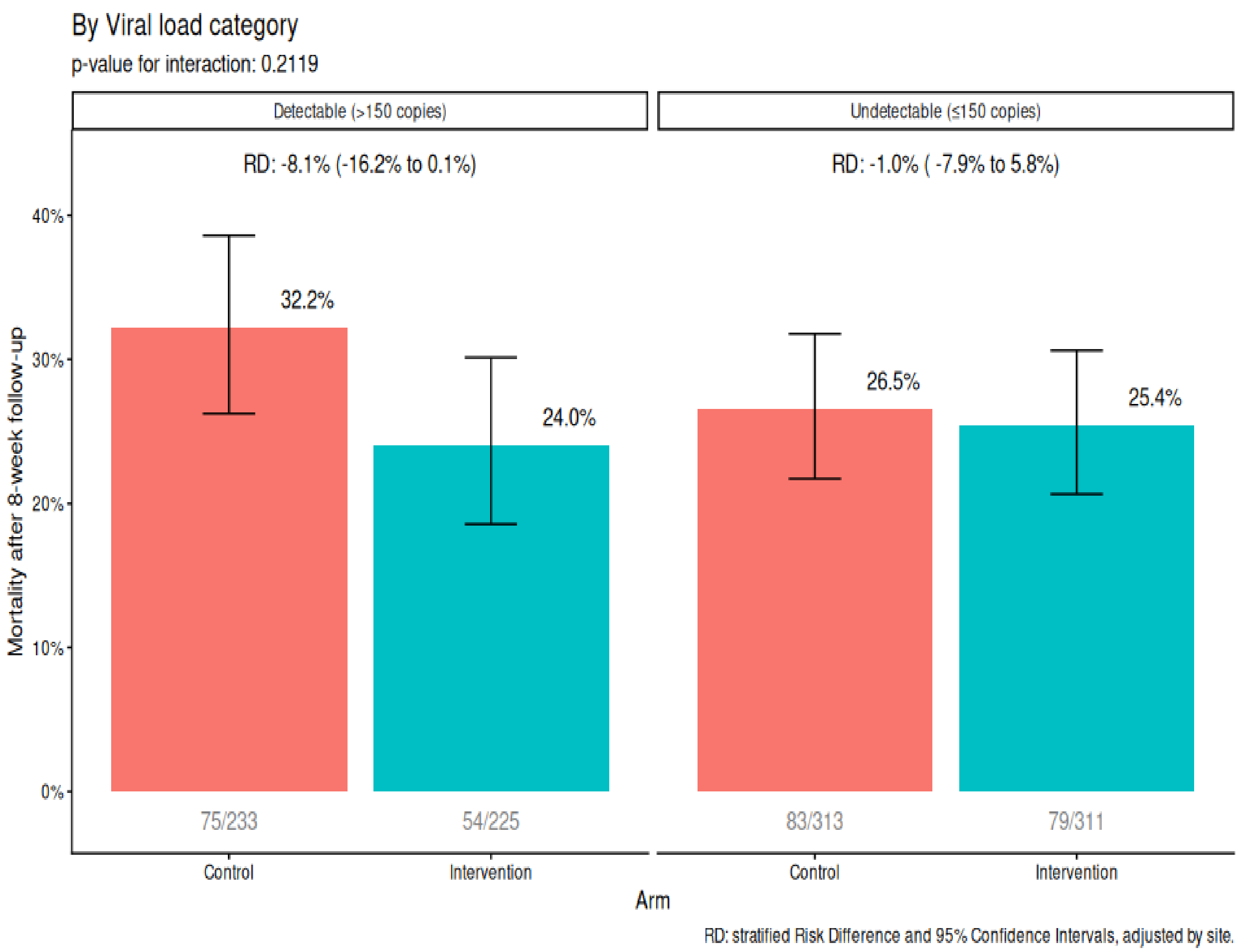

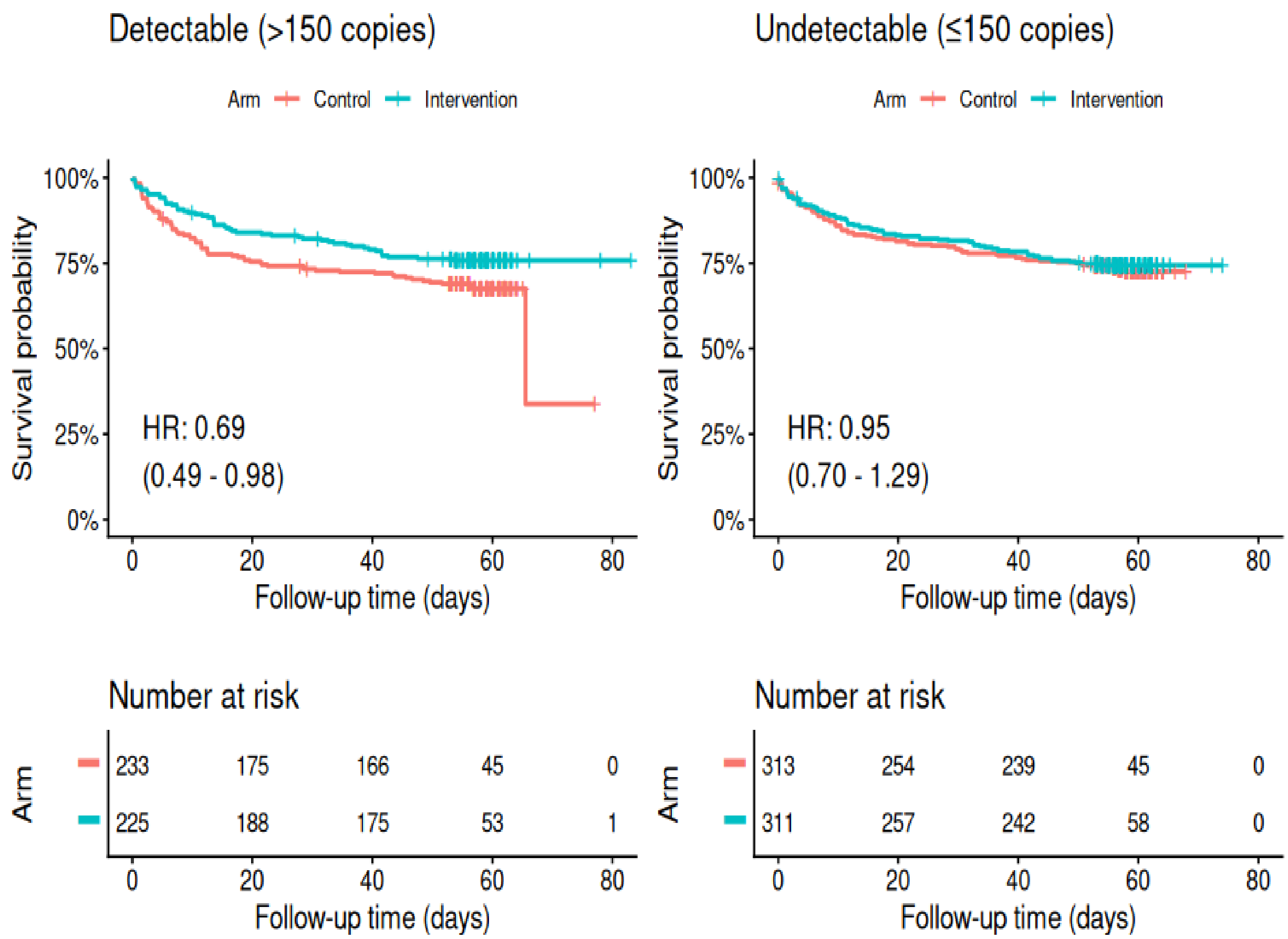

p-value for interaction: 0.1912. HR: stratified Hazard Ratio and 95% Confidence Intervals, adjusted by site.

Figure S35. Subgroup Analysis by Sex

facet	Group	Numerator	Denominator	Proportion	Risk Difference	95% Confidence interval	p-value for interaction
Female	Control	101	366	27.6	-4.8%	(-11.2% to 1.5%)	0.3553
Female	Intervention	79	349	22.6			
Male	Control	69	224	30.8	-0.3%	(-8.7% to 8.2%)	
Male	Intervention	71	233	30.5			

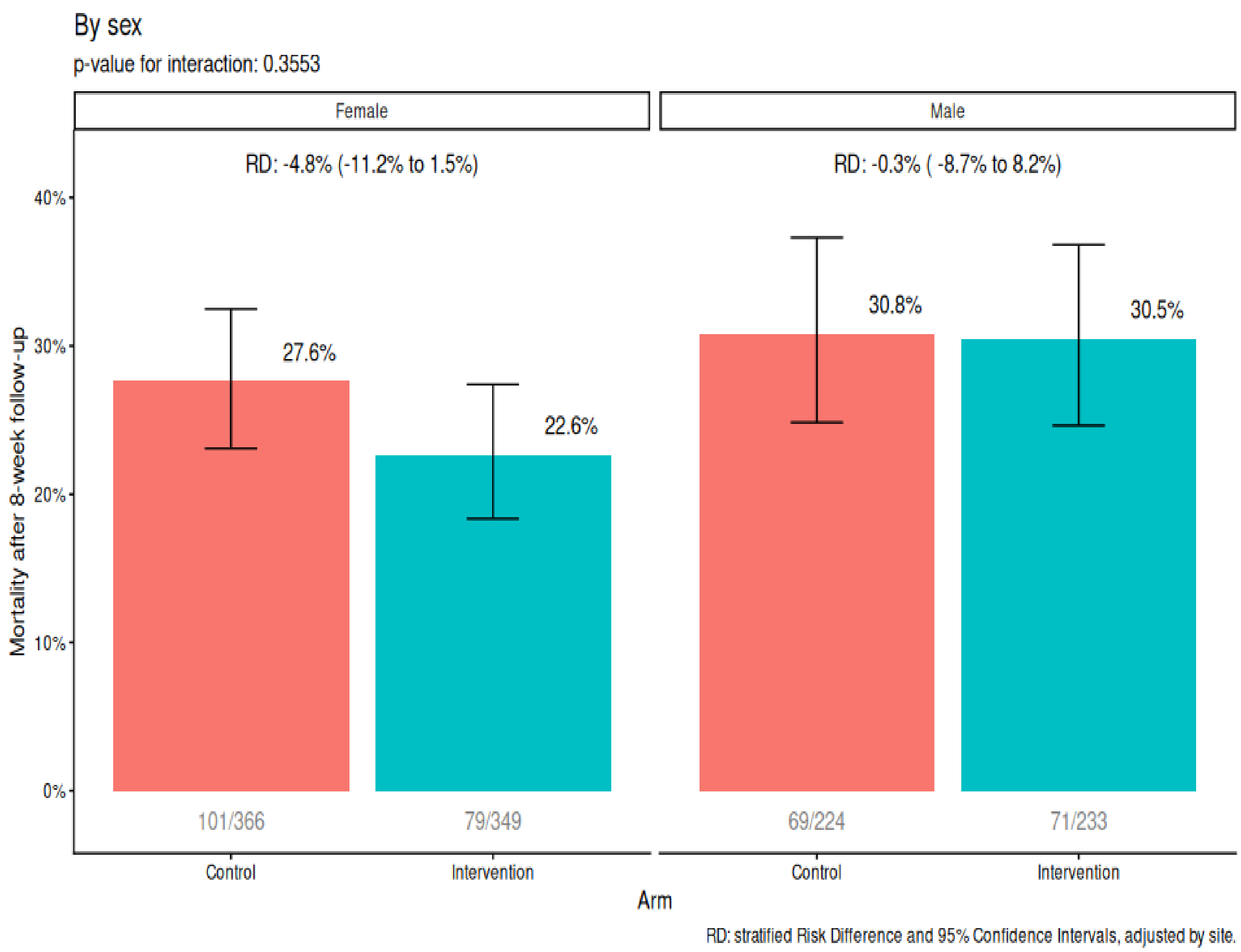

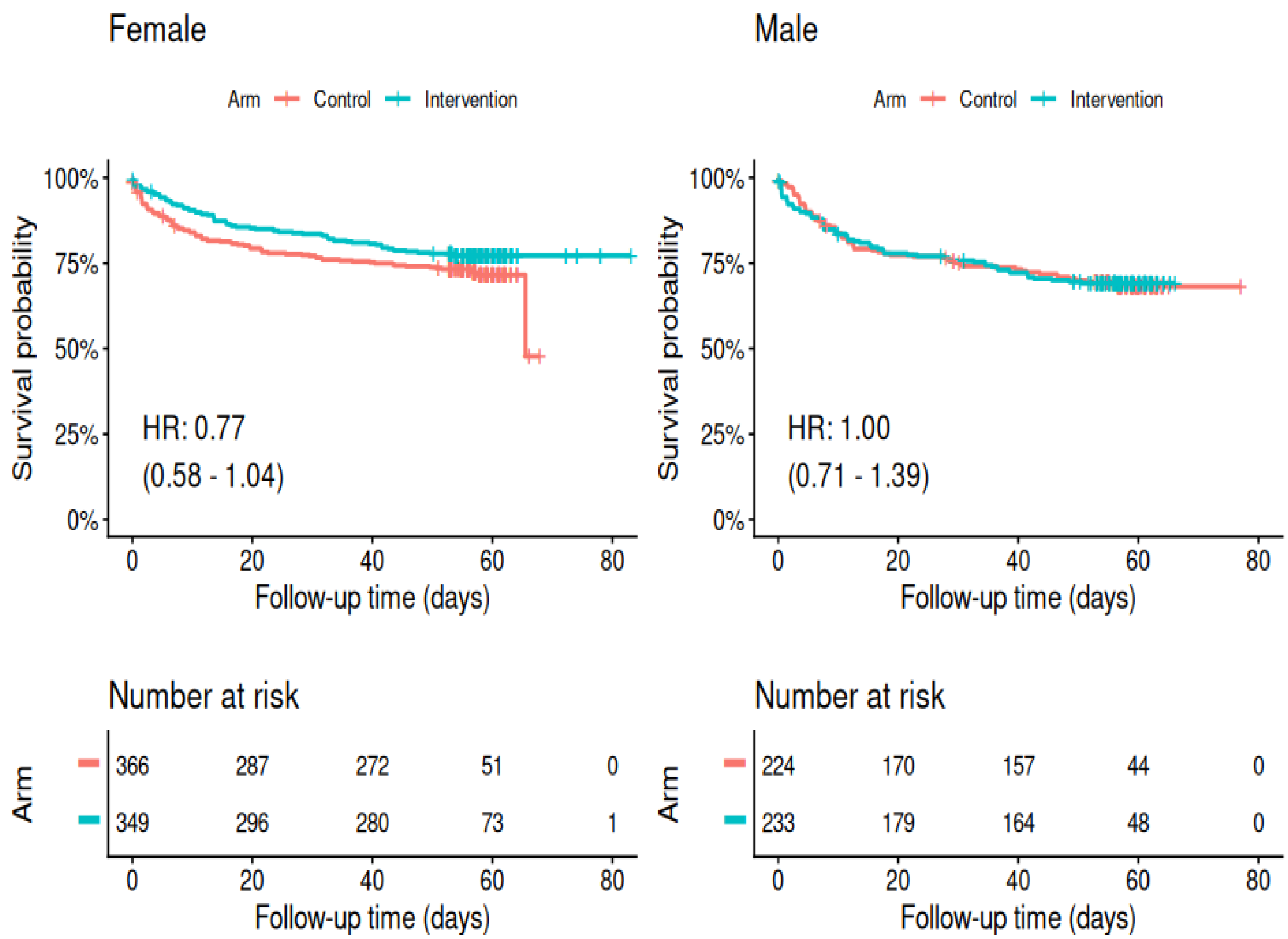

p-value for interaction: 0.2596. HR: stratified Hazard Ratio and 95% Confidence Intervals, adjusted by site.

Post hoc analyses

Figure S36. Mortality of only LF-LAM+ TB cases, with and without treatment

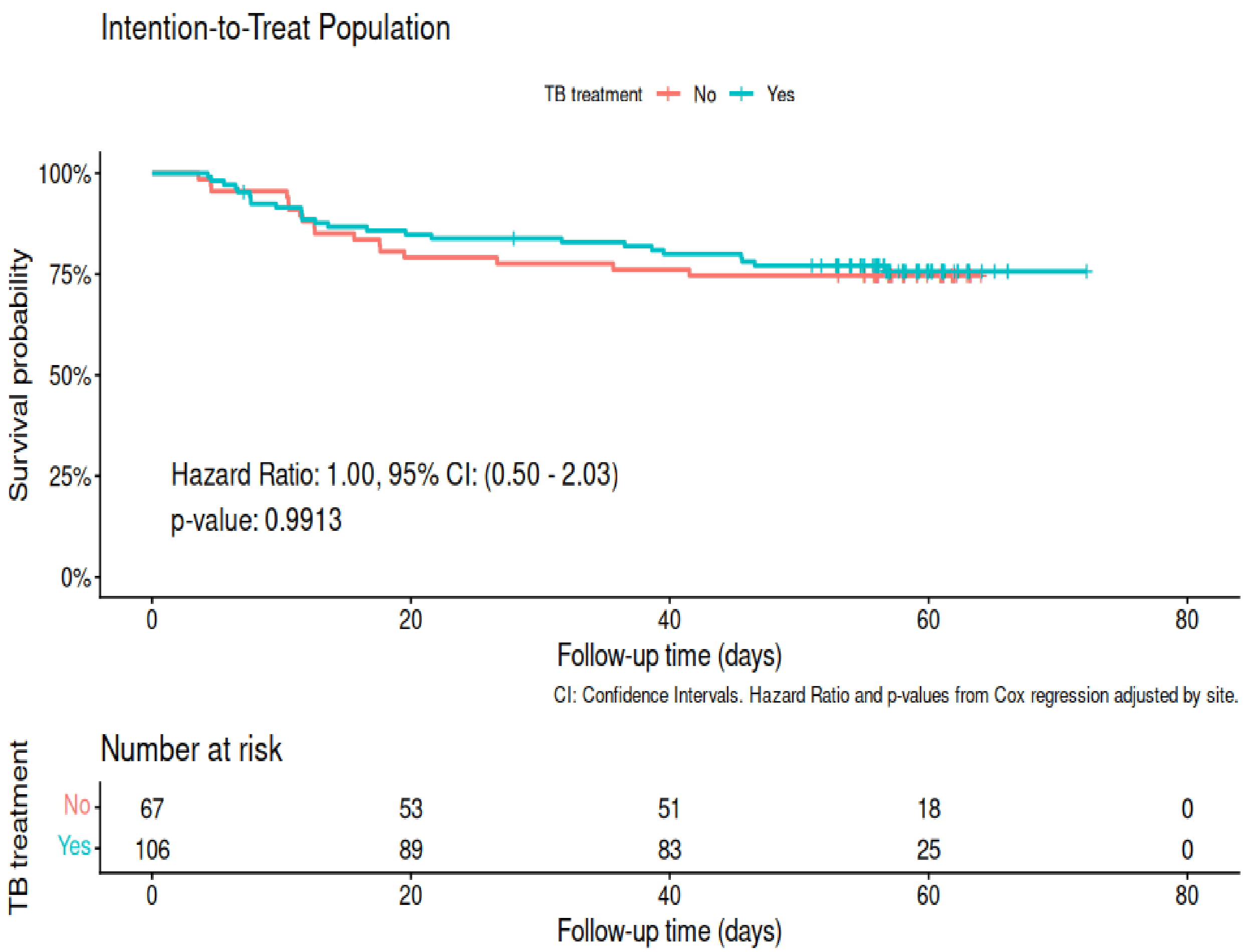

Group	Numerator	Denominator	Proportion	Risk Difference	95% Confidence interval	p-value
No	17	67	25.4	0.1%	(-14.8% to 15.1%)	0.9868
Yes	25	106	23.6			

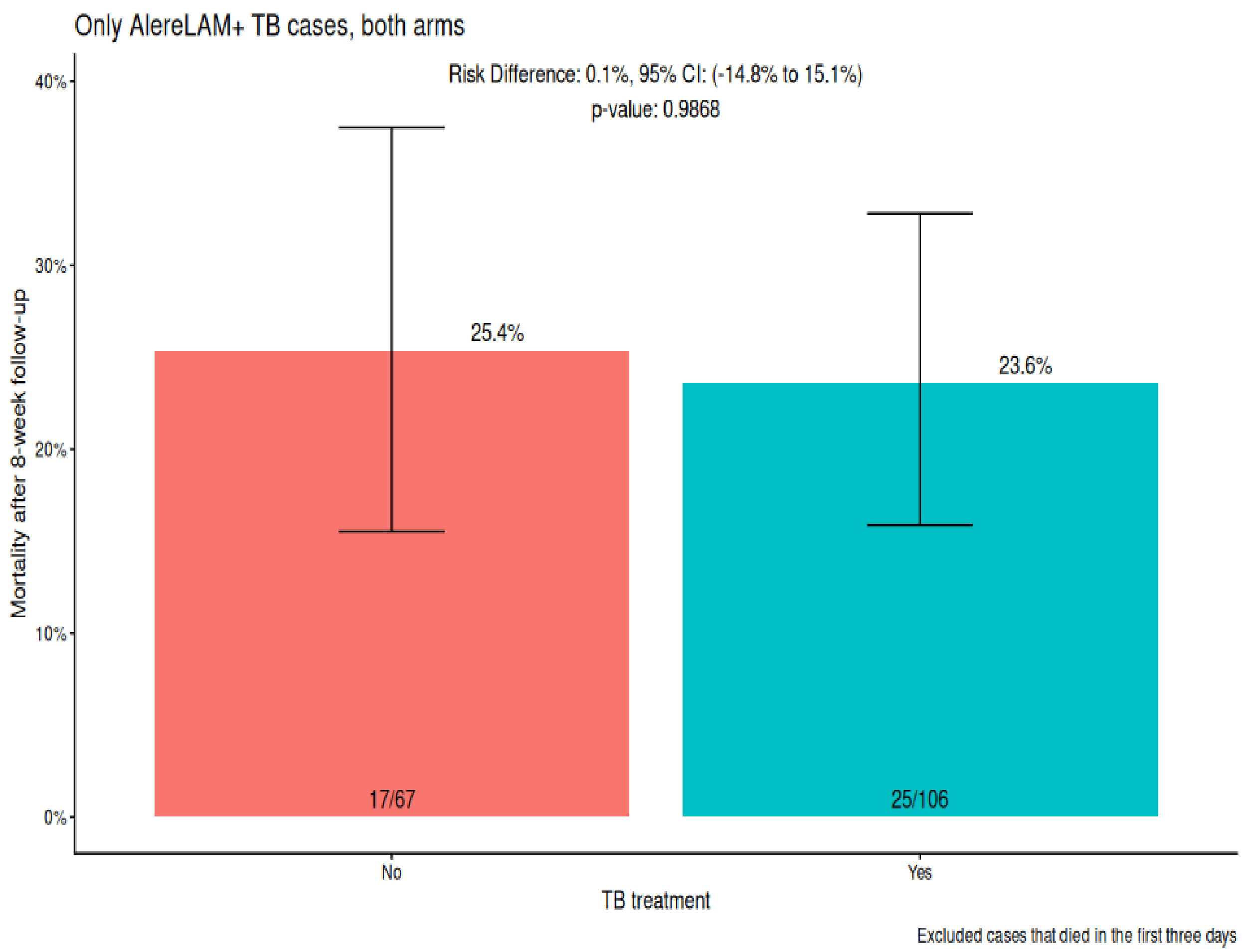
Only AlereLAM+ TB cases, both arms
Risk Difference: 0.1%, 95% CI: (-14.8% to 15.1%)
p-value: 0.9868
Mortality after 8-week follow-up
40%
30%
20%
10%
0%
25.4%
23.6%
17/67
25/106
No
Yes
TB treatment
Excluded cases that died in the first three days

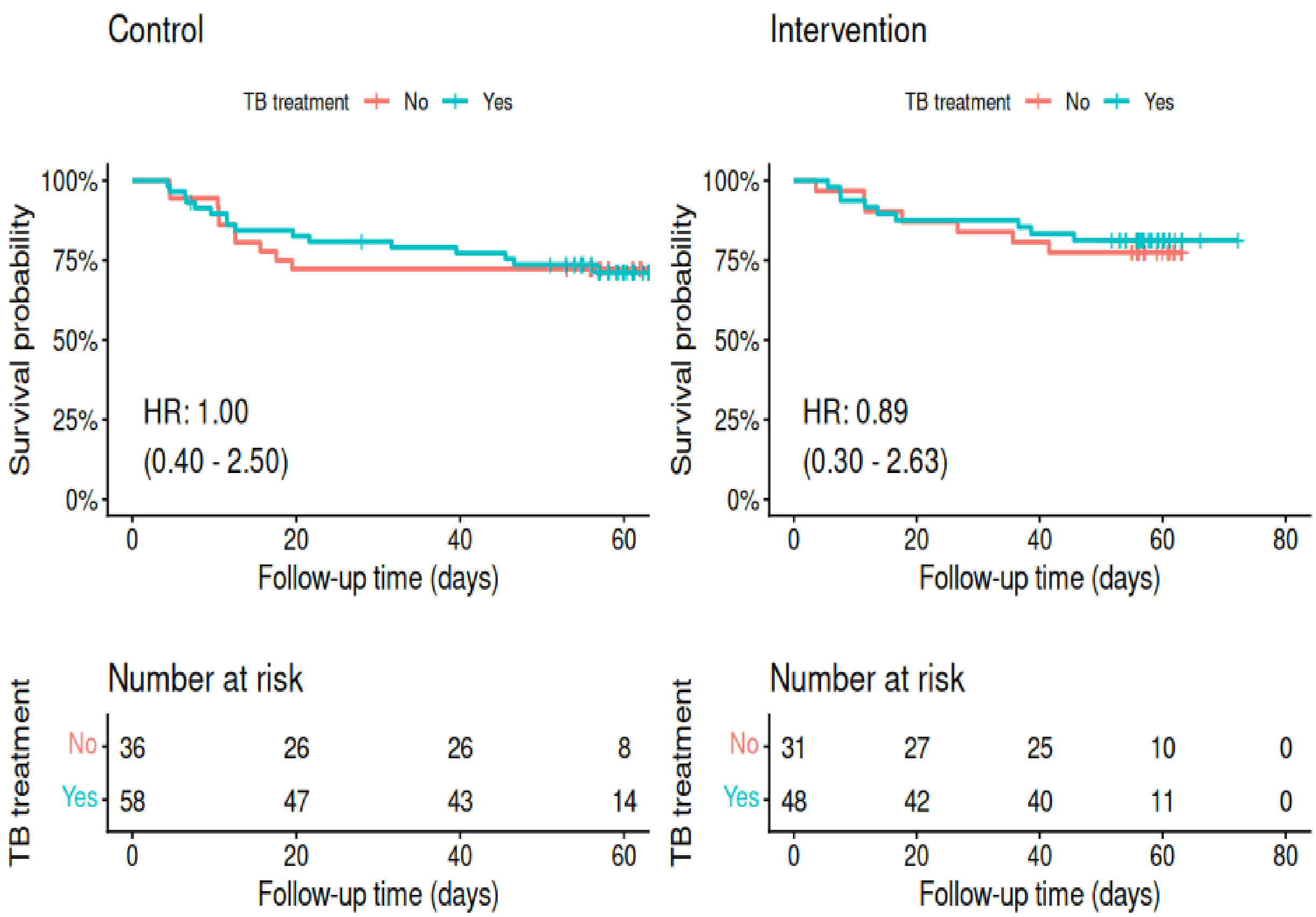

Survival of TB cases only positive on AlereLAM, treated vs. untreated
p-value for interaction: 0.8645. HR: stratified Hazard Ratio and 95% Confidence Intervals, adjusted by site.
Excluded cases that died in the first three days

Figure S37. Mortality of microbiologically confirmed TB cases, with and without treatment

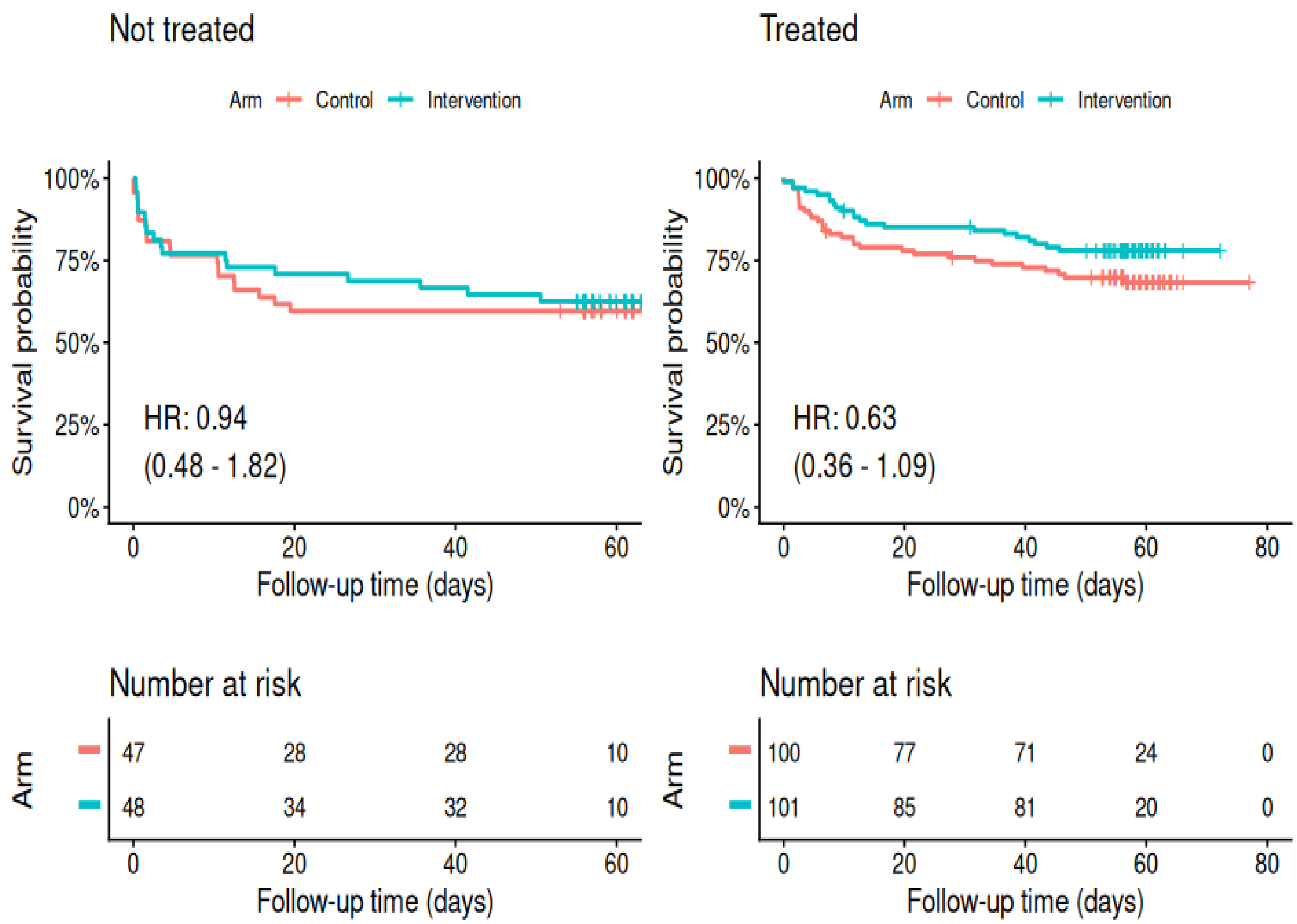

Survival of microbiologically confirmed TB cases, treated vs. untreated
p-value for interaction: 0.4560. HR: stratified Hazard Ratio and 95% Confidence Intervals, adjusted by site.
